# Structural Insights from Molecular Modeling of Isoindolin-1-One Derivatives as PI3Kγ Inhibitors against Gastric Carcinoma

**DOI:** 10.3390/biomedicines10040813

**Published:** 2022-03-30

**Authors:** Suparna Ghosh, Seung Joo Cho

**Affiliations:** 1 Department of Biomedical Sciences, College of Medicine, Chosun University, Gwangju 501-759, Korea; s.ghosh@chosun.kr; 2 Department of Cellular and Molecular Medicine, College of Medicine, Chosun University, Gwangju 501-759, Korea

**Keywords:** gastric carcinoma, PI3Kγ, PI3Kδ, tumor-associated macrophage, MM-PB/GBSA, CoMFA, CoMSIA, structure–activity relationship

## Abstract

The upregulation of phosphoinositol-3-kinase γ (PI3Kγ) is deemed to be positively correlated with tumor-associated-macrophage (TAM)-mediated gastric carcinoma (GC). PI3Kγ suppresses tumor necrosis factor-alpha (TNF-α) and interleukin-12 (IL-12) through activation of the AKT/mTOR pathway, which promotes the immunosuppressant phenotype of TAM. Unlike α and β isoforms, δ and γ isoforms are primarily distributed in leucocytes and macrophages. Dual inhibitors against PI3Kδ and PI3Kγ have been proven to have merits in targeting solid tumors. Furthermore, it has been found that PI3Kδ is activated by cytokines, while PI3Kγ is activated by G-protein-coupled receptors (GPCRs). This facilitates determining the functional difference between these two isoforms. For this goal, selective inhibitors would be immensely helpful. In the current manuscript, we conducted various molecular modeling studies with a series of isoindolin-1-one derivatives as potent PI3Kγ inhibitors by combining molecular docking, molecular dynamics (MD), molecular mechanics, Poisson–Boltzmann/generalized Born surface area (MM-PB/GBSA) binding free energy calculation, and three-dimensional structure–activity relationship (3D-QSAR) study. To evaluate the selectivity of γ isoform over δ, the molecular modeling studies of idelalisib analogs reported as PI3Kδ inhibitors were also investigated. The contour polyhedrons were generated from the comparative molecular field analysis (CoMFA) and comparative molecular similarity index analysis (CoMSIA) around the ligand-bound active site for both isoforms, which could emphasize plausible explanations for the physicochemical factors that affect selective ligand recognition. The binding modalities of the two isoforms using CoMFA and MD models were compared, which suggested some key differences in the molecular interactions with the ligands and could be summarized as three subsites (one affinity subsite near the C-helix and DFG and two hydrophobic subsites). In the context of the structure–activity relationship (SAR), several new compounds were designed using a fragment-substitution strategy with the aim of selectively targeting PI3Kγ. The pIC_50_ values of the designed compounds were predicted by the 3D-QSAR models, followed by the MM-PB/GBSA binding energy estimation. The overall findings suggest that the designed compounds have the potential to be used as PI3Kγ inhibitors with a higher binding affinity and selectivity.

## 1. Introduction

Recurrent amplification and gene mutations in *PIK3CA* are closely associated with alteration in the expression level of biomarkers, such as those found in the PI3K/AKT/mTOR pathway [1,2]. Phosphoinositide 3-kinases (PI3Ks) encoded by *PIK3CA* are integral components of the AKT and mTOR cell signaling pathways and regulate divergent fundamental roles in cell proliferation, metabolism, growth, and apoptosis [3]. Overexpression and deregulation of PI3K lead to gastric mucosa in patients with advanced GC, making it the fifth most prevalent cancer type worldwide [3,4].

Despite being categorized into three different classes (class I, II, and III) based on the structure, distribution, and mechanism of action, class I PI3Ks are mostly abundant in malignancies [5]. This class was further subdivided into 1A and 1B, which are members of the lipid kinase family and exist as heterodimers of a regulatory/adapter (p85) subunit and a catalytic subunit (p110). The regulatory subunits P85 (p85α, p85β, and p55γ) and the catalytic subunits P110 (p110α, p110β, p110γ, and p110δ) differ between the PI3K isoforms [6].

**Figure 1:**
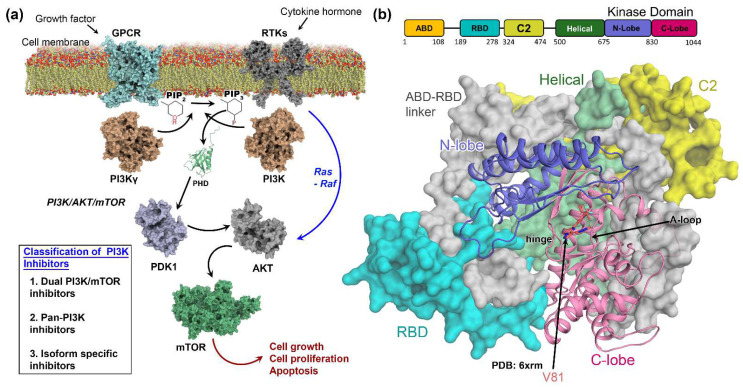
Canonical signaling and structural domain organization of PI3Kγ. (**a**) An overview of PI3Kγ-mediated AKT/mTOR signaling pathway. The activated mTOR is further involved in GC progression through the remodeling of TAM differentiation. The therapeutics available for targeting PI3K mediated pathways have been categorized into three major classes, as illustrated in the inset box. (**b**) The overall crystal structure of human PI3Kγ (PDB: 6xrm) consists of four domains: an RBD domain in cyan, a C2 domain in yellow, a helical domain in green, and a kinase domain colored in slate (N-lobe) and pink (C-lobe), respectively. The inhibitor V81 bound to the hinge is shown in orange.

As shown in Figure 1a, G-protein-coupled receptors (GPCRs) and receptor tyrosine kinases (RTKs) are auto-phosphorylated on their tyrosine residues after binding to external growth factors or cytokines, which recruit PI3K to the membrane. The recruited PI3K binds to the phosphor-tyrosine residue via one of its two Src homology-2 (SH2) domains present in the regulatory subunit, followed by subsequent allosteric activation of the catalytic subunits [7]. Class IA PI3Ks (α, β, δ isoforms) are activated by RTKs, whereas class IB (PI3Kγ) is activated by GPCRs located at the cell membrane under physiological conditions. Activated PI3K phosphorylates the substrate phosphatidylinositol 4,5 bisphosphate (PIP_2_) at the 3’-OH position, converting it to the second messenger phosphatidylinositol 3,4,5 triphosphate (PIP_3_), which binds directly to the pleckstrin homology domain (PHD) of various signaling proteins, including phosphoinositide-dependent kinase 1 (PDK1) [8]. PDK1 phosphorylates Protein Kinase B (AKT) at residue T308 in the kinase domain, leading to AKT activation. Following its activation, AKT initiates its own downstream signaling cascade involving the mammalian target of rapamycin (mTOR) [9]. Furthermore, the γ isoform of the PI3K-mediated AKT/mTOR pathway can suppress NF-κB activation while inducing CCAAT enhancer-binding protein (C/EBPβ) activation, which endorses the remodeling of the differentiation of tumor-associated macrophages (TAM) [10,11].

The involvement of PI3Kγ in GC progression is less understood, which remains a concern. However, some recent studies have suggested that PI3Kγ modulates cell proliferation and metastasis in gastric cancer by serving as an immunological checkpoint between two macrophage polarization states, i.e., an immunosuppressive M2-like state and a more inflammatory M1-like state, via remodeling of TAM differentiation [12,13]. Another study by Yuan et al. [14] demonstrated that the modified Jian-pi-yang (mJPYZ) decoction could inhibit TAM-mediated GC and metastasis by specific suppression of PI3Kγ. As a result, selective inhibition of γ isoforms is an ideal therapeutic choice. PI3K inhibitors can be classified into three major categories: 1. Dual PI3K/mTOR inhibitors, 2. Pan-PI3K inhibitors, and 3. Isoform-specific inhibitors. Dual inhibitors of PI3K and mTOR were developed after considering that their catalytic subunits share a high degree of sequence homology [14]. BEZ235 (Dactolisib), XL765, P7170, GDC-0980 (Apitolisib), SF1126, and PF-4691502 (Gedatolisib) are first-generation dual PI3K/mTOR inhibitors entering clinical trials and showed tolerable efficacy with substantial antitumor activity. Compounds such as Wortmannin, IPI-145 (Duvelisib), BKM120 (Buparlisib), GDC-0941 (Pictilisib), GDC-0032 (Taselisib) PX-886, XL147, WX-037, and LY294002 are pan-PI3K inhibitors, which were able to bind to all class I PI3K. However, pan-PI3K inhibitors have not been fully developed as anticancer drugs due to toxicity and poor pharmacokinetic concerns. In more recent studies, isoform-specific selective inhibitors have been developed, which showed greater efficacy in in vitro studies. Compounds BYL719 (Alpelisib), GDC0032, and INK1117 are the first-generation, α isoform-selective PI3K inhibitors that have entered phase I clinical trials [15,16,17,18]. IPI-549 is currently undergoing clinical trials for selective PI3Kγ in the treatment of solid tumors [19]. A potential drawback of these inhibitors is the partial blockage of AKT activation due to the presence of multiple p110 isoforms.

Figure 1b illustrates the X-ray structure of the human PI3Kγ catalytic subunit (p100γ, PDB: 6xrm) [20], which is 1044 amino acids long and structurally subdivided into five domains, i.e., an ABD domain (residue 1–108) that is not present in the crystallographic form, a RAS binding domain (RBD), a C2 domain, a helical domain, and a kinase domain. The RBD domain (residues 198–278) is adjacent to the kinase domain and is thought to be involved in the fuzzy allosteric mechanism of p110γ upon binding to the Ras protein. The C2 domain (residues 324–474) is postulated to be involved in membrane binding. The Helical domain (residues 500–675) consists of five A/B pairs of antiparallel helices similar to HEAT-repeat-containing proteins for protein–protein interactions (PPI). However, the specific mechanism of this domain remains to be elucidated. Next, the kinase domain (residues 676–1044) consists of an N-lobe and C-lobe, adjoined by a hinge loop to form the binding pocket for ATP. The residues around this pocket are homologous across different isoforms, posing a major hurdle to researchers in terms of developing ATP-competitive, γ-selective inhibitors [6,21].

Molecular modeling is an emerging technique in structure-based drug discovery that can reveal unique binding mechanisms of chemical compounds at the molecular level [21]. This study used integrated computational modeling approaches for a series of isoindoline-1-one-based PI3Kγ inhibitors, as reported in the previously published literature [20,22,23,24,25]. The analog compounds exhibited a diverse range of inhibitory activities (pIC_50_ 5.27–9.20) in the biochemical assay. Parts of the compounds in the dataset also exhibited inhibitory efficacy against the δ isoform. Thus, understanding molecular insights at the structural level of isoform-specific ligand selectivity poses an intriguing challenge. Molecular docking was performed using structurally diversified analog compounds to uncover the binding pose and protein–ligand interactions. MD and MM-PB/GBSA binding energy calculations were then employed to assess the protein–ligand stability and binding affinity. Finally, robust 3D-QSAR models based on CoMFA and CoMSIA were developed to establish the structure–activity relationship. In addition, similar 3D-QSAR models were generated by collecting the PI3Kδ inhibitors and their activity values reported in these studies [26,27,28,29,30,31,32]. Unlike 2D-QSAR, 3D-QSAR results were visually represented as colored polyhedrons to demonstrate the field contribution of the chemical descriptors, which might affect the compound’s inhibitory efficacy and selectivity of PI3Kγ over PI3Kδ. Based on the SAR scheme, several new compounds were designed using the fragment substitution strategy, and their activities were predicted by the 3D-QSAR model. Compounds with predicted pIC_50_ more than the highest active compound (pIC_50_ > 9.2) in the dataset were subjected to binding energy evaluation using the MM-PB/GBSA method.

## 2. Materials and Methods

### 2.1. Protein Structure Preparation and Molecular Docking

The structure preparation of the receptor coordinates is an essential step toward the initiation of molecular docking. The crystal structure of human PI3Kγ (PDB:6xrm) and PI3Kδ (PDB:6pyr) were retrieved from the PDB database. Since the specific objective of our study is to mechanistically understand the interactions between protein and ligand at the active site, we only remodeled the kinase domain (N term and C term) using the MODELLER webserver (University of San Francisco, San Francisco, CA, USA) using UCSF Chimera-1.14 (RBVI, UCSF, San Francisco, CA, USA). The best model was selected using the lowest DOPE score criterion and validated by analyzing the Ramachandran plot (Figure S1) on the PROCHECK webserver (PROCHECK v.3.5, DOE-MBI service, UCLA, Los Angeles, CA, USA). As described in the previous study [33], the initial ‘pdbqt’ files of proteins and ligands were prepared in the graphical version of AudoDockTools (AutoDock 4.2, Scripps Research, La Jolla, CA, USA). Polar hydrogen, Kollman charges, and AD4-type atoms were assigned to the protein during the receptor preparation. On the other hand, the ligands were assigned polar and nonpolar hydrogen and Gasteiger charges. The AutoGrid was used to generate the grid parameter file with a grid box of 50 × 40 × 40 in the X, Y, and Z dimensions. The center of the grid was set to X = −23, Y = 13, and Z = −22, with a grid spacing of 0.375. Finally, AutoDock 4.2 was executed to perform 100 docking search runs utilizing the Lamarckian Genetic Algorithm (LGA). The single protein–ligand docked complex with the lowest binding free energy was selected from the lowest positional RMSD cluster. Polar and nonpolar interactions were also taken into account for further docking pose evaluation. The RMSD of the docked pose of the crystal ligand was evaluated using the LigRMSD v1.0 [34] webserver. All selected docked complexes were taken as the initial structures for the MD simulation study.

### 2.2. Molecular Dynamics

GROMACS 2019.5 [35] was used for the MD simulation study according to previously conducted research [36]. The Amber14SB 4force field was used to prepare the topology and parameter files for the protein. The ligands were parameterized using ACPYPE [37] (or AnteChamber Python Parser interface). The protein–ligand complex was placed in a cubic periodic box and solvated using a TIP3P water model with the minimum thickness of the water wall set at 10 Å from the protein atoms. Adequate amounts of Na^+^ and Cl^-^ counter ions were added to neutralize the system and bring the NaCl concentration to 0.15 mM. The steepest descent algorithm was used for the energy minimization of the system by setting the F_max_ at 1000.0 kJ mol^−1^ nm^−1^, which eliminated torsional strain and steric clashes. The NVT and NPT ensembles were then performed for 250 ps and 500 ps, respectively, by applying positional restraining to the protein backbone and heavy atoms of the ligands with a Berendsen thermostat (V-rescale) and modified Berendsen barostat. NVT and NPT equilibration gradually heats the system to 300K and attains the pressure of 1 bar. The production of the MD run was carried out for 100 ns using the leap-frog integrator after removing the positional restraint. The long-range electrostatic interaction was estimated using the Particle mesh Ewald (PME) scheme. The cutoff distances for van der Waals and Coulombic interactions were set at 12.0 Å. The H-bonds were constrained by the LINCS algorithm, while the minimum time step was set to 2.0 fs. The above protocol was followed in all complexes. RMSDs were calculated using the built-in *‘gmx rms’* function in gromacs.

### 2.3. Binding Free Energy Estimation

The last 5 ns (500 snapshots) of each protein–ligand complex was extracted to compute the end-state binding free energy using the GMX_MMPBSA [38] package, which utilizes the MMPBSA.py module [39]. The binding free energy (${\Delta G}_{\mathrm{bind}}$) from the MM-GBSA estimation is decomposed by Equation (1)

(1) $${\Delta G}_{\mathrm{bind}}={\;\Delta G}_{\mathrm{complex}}-{\;\Delta G}_{\mathrm{protein}}-{\;\Delta G}_{\mathrm{ligand}}\;$$

(2) $${\Delta G}_{\mathrm{bind}}={\Delta E}_{\mathrm{gas}}+{\Delta G}_{\mathrm{sol}}-\;T\Delta S$$

(3) $${\Delta E}_{\mathrm{gas}}=\;\left({\Delta E}_{\mathrm{vdW}}+{\;\Delta E}_{\mathrm{ele}}\right)$$

(4) $${\Delta G}_{\mathrm{sol}}={\Delta G}_{\mathrm{GB}}+{\Delta G}_{\mathrm{SA}}$$

where the total binding free energy of the protein–ligand complex is represented by ${\Delta G}_{\mathrm{complex}}$. ${\Delta G}_{\mathrm{protein}}$ and ${\Delta G}_{\mathrm{ligand}}$ represent the total free energy of the protein and ligand separately. In Equation (2), ${\Delta E}_{\mathrm{gas}}$, and ${\Delta G}_{\mathrm{sol}}\;$ express the interaction energy between protein and ligand in the gas phase and exposed to solvent conditions. Further, ${\Delta E}_{\mathrm{gas}}$ and ${\Delta G}_{\mathrm{sol}}$ can be derived from Equations (3) and (4). ${\Delta E}_{\mathrm{vdW}}$ and ${\Delta E}_{\mathrm{ele}}$ stand for van der Waals and electrostatic energy, whereas ${\Delta G}_{\mathrm{GB}}$ and ${\Delta G}_{\mathrm{SA}}$ express the polar and nonpolar solvation free energy in the generalized Born (GB) implicit solvent. The contribution of the entropy term in the system is represented by TΔS. The entropy calculation through the nmode or quasi-harmonic (QH) approximation is computationally expensive and time-dependent. Instead, the interaction entropy (IE) method proposed by Duan et al. [40] was used to compute the TΔS term.

### 2.4. Molecular Alignment and Dataset Building

The last 1 ns average MD structure of C34 was taken from the PI3Kγ-C34 trajectory as a representative template molecule of the dataset. The remaining 214 compounds were sketched, minimized with the tripos force field with the convergence force of 0.05 kcal mol^−1^ using a maximum iteration of 2000 steps, and partial charges were applied in SYBYL-X2.1 (Tripos, Inc., St. Louis, MO, USA) as reported in earlier works [41,42]. The ‘distill rigid’ and ‘database alignment’ features were used to align the compounds with the template molecule. The dataset was developed by taking 215 molecules and their respective inhibitory activity against PI3Kγ taken together. The inhibitory activity of the compounds was converted to the negative logarithm of IC_50_ (pIC_50_) values. The dataset compounds were then divided into training sets and test sets for internal and external validation of the 3D-QSAR models. Similarly, the final 1 ns average MD structure of idelalisib was extracted from the PI3Kδ-idelalisib trajectory and considered as a template molecule. Based on the template molecule, the 213 compounds were modeled for the PI3Kδ dataset using the same protocol described above.

### 2.5. CoMFA and CoMSIA Model Building

CoMFA and CoMSIA are two 3D-QSAR methods frequently used to determine the correlation between biological activity and physicochemical properties of chemical compounds. In CoMFA, the compounds were placed one after another in a three-dimensional spatial grid box with a grid spacing of 2.0 Å by applying an energy tolerance of 30 kcal/mol. The steric descriptors (S) were calculated using Lennard-Jones potential, whereas the electrostatic descriptors (E) were calculated using the Coulombic potential. The sp^3^ carbon atoms with a net charge of +1.0 were assigned as probes. The van der Waals radii were set to 1.52 Å, while the remaining parameters were accepted by default in SYBYL-X2.1.

In addition to the steric (S) and electrostatic (E) fields, the hydrophobic (H), H-bond donor (D), and H-bond acceptor (A) descriptors fields were employed in CoMSIA. To distinguish between the probe atoms and the atoms of the molecules, Gaussian-type functions with attenuation factor (α) 0.3 were assigned to each grid point. The rest of the parameters were kept similar to the CoMFA. The descriptor fields (S, E, H, A, and D) were used in different permutation–combination processes to obtain the best possible CoMSIA model.

### 2.6. 3D-QSAR Model Validation

The partial least squares (PLS) method was used to establish the correlation statistics between observed and predicted activity in CoMFA and CoMSIA. Leave-one-out (LOO) validation was applied to obtain the cross-validation coefficient q^2^, the optimal number of components (ONC), and standard error of prediction (SEP). Subsequently, the no-validation method was performed to obtain the non-cross-validated correlation coefficient (r^2^), Fischer’s statistics (F value), and the Standard Error of Estimation (SEE). Finally, the activity values were predicted for each compound from the CoMFA and CoMSIA models.

The internal validation of the QSAR model was validated using χ^2^, RMSE, and MAE calculations. The external validation of the QSAR model was conducted using different statistical matrix parameters, such as k, k’, |r_0_^2^ − r’_0_^2^|, (r^2^ − r_0_^2^)/r^2^, r_m_^2^, $\bar{r_{m}^{\;2}}$, Δr_m_^2^, $r_{\mathrm{pred}}^{2}$, $Q_{F1}^{2}$^,^ $Q_{F2}^{2}$^,^ $Q_{F3}^{2}$, and $Q_{\mathrm{ccc}}^{2}$, as proposed by Roy et al. [43], Gramatica et al. [44], and Todeschini et al. [45]. The acceptable statistical range was given in the ‘threshold values’ column in the statistical tables, according to our previous study [46]. Additionally, the progressive scrambling Q^2^ was determined to assess the sensitivity and robustness of the selected models.

### 2.7. Applicability Domain Analysis

Since the QSAR model was developed by taking a limited number of chemical compounds, the model is incapable of covering the entire chemical space. The applicability domain includes a certain chemical space in which it can predict the activity of unknown compounds with high accuracy. In the present study, AD was analyzed by the distance-based Williams plot. In this method, AD was represented by a square area between the ±3 standardized residual and warning leverage (h*). Compounds having a leverage value (h_i_) more than warning leverage are regarded as outliers and influence the model’s fitness. The methodological details about calculating the standardized residual leverage value (h_i_) and warning leverage (h*) were performed according to the previously described studies [46,47].

### 2.8. Contour Map Analysis and SAR Study

The contour maps were generated from CoMFA and CoMSIA models in differently colored polyhedrons to illustrate the structure–activity relationship of chemical compounds according to previous studies [48,49]. The MD average structure of C34 was taken as a representative compound, and the contour maps were drawn as 3D StDev*Coeff around the molecule to elucidate the field effect of the descriptors.

### 2.9. Designing of the New Compounds and Binding Affinity Calculation

Based on the SAR analysis, we designed one hundred new compounds by fragment replacement, and their inhibitor activity was predicted by the CoMFA model of the PI3Kγ. Compounds with predicted pIC_50_ values more than 9.20 were selected, and their synthetic accessibility (SA) score and binding affinity were measured. The SA scores were assessed using SwissADME [50] server, and the binding affinity was determined by the MM-PB/GBSA method.

## 3. Results

### 3.1. Molecular Docking Analysis

**Figure 2:**
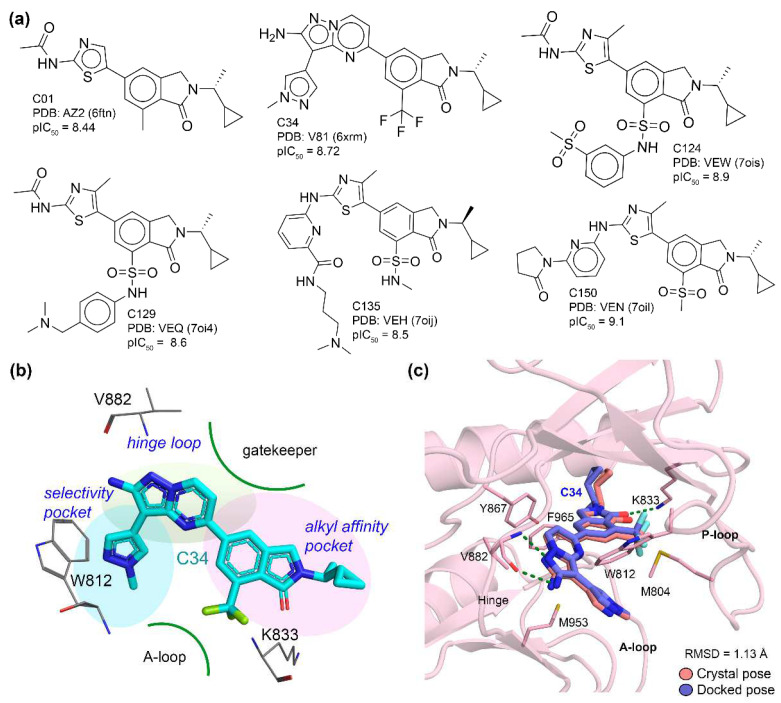
(**a**) 2D structure of the PI3Kγ inhibitors that are available in their biological form in the RCSB PDB database and present in our dataset. The compound’s number, their X-ray crystallographic code, and inhibitory activity against PI3Kγ are shown. Compounds C01 (PDB: 6ftn), C124 (PDB: 7ois), C129 (PDB: 7oij), and C150 (PDB: 7oil) were bound with mouse PI3Kδ, whereas C34 (PDB: 6xrm) was bound with human PI3Kγ. (**b**) Structural characteristics of the γ-selective PI3K inhibitors. The compounds were designed to interact with the hinge region, the alkyl affinity pocket, and the selectivity pocket. (**c**) Self-docking of compound C34 with PI3Kγ inside the ATP binding pocket. The docking process was able to reconstruct the crystal pose of C34 with an RMSD of 1.13 Å in our study. The H-bonds with residues K833 and V882 are shown by dotted green lines.

As the primary structure of the MD simulation and further QSAR model development relies on molecular docking, the docking pose verification is a crucial step to consider. A total of 18 compounds were selected from 215 compounds after manual inspection of their structural diversity in their chemical subgroups, as well as their bioactivity values. Among the selected compounds, C34 was already available in a crystallographic form bound to human PI3Kγ (PDB: 6xrm). Compounds C01, C124, C129, C135, and C150, on the other hand, were available with the mouse PI3Kδ isoform in the PDB database (PDB ID: 6ftn, 7ois, 7oi4, 7oij, and 7oil, respectively), as shown in Figure 2a. The compounds were rationally designed to interact with the residues in three different subsites: the hinge, the alkyl affinity pocket, and the selectivity pocket (Figure 2b). The experimental binding orientation was reconstructed by the self-docking of C34 to PI3Kγ with a docking score of −11.2 kcal/mol. The remaining selected compounds were cross-docked with PI3Kγ. A summary of the docking results is shown in Table 1.

However, the best docking score in cross-docking experiments is not always reliable for ranking the ligand pose. In addition to the docking score, the best docking pose was selected depending on the RMSD of the experimental ligand pose, which also complied with the Essential Chemical Interaction Described for Analog Ligands (ECIDALs) criterion. According to this research [51], the RMSD between 2.0 and 3.0 Å from the crystal pose is an acceptable solution when selecting the final ligand structure. Compound C34 interacts with the hinge loop by bidentate H-bond interactions of its aminopyrazollopyrimidine moiety with the -C=O and -NH_2_ groups of C694. The third H-bond interaction was formed between the -C=O group of C34 and the -NH_2_ group of catalytic lysine K644. Residues M804 and M953 formed π–sigma interactions with the pyrazole ring in the selectivity pocket (Figure 2c). A similar π–sigma interaction was observed between the gatekeeper residue I879 and the isoindolinone ring of C34. The residue Y867 formed the π–π edge-to-face interaction with the azaindole ring. The ethylcyclopropane moiety, on the other hand, formed hydrophobic interactions with L838, M842, L845, C869, and F965, inside the alkyl affinity pocket. The C34 self-docking effectively reproduced the intermolecular interactions identical to the original crystallographic form, suggesting overall docking reliability. The other selected compounds shared isoindolin-1-one as a common substructure with C34 and showed a similar mode of binding interaction within the ATP pockets in cross-docking according to the ECIDAL norms. The 2D docking interaction results are shown in Figure S2. Compounds C01, C118, C124, C129, and C150 had a thiazole ring as an HBM motif instead of the bicyclic azaindole ring, which created an additional π–sulfur interaction with W812. The RMSD and molecular interaction analyses strongly supported the validity of the overall docking process. The single docked conformation selected from each complex was used for the MD study.

### 3.2. MD Simulation and Protein–Ligand Stability

Given that ligand binding to its receptor is a highly dynamic process, the single protein–ligand docking conformation remains uncertain for evaluating the final binding conformation. Additionally, in the docking experiment, the receptor was treated as a rigid molecule, and the scoring function uses many approximations. Therefore, the all-atom MD simulations were used for a more rigorous conformational search and complex stability supplemented with molecular docking study. We performed 100 ns of MD simulation of each docked complex in explicit solvent conditions to assess the overall stability of each system (Figure S3). The protein–ligand complexes converged well within 10 ns of the initial MD run. The ligand RMSDs were found to be within the range of 1.0–3.0 Å. The backbone RMSDs of the proteins were found to be within 2.0–4.5 Å, which is comparable to our previous study [52]. The last 1 ns average MD structure is shown in Figure S4. The protein–ligand complexes oscillated at an RMSD of less than 5.0 Å, except for the PI3Kγ-C22 complex. Compound C22 showed a high RMSD compared to other compounds, resembling the binding instability at the active site. In the docking analysis, pyrrolidine-2-carboxamide initiated an H-bond interaction with A885. However, in the MD analysis, the pyrrolidine ring showed the steric hindrance effect with residues T886 and T887 of the selectivity pocket, which might have caused rather high RMSD values in MD simulation.

### 3.3. Free Energy Calculation

To understand the binding affinity between the receptor and its diverse set of ligands, we estimated the MM-PB/GBSA binding free energy by taking the last 500 frames from the MD trajectory of each complex. During the calculations, the dielectric constant (ε_in_) was set to 5. The in-depth binding energy terms are shown in Table 2. The entropy term (TΔS) was calculated by averaging the IE term from the last 126 snapshots, which was further subtracted from the ΔTOTAL term to obtain the final binding energy (ΔG_bind_). In the MD study, C22, which showed a higher RMSD, was estimated to have the lowest affinity towards PI3Kγ (−21.73 kcal/mol) in MM-PB/GBSA binding energy calculation. The entropy term contributed a large numerical value of 23.88 kcal/mol to the final binding energy. Residues A885, T886, and T887 formed a solvent-unexposed hydrophobic core inside the internal cavity. When the extended pyrrolidine-2-carboxamide moiety of the HBM interacted with the hydrophobically buried residues, it might be entropically detrimental to the protein–ligand binding. In terms of binding affinity, compound C150 was calculated to have the highest binding free energy of −57.35 kcal/mol. Compounds C01, C34, C41, C62, C72, C79, C81, C103, C124, and C195 exhibited binding energies of −48.40 kcal/mol, −43.21 kcal/mol, −48.42 kcal/mol, −46.03 kcal/mol, −44.69 kcal/mol, −53.31 kcal/mol, −47.33 kcal/mol, −47.44 kcal/mol, −41.58 kcal/mol, and −41.90 kcal/mol, respectively. In contrast, compounds C60, C99, C118, C129, C182, and C215 were calculated to have a slightly lower binding energy of −34.17 kcal/mol, −37.61 kcal/mol, −39.75 kcal/mol, −35.50 kcal/mol, −37.70 kcal/mol, and −37.68 kcal/mol, respectively.

**Figure 3:**
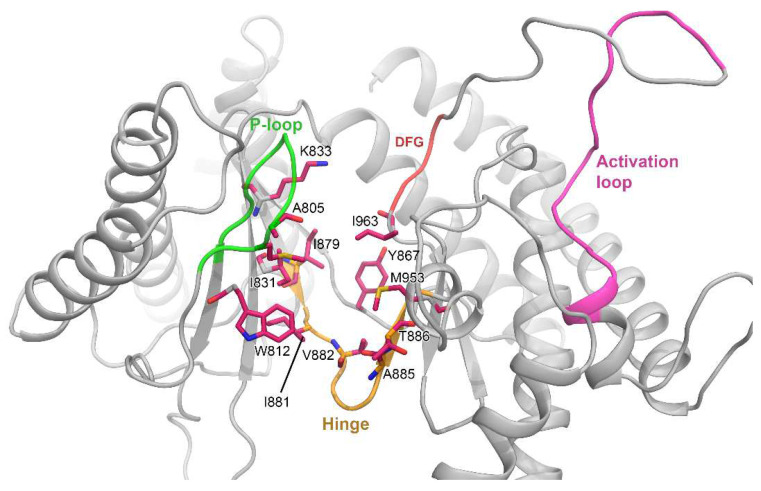
Per-residue MM-PB/GBSA binding free energy decomposition analysis. Common residues within the 4 Å of ligand atoms that contributed the binding energy to final ΔTOTAL terms are shown in stick representation. The different subdomains, such as the P-loop, Hinge, DFG motif, and activation loop, are colored in green, yellow, red, and pink.

Next, we calculated the per-residue binding energy decomposition from the residues that were within the 4 Å distances of the ligand atoms in Table 3. Additionally, residues that contributed very less or negligible binding energy to the ligand were further excluded. As expected, residues M804, W812, I831, K833, Y867, I879, I881, V882, M953, and I963 were the common BE contributing residues inside the ATP pocket. In addition, compounds C22, C41, and C60 had steric chemical extensions attached to their HBM towards the selectivity pocket; thereby, residues T886 and A885 decomposed the additional binding free energy with these compounds. Figure 3 shows our speculated generalized BE-contributing residues surrounding the active site in addition to the different subdomains.

**Figure 4:**
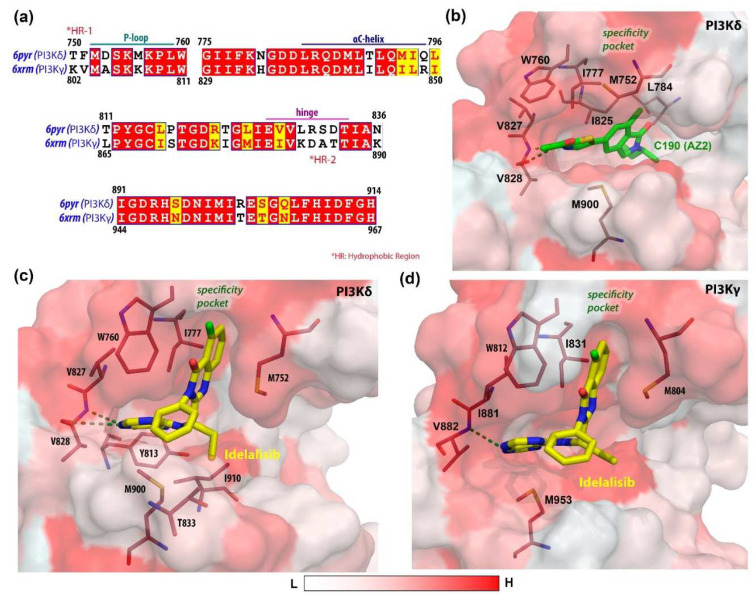
Sequence alignment of the active sites and ligand interactions inside the hydrophobic cavity of δ and γ isoforms of PI3K. (**a**) Consensus sequence alignment of the active sites of two isoforms. The conserved amino acid residues are highlighted in warm red. (**b**) The final 1 ns average MD structure of C170 (AZ2)-bound PI3Kδ. The final 1 ns average MD structures of the idelalisib-bound active sites of PI3K and PI3K, respectively, are shown in (**c**) and (**d**). The H-bond interactions are shown in dotted green lines. The white to red surface indicates ascending hydrophobicity.

We further investigated the MD study of CZ2 (C170) and idelalisib in the complex with both PI3Ks (Figure S5) to estimate the final BE, as shown in Table 4. C01 and C170 in Tables S1 and S12 are the same compounds, i.e., CZ2, which was developed as a dual receptor targeting inhibitor. This compound exhibited binding energies of −41.19 kcal/mol and −48.40 kcal/mol, respectively, to PI3Kδ and PI3Kγ. Idelalisib, on the other hand, was developed to increase δ selectivity over γ by more than 100 times [53], exhibiting binding energies of −32.48 kcal/mol and −16.48 kcal/mol, respectively in complex with PI3Kδ and PI3Kγ. However, a higher entropic energy contribution (TΔS = 12.47 kcal/mol) was observed in the PI3Kγ-idelalisib complex. The per-residue energy decomposition analysis of the individual complexes is summarized in Table 5, which shows the distinctive residue spectrum interacting with the ligand of the two isoforms. To explore the critical structural insights, the final 1 ns average MD structures of the CZ2 and idelalisib-bound isoforms were retrieved from the simulation trajectories, as shown in Figure 4. In sequence alignment, the difference in the major amino acid residues was detected in the P-loop (HR-1) and the hinge region (HR-2) as key determinant sub-sites shown in Figure 4a. In the P-loop of PI3Kδ, residues T750 and F751 are substituted with less-hydrophobic and positively charged K802 and V803 in PI3Kγ, which initially formed the entry point of the ligands, whereas, in the hinge region, S831 and D832 are replaced by more hydrophobic A885 and T886 in the γ isoform. The binding interaction of C170 with PI3Kδ appeared to be similar to PI3Kγ and was mainly stabilized by forming two H-bond interactions with V828 in the hinge loop, as shown in Figure 4b. Figure 4c,d illustrates the idelalisib-bound active sites of the two isoforms, where the ligand was stabilized by establishing at least one H-bond interaction with valine. In the specificity pocket, hydrophobic interactions were found between the bicyclic quinazoline ring of idelalisib and residues tryptophan and methionine.

### 3.4. Statistical Results from CoMFA and CoMSIA

**Figure 5:**
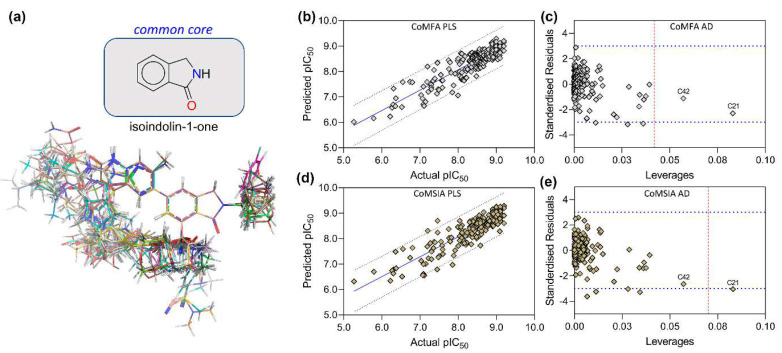
Molecular alignment and CoMFA-CoMSIA statistical plots. (**a**) The dataset compounds are aligned to the common skeleton isoindolin-1-one. (**b**) and (**c**) show the partial least squares (PLS) correlation plot and applicability domain (AD) analysis (Williams plot) of the CoMFA model by taking every compound in the dataset. (**d**,**e**) show the PLS plot and AD analysis of the CoMSIA model. The warning leverage (h*) for CoMFA (h* = 0.042) and CoMSIA (h* = 0.070) is shown in dotted red lines in the Williams plot.

From the literature, a series of 215 isoindolin-1-one-based compounds reported to be selective inhibitors of PI3Kγ isoform were collected for developing the 3D-QSAR model. The inhibitory activity data (IC_50_) were converted to the negative logarithm of activity (pIC_50_), which was further used as the dependent variable in the 3D-QSAR model. Supplementary Table S1 shows the 2D chemical structure of the compounds and their associated pIC_50_ values. The final 1 ns average structure of C34 was chosen as a template for the molecular modeling of other compounds since it was considered a bioactive 3D conformer. Compounds C02 and C106 had nonspecific activity, i.e., their activity was not determined and therefore omitted from the dataset during the model development. All 213 compounds in the dataset were aligned to the isoindolin-1-one common chemical core using the ‘database alignment’ functionality available in SYBYL X2.1. Initially, the first CoMFA and CoMSIA models were built by taking every compound from the dataset. The comprehensive statistics of both models are shown in Table 6, including the reasonable acceptance criterion. The CoMFA q^2^ and r^2^ value was predicted to be 0.612 and 0.800 at an ONC of 6, respectively. To derive the best CoMSIA model, multiple descriptor fields, namely, steric (S), electrostatic (E), hydrophobic (H), H-bond acceptor (A), and H-bond donor (D), were applied in the permutation combination process by applying Gasteiger–Marsili partial charges, as shown in Table S2. The best scores for q^2^ and r^2^ were predicted to be 0.630 and 0.784 in the combination of SEAD descriptors at an ONC of 6, respectively. The values of q^2^ and r^2^ were well above the acceptable statistical value. Following that, we predicted the pIC_50_ and residuals of each compound using both the CoMFA and CoMSIA models (Table S3). In Figure 5, the PLS plot is illustrated to correlate the actual pIC_50_ and the predicted pIC_50_. The Williams plot was used to analyze the applicability domain (AD) of the 3D-QSAR model. The leverage values of C21 were anticipated to be higher than the warning leverage (h*) in both COMFA and CoMSIA applicability domain analysis, suggesting that C21’s activity value had a significant influence on the PLS slopes of CoMFA and CoMSIA. In addition, we also determined several other statistical terms, such as χ^2^, RMSE, MAE and k, k′, |r_0_^2^ − r′_0_^2^|, $\frac{r^{2}-r_{0}^{\prime 2}}{r^{2}}$, and the ${r^{\prime }}_{m}^{2\;}$ matric, which also satisfies the proposed parameters from several studies given under the ‘threshold values’ column.

However, any QSAR model is insufficient without being validated externally by the test set compounds that were not used during model development. The dataset was divided into training and test sets for model building and external validation. Initially, the compounds were classified into three different (high, medium, and low), mutually exclusive, and nonoverlapping strata based on their pIC_50_ values. Each stratum was shuffled to distribute the compounds by random number generation and split into four sets. Following that, one set from each stratum was selected one at a time as a test from three different strata to make the final training vs. test set ratio ~3:1. This process was repeated four times so that each compound had an equal chance to participate in the test set. In this way, we obtained four different training sets and test set combinations to generate four CoMFA and CoMSIA models (Figure S6). We used different combinations of descriptor fields (S, E, H, A, and D) fields to obtain the best possible CoMSIA model for each set, as shown in Tables S4–S7. The observed pIC_50_ and the predicted pIC_50_ of each model are tabulated in Supplementary Tables S8–S11.

The statistical analysis of the CoMFA models is summarized in Table 7. For validation, we strictly followed the acceptable statistical ranges as given in the ‘threshold values’ column. The Gasteiger–Marsilli partial charges were applied and kept uniform for each model during model generation. SET-A produces q^2^, r^2^, and BS-r^2^ of 0.655, 0.854, and 0.894, respectively, at the ONC of 6, which are statistically significant. The χ^2^ and RMSE values were found to be 0.227 and 0.289, respectively. Other statistical parameters, such as k and k′, were found to be 0.999 and 0.998, and the value of r_0_^2^ and r′_0_^2^ is close to the actual r^2^. The overall results indicated good internal statistical validation. The $r_{m}^{2\;}$*_Test_* or ${r^{\prime }}_{m}^{2\;}$*_Test_* was found to be >0.5 and $r_{\mathrm{pred}}^{2}$ was found to be 0.635 in external test set validation, both of which fell within the set parameters. In SET-B, the external predictivity $r_{\mathrm{pred}}^{2}$ was found to be less than 0.6, despite having a good internally validated training set model. In SET-C, both the internal and external validation parameters were not satisfied by the acceptance criterion. Although SET-D had good external predictive power ($r_{\mathrm{pred}}^{2}=0.694$), it showed poor internal model quality. This led us to select SET-A as a final representative of the CoMFA model.

The statistical result of the CoMSIA scheme is summarized in Table 8. With the highest q^2^ and r^2^, the three best models were chosen from each set for additional internal and external validation. The SED, SEAD, and SEHAD combinations yielded the highest q^2^ and r^2^ values and good internal validation in SET-A. The SD, SED, and SEAD combinations were found to produce the best q^2^ and r^2^ CoMSIA schemes in SET-B and SET-C. In SET-D, SD, SEAD, and SHAD yielded the models with the top three best q^2^ and r^2^ values. However, the final model was adopted based on good internal and external validation parameters. Although having a good external predictive power ($r_{\mathrm{pred}}^{2}$), the models from SET-D were not selected due to the lower q^2^ and r^2^ values during the internal validation.

Overall, in SET-A, the three subsets yielded satisfactory q^2^ and r^2^ internally with statistically significant models during the external validation. SEAD had the highest internal q^2^ and r^2^ values of 0.655 and 0.804 at the ONC of 5. However, SEHAD had a better $r_{\mathrm{pred}}^{2}$ over SEAD combination; thus, the SEHAD from the SET-A was selected as the final model for describing the SAR analysis in the CoMSIA scheme.

Continuing that, we implemented progressive scrambling in the CoMFA and CoMSIA models to test their sensitivity and robustness, as shown in Table 9. This also helps to verify the optimal number of components for each model. For component 6, the SET-A in CoMFA produced the highest scrambling Q^2^ and the lowest cSDEP scores of 0.433 and 0.581. In contrast, in component number 5, SET-A produced the highest scrambling Q^2^ and cSDEP scores of 0.471 and 0.702, respectively, in CoMSIA. In both cases, the d*q*^2^/d*r*^2^*_yy’_* score was lower than the limit of 1.2. Overall, the evaluations suggested that the selected models were significant and statistically reliable.

Similar to the PI3Kγ 3D-QSAR model, we also initiated the development of the PI3Kδ 3D-QSAR model by using 213 compounds and their pIC_50_ values, as shown in Table S12. The compounds were divided into the training set and test set by stratified random number sampling (Figure S7) as previously described, obtaining the final training vs. test ratio to ~3:1. SET-A, B, and C produced unsatisfactory results during CoMFA and CoMSIA model development and henceforth were not investigated further. The training set from SET-D produced the statistically significant CoMFA model, in which the internal q^2^ and r^2^ were predicted to be 0.547 and 0.699 at an ONC of 6 (Table 10). This model showed an external predictive correlation coefficient, $r_{\mathrm{pred}}^{2}$ of 0.615. The combination of steric and H-bond acceptors produced the best PI3Kδ CoMSIA model, in which q^2^ and r^2^ were found to be 0.537 and 0.680 at an ONC of 5. In this model, the external predictivity $r_{\mathrm{pred}}^{2}$ was measured to be 0.562. We also determined other statistical criteria, such as the $r_{m}^{2\;}$ matric and $Q_{\mathrm{Fn}}^{2}\;$(n = 1, 2, 3), to determine whether the models had sufficient predictive power. The parameter’s values fell within the acceptable threshold value, thus indicating the overall reliability of the developed models. Subsequently, the progressive scrambling stability test was performed with the CoMFA and CoMSIA models (Table 11); at components 6 and 5, the highest progressive scrambling *Q*^2^ values were obtained, 0.475 and 0.471 for CoMFA and CoMSIA, respectively, with the lowest cSDEP scores. The observations above suggested that the models were stable and not based on chance correlation.

### 3.5. PLS Plots and Applicability Domain Analysis

**Figure 6:**
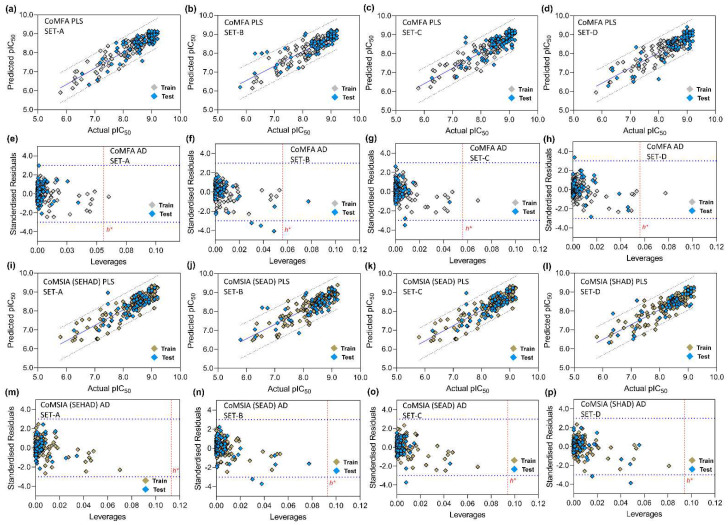
PLS correlation plot and Williams plot analysis of CoMFA and CoMSIA models. (**a**–**d**) are the PLS plots, and (**e**–**h**) are the corresponding Williams plots of the different test set groups (SET-A, SET-B, SET-C, and SET-D), respectively, in the CoMFA scheme. Similarly, (**i**–**l**) are PLS plots, and (**m**–**p**) are the corresponding Williams plots for the AD analysis of different test set groups in the CoMSIA scheme. The warning leverage (h*) is shown by dotted red lines inside the AD plots. The actual vs. predicted pIC_50_ values corresponding to the training set and test set compounds are depicted in gray and cyan in the CoMFA scheme. In the CoMSIA scheme, the values of the training and test set compounds are colored in light brown and cyan, respectively.

**Figure 7:**
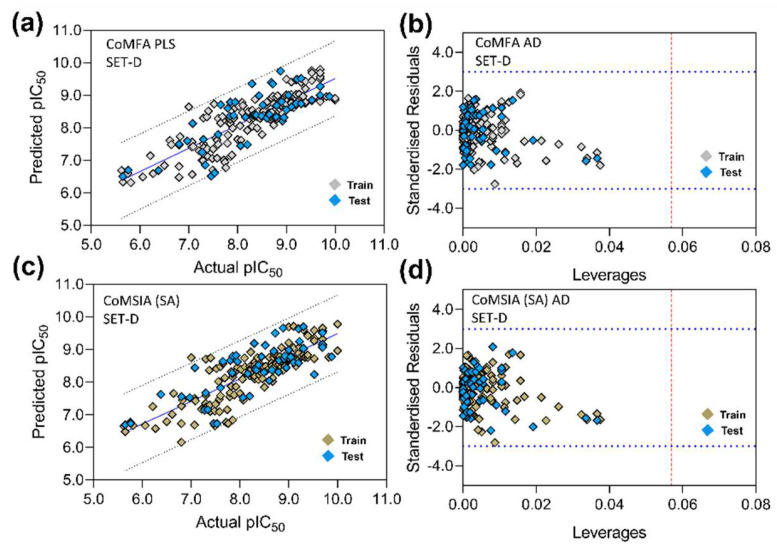
PLS correlation plot and Williams plot analysis of CoMFA and CoMSIA models of PI3Kδ. (**a**,**b**) are the PLS plot and applicability domain analysis by Williams plot of CoMFA model. (**c**,**d**) are the PLS and Williams plots of the CoMSIA (SA) model. The warning leverage (h*) for CoMFA (h* = 0.056) and CoMSIA (h* = 0.056) are shown in dotted red lines in the Williams plot.

The correlation plots (PLS) between the actual pIC_50_ and the predicted pIC_50_ of the CoMFA and CoMSIA models and the corresponding AD plots are shown in Figure 6. The PLS plots of the CoMFA models are illustrated in Figure 6a–d. The AD was analyzed using the Williams plot (Figure 6e–h) within the σ = ±3 standardized residual level and a constraint of warning leverage (h*) by a dotted red line. The leverage greater than h* signifies that the compounds strongly influence the regression slope. As observed earlier in Figure 5, the leverage value of C42 is higher than the warning leverage in each AD plot of CoMFA, where h* was 0.056. However, the value of h* was increased in the CoMSIA scheme when more descriptor fields were considered. The h* was estimated to be 0.113 in SET-A and 0.093 in SET-B, C, and D, respectively, in which all compounds fell below the permissible levels of h*. In Figure 7, the CoMFA and CoMSIA PLS plots and the Williams plots of PI3Kδ are depicted. The leverages of all compounds were within the warning h* (CoMFA h* = 0.057 and CoMSIA h* = 0.057). Overall, AD analysis suggested that the CoMFA and CoMSIA models can accurately predict the activity of an unknown compound that has a similar scaffold in a 3D chemical space.

### 3.6. Contour Maps Analysis

**Figure 8:**
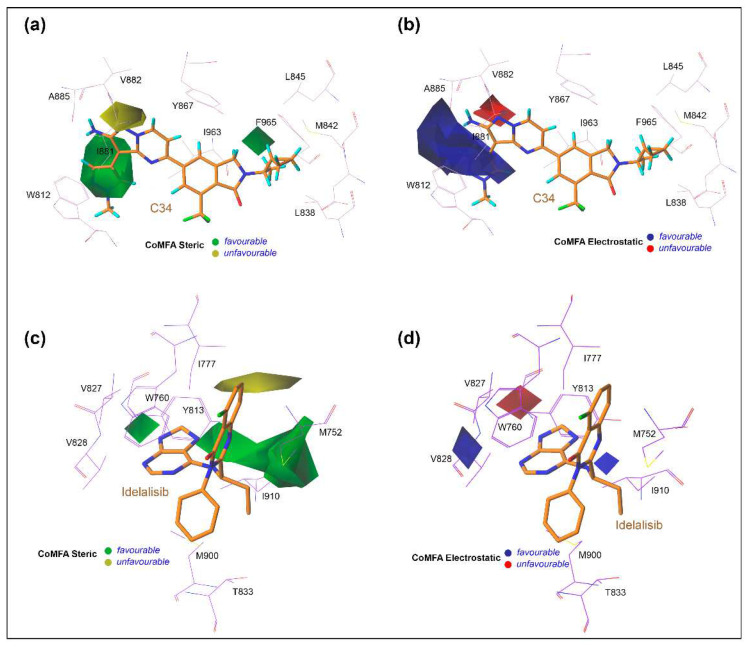
The standard deviation of coefficient contour maps of PI3Kγ and PI3Kδ CoMFA models. (**a**,**b**) contour maps depict the favorable and unfavorable substitutions for steric and electrostatic chemical groups over C34 in the PI3Kγ CoMFA scheme. (**c**,**d**) signify the favorable and unfavorable substitution for steric and electrostatic chemical groups in the PI3Kδ CoMFA scheme by taking the template molecule idelalisib as reference.

In Figure 8, the StDev*coeff of the contour maps is graphically illustrated to interpret the effects of steric and electrostatic fields of the CoMFA descriptors. The chemically meaningful different contour color scheme was used to describe the key structural features that are required for the inhibitory potency against γ and δ isoforms. The MD pose of the C34-bound active site was taken as a reference, and contour maps were generated around it for the PI3Kγ CoMFA model. In contrast, the average MD position of the idelalisib-bound active site surrounded by colored contour maps was used for the CoMFA model analysis of PI3Kδ. In the steric descriptor field, the green contours favored bulky and steric chemical entities, whereas the yellow contours did not favor that substitution. Similarly, in the electrostatic descriptor fields, blue and red contours signified the favorable position for the electropositive and electronegative groups, respectively.

In the PI3Kγ contour map (Figure 8a), a large green polyhedron appeared in the R_2_ position of the 1-methylpyrazole ring in the selectivity pocket. This suggested that a bulkier steric group at this position could increase the inhibitory activity of the compounds by interacting with residues A885 and T886. In the dataset, compounds C48-C51, C53, C55, C57-C58, C62-C74, C76-C80, C86-C104, C133-C164, C168-C195, and C200-C215 carrying bulky heterocycles in their R_2_ position showed pIC_50_ values greater than 8. Another small green contour was observed at the R_4_ position at the ethylcyclopropane moiety. A smaller steric substitution at that position could result in a stronger molecular interaction with residues D841, M842, L845, and F965, which could increase the additional inhibitory efficacy. Compounds C79, C199, C201, and C202 had a modest steric substitution and showed better inhibitory action than compounds C81 and C85. A yellow contour was seen around the HBM near the V882 residue, indicating that a steric group would be unfavorable at that location, considerably reducing the inhibitory activity of the compound. As shown in Figure 8b, a large blue contour surrounds the HBM and R_2_ position, signifying a favorable substitution of positively charged atoms or groups such as nitrogen or amine that might increase the inhibitory potency. The backbone oxygen and hydroxyl of alanine or threonine have a higher potency of H-bonding with the electropositive groups. A red contour near V882 indicated an unfavorable location for electronegative substitution and possibly hindered the formation of an H-bond in the hinge loop.

Distinctive characteristics were found in CoMFA contour maps of PI3Kδ when compared to the contours of PI3Kγ, as shown in Figure 8c,d. A large green contour appeared around the quinazoline ring near M752, Y813, and I910, and another small green contour appeared at the purine ring near the residue W760. Combining this observation indicated that a bulky steric substitution in such areas could increase the π–π stacking or π–σ interactions with the surrounding hydrophobic residues. The presence of a narrow yellow contour also indicated an undesirable site for additional steric substitution and might result in steric clashes. In Figure 8d, two small blue contours near V828 and I910 signify the favorable position for electropositive substitution, which could provide better molecular interaction and inhibitory potency to PI3Kδ. A red contour near residue W760 indicates a favorable position for an electronegative substitution, such as carboxyl (-COOH) or keto (-C=O) groups, which could provide additional interactions with surrounding residues.

**Figure 9:**
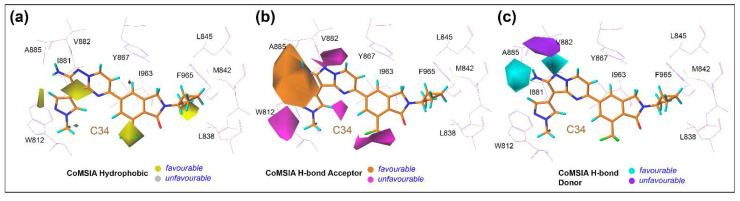
The standard deviation of coefficient contour maps of PI3Kγ CoMSIA model. In Figures (**a**–**c**), the contour maps indicate the favorable and unfavorable substitution of hydrophobic, H-bond acceptor, and H-bond donor groups over compound C34.

Additional CoMSIA contour maps of PI3Kγ and PI3Kδ are shown in Figure 9 and Figure S8. The steric and electrostatic fields were already described in the CoMFA; hence, they are not discussed further. The contour maps signifying favorable and unfavorable locations for the hydrophobic H-bond donor and H-bond acceptor are shown in multiple color schemes. The yellow contour represents the beneficial substitution for the hydrophobic chemical entities. Compounds with hydrophobic alkyl or aromatic groups in that region displayed hydrophobic interactions with the residues in the binding pocket. Additionally, in Figure 9b, the cyan and purple contours represent favorable and unfavorable substitutions for the H-bond donor atoms or chemical groups such as nitrogen, amine, and amide. Next, in Figure 9c, the magenta and orange contours represent a favorable or unfavorable substitution for H-bond donors, such as the carboxyl, keto, and sulphone groups, which could increase the inhibitory activity of the compounds. The contour polyhedrons of the H-bond acceptor of PI3Kδ (Figure S8) signify the favorable position of the H-bond acceptor moieties.

## 4. Discussion

Our study investigated the structural and mechanistic insights of ligand interactions and differences in the selectivity of the two isoforms of PI3Ks. The underlying molecular mechanism of direct involvement of PI3Kγ in GC is less studied; therefore, selective targeting could inhibit the γ-isoform-mediated remodeling of TAM differentiation. The isoindolin-1-one core-based analogs were designed to carry two independent features., i.e., interaction with the residues in the selectivity pocket and access inside the alkyl affinity pocket, while bicyclic HBM mimicked the adenine ring of ATP. The nitrogen-containing isoindolin-1-one is also a key building component of many natural products and has shown a wide range of biological activities against microorganisms, tumors, HIV, psychotic, and diabetes. Peytam et al. conducted a study [54] using isoindolin-1-one derivatives effectively against urease in the treatment of *Helicobactor pylori*-mediated gastric cancer and peptic ulcer. They synthesized sixteen compounds, two of which had inhibitory potencies 2- and 10-fold greater, respectively, than thiourea and hydroxyurea as standard inhibitors. The success of the inhibitor binding to the hinge is highly dependent on the HBM structure, which allows the improvement of the isoform selectivity by the de novo design of some hinge binding moiety. Compounds that are analogous to AZ2 have a single HBM ring system, such as the thiazole ring, which was at least able to form a single H-bond interaction with the hinge of its nitrogen atom. In compounds C134–C164 in Table S1, the substitutions of R_1_ were truncated to T886 and T887 to access the selectivity pocket. From the binding energy estimation, it could be seen that compounds with truncated subgroups (compound C150) tend to provide a higher electrostatic contribution in ΔTOTAL terms. In contrast, larger R_3_ substitutions, such as those found in C118, C124, and C129, tend to form stable H-bonding as well as hydrophobic interactions with residues K802, M804, and P810.

The structural alignment showed that both isoforms carry two non-conserved activity subsites, i.e., one is in the P-loop/HR-1, and the other is in the hinge motif or HR-2, which may lead to differences in their conformational plasticity in P-loops between the isoforms. The propeller-like-shaped idelalisib analogs were developed specifically for δ selectivity and featured additional interactions with the tryptophan shelf subsite. Analysis of the interactions between idelalisib and PI3Kδ revealed that the δ selectivity was mainly retained by interacting with the residues in P-loop, whereas the hinge binding was attributed through H-bonding with residue V828. However, the purine ring of idelalisib occupied the adenine subsite and was packed between the base and roof of the binding pocket but was not fully inserted into the polyphosphate subsite and did not interact with the residues with DFG residues. Instead, the selectivity comes from interacting with the hydrophobic tryptophan shelf via its quinazoline ring. Additional fluorine atoms in this moiety formed halogen bond interactions with F751 and T750. The F751 replaced with V803 in PI3Kγ significantly decreased the idelalisib affinity PI3Kγ interactions, according to the MD study.

One of the major universal drug design strategies for kinase inhibitors is targeting the residues in the DFG. In PI3Kγ, the alkyl affinity pocket was dominated by hydrophobic residues, such as I963, C869, L845, and F965, and residue I879 controlled the ligand insertion into the polyphosphate subsite cavity as gatekeepers. The ethylcyclopropane moiety of the C34 tail induced the interaction with the aspartate and phenylalanine residues and occupied the adjacent ribose subsite. Furthermore, this observation can be supported by the steric contour map from the CoMFA analysis. At the hinge location, although the H-bonding residue valine is common to both isoforms, the adjacent residue such as lysine to threonine (K883-T886) in HR-2 is not conserved, however, which could introduce the additional opportunity of targeting this residue for greater γ selectivity.

**Figure 10:**
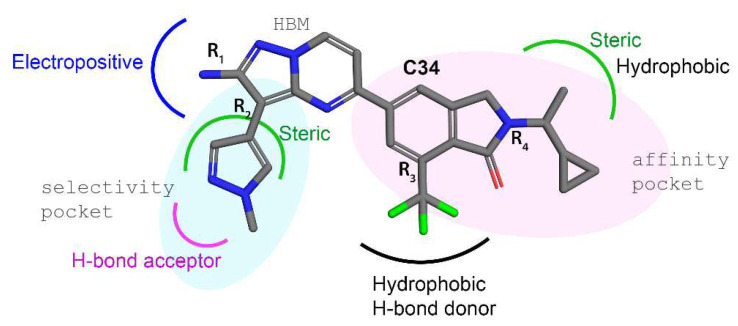
Structure–activity relationship analysis by taking C34 as a reference.

We summarize the optimum SAR scheme around C34 in Figure 10 to further optimize the efficacy of the selective PI3K inhibition from the CoMFA and CoMSIA contours. At the alkyl affinity pocket in position R_4_, substituting the steric and hydrophobic groups could increase the probability of better molecular interaction with surrounding residues, such as L845, L848, and F965 ‘alkyl-push’, as previously described in [55]. However, if the substitution is large, it could bring a steric clash. In the R_3_ position, hydrophobic and H-bond donors could be beneficial. Notably, this R_3_ position of compounds C118, C124, C129, C150, and C195 had sulphone groups that interacted with the residues in this region by forming additional sulfur–x interactions.

Additionally, from the contour analysis around the pyrazole ring in the R_2_ position, a bulky heterocyclic ring substitution that parallels or truncates parallelly to the hydrophobic core could be beneficial for better molecular interactions with A885 and T886. However, the SAR scheme around the R_1_ position of the pyrazolopyrimidine ring and R_2_ position was already investigated in earlier studies [56,57], and the interaction was found to be similar across the other isoforms. Therefore, the R_3_ and R_4_ positions may offer greater opportunities to improve selective potency and inhibitory effectiveness against the γ isoforms. The per-residue binding energy estimation combined with the CoMSIA hydrophobic contours corroborated that space-filling by attaching additional chemical groups in affinity pockets, and HR-2 could improve the binding affinity as well as selectivity of the compounds.

Based on the SAR study, we designed 100 new compounds, as shown in Supplementary Table S15, by substituting chemical fragments at positions R_2_, R_3_, X1, and X_2_ in C34. The inhibitory potencies of these compounds were assessed by the PI3Kγ CoMFA model. The pIC_50_ values of compounds D21–25, D81–85, and D87 were predicted to be higher (pIC_50_ > 9.2) than the most active compounds C113, C116, and C121 (Table S1). In contrast, the designed compounds were predicted to have low inhibitory potencies against PI3Kδ (predicted pIC_50_ < 8.0). The SA scores of the designed compounds were analyzed on the SwissADME server. The server assigns a scale of 1 to 10 scores to the compounds: an SA score of 1 indicates the ease of the synthetic route, while an SA score of 10 indicates a difficult synthetic route. The designed compounds had an SA score between 5.21 and 6.49, indicating moderate synthesis difficulty. We used the MD simulation study of the selected compounds and calculated the MM-PB/GBSA binding free energy to evaluate protein–ligand binding affinity, as shown in Figure S9 and Table 12. The BE of compounds D21–25 and D81–85, D87 were estimated to be −63.37 kcal/mol, −56.93 kcal/mol, −54.76 kcal/mol, −55.57 kcal/mol, −53.01 kcal/mol, −53.08 kcal/mol, −58.92 kcal/mol, −66.08 kcal/mol, −52.02 kcal/mol, −58.36 kcal/mol, and −50.71 kcal/mol, respectively. The ΔG_bind_ values of the designed compounds resulted in a favorable binding affinity (ΔG_bind_ > −50 kcal/mol) in the PI3Kγ-ligand interaction. When the BE of D25 in complex with PI3Kδ was calculated, a large numerical value of 16.20 kcal/mol was estimated in the TΔS term, and the final ΔG_bind_ was estimated to be −31.97 kcal/mol, indicating an unfavorable binding affinity to the δ isoform compared to the γ isoform.

## 5. Conclusions

In summary, our work aims to demonstrate key structural and mechanistic insights into the selectivity of isoform-specific inhibitors for PI3Kγ that could be a feasible therapeutic target for M2-like, macrophage-mediated GC. The molecular docking provides the binding orientation and molecular interaction in the ATP binding pocket, which were further assessed by MD simulation and BE estimation. The structure–activity relationships of δ- and γ-isoform-specific inhibitors were investigated by establishing the CoMFA and CoMSIA based 3D-QSAR models. Both models were statistically significant and had adequate prediction ability. To elucidate the selectivity and binding affinity at the structural level, the activity subsites from CoMFA and MD models of both isoforms were compared. We identified that the bulkier group substitution in the alkyl affinity pocket could increase the ligand selectivity of PI3Kγ. Following that, 100 new compounds were designed by a fragment-substitution strategy to increase the probability of molecular interaction in the binding pocket. Their inhibitory potency was predicted using PI3Kγ and PI3Kδ CoMFA models, in which the designed compounds D21–25, D81–85, and D87 showed higher pIC_50_ in the PI3Kγ CoMFA model compared to PI3Kδ. These compounds also exhibited a higher γ isoform binding affinity. The high-volume chemical moieties in X_1_ and X_2_ might favor the γ isoform binding over the other by forming additional steric interactions. The perspectives presented here may provide theoretical clues for improving the binding affinity and selectivity of PI3Kγ-targeting inhibitors through rational drug design.

## Figures and Tables

**Table 1:** Molecular docking analysis of the selected compounds with PI3Kγ.

Docked Compounds with PI3Kγ																		
	C01	C22	C34	C41	C60	C62	C72	C79	C81	C99	C103	C118	C124	C129	C150	C182	C195	C215
Docking Score (ΔG in kcal/mol)	−10.7	−11.0	−11.2	−11.4	−10.9	12.7	−13.0	−12.2	−12.2	−12.4	−13.6	−12.4	−14.4	−13.3	−14.2	−12.3	−11.8	−12.9
RMSD (Å) from crystal ligands Ref. V81 (6xrm)	2.03	2.55	0.91	2.63	2.64	2.44	2.74	1.90	1.19	2.44	1.90	2.52	2.41	1.99	2.38	1.65	1.89	1.61
Number of H-bonds	2	3	3	3	3	3	3	3	2	2	4	2	5	2	3	2	3	4
H-bond-interacting residues	V882	V882, A885	V882, K833	V882, A885	V882, A885	V882, A885	V882, K890	V882, K833	V882, K833	V882	V882, T887, K890, K833	V882	V882, K833, N951, K808	V882	V882, T887	K833, V882	K833, V882	K833, V882, K890
π–π interaction	Y867	Y867, W812	Y867	W812	Y867, W812	Y867, W812	W812	Y867, W812	W812	W812	Y867, W812	Y867	Y867, F965	Y867, F965	Y867, F965	Y867	Y867	Y867
π–Sigma bonding	I963, I879	I963	M804, M953, I879	I963	I963, M953	I881	I963	I879, M953, I963	I879, M953, I963	M953, I963	I879, M953, I963	I963	I879, I963	I879, I963	I879, M953, I963	I879	I879	I879, M953
π–sulfur interaction	M953, W812	M804, M953	-	M804, M963	M804	M953	M804, M853	-	M804	M804	-	M804, W812, M953	M804, W812, M953	M804, W812, M953	M804, W812	-	-	-

**Table 2:** Binding energy calculation of the selected compounds in complex with PI3Kγ.

Complexes	MM-PB/GBSA Binding Energy Terms in kcal/mol								
	VDW (±SD)	E_EL_ (±SD)	E_PB_/_GB_ (±SD)	E_SURF_ (±SD)	ΔG_gas_ (±SD)	ΔG_solv_ (±SD)	ΔTOTAL (±SD)	TΔS (±SD)	ΔG_bind_ (±SD)
PI3Kγ-C01	−54.40 ±2.82	−35.03 ±3.67	39.06 ±2.67	−6.63 ±0.12	−89.45 ±3.54	32.43 ±2.65	−57.01 ±2.76	8.61 ±0.20	−48.40 ±2.77
PI3Kγ-C22	−52.53 ±3.26	−23.77 ±8.87	37.55 ±6.11	−6.88 ±0.29	−76.29 ±8.82	30.67 ±6.03	−45.62 ±4.72	23.88 ±4.90	−21.73 ±6.81
PI3Kγ-C34	−60.86 ±3.22	−48.55 ±6.02	60.26 ±5.18	−7.42 ±0.21	−109.43 ±5.60	52.83 ±5.21	−56.59 ±3.68	13.38 ±0.02	−43.21 ±3.68
PI3Kγ-C41	−48.66 ±3.13	−20.19 ±5.85	26.4 3±4.05	−5.99 ±0.26	−68.86 ±5.24	20.43 ±3.96	−48.42 ±3.83	17.93 ±0.94	−48.42 ±3.83
PI3Kγ-C60	−52.67 ±2.85	−39.47 ±5.83	45.74 ±4.32	−6.64 ±0.15	−92.15 ±5.40	39.10 ±4.35	−53.05 ±3.30	18.87 ±0.04	−34.17 ±3.34
PI3Kγ-C62	−61.42 ±3.02	−34.43 ±3.54	49.40 ±2.66	−7.33 ±0.22	−95.87 ±3.98	42.07 ±2.59	−53.80 ±2.92	7.76 ±1.46	−46.03 ±3.61
PI3Kγ-C72	−69.47 ±3.48	−34.14 ±6.81	51.64 ±4.65	−7.90 ±0.24	−103.62 ±6.68	43.74 ±4.60	−59.88 ±3.70	15.19 ±3.55	−44.69 ±5.61
PI3Kγ-C79	−65.21 ±2.96	−38.13 ±4.28	48.45 ±2.83	−7.70 ±0.15	−103.36 ±4.23	40.75 ±2.81	−62.61 ±3.24	9.29 ±0.61	−53.31 ±3.30
PI3Kγ-C81	−59.05 ±3.00	−34.53 ±6.75	44.62 ±5.08	−7.04 ±0.26	−93.61 ±6.73	37.58 ±4.97	−56.03 ±3.71	8.69 ±0.04	−47.33 ±3.71
PI3Kγ-C99	−58.25 ±3.44	−56.56 ±7.52	67.51 ±6.90	−7.59 ±6.90	−114.81 ±8.76	59.91 ±6.69	−54.89 ±3.82	17.28 ±0.59	−37.61 ±3.86
PI3Kγ-C103	−61.05 ±2.90	−46.84 ±4.40	56.49 ±3.64	−7.70 ±0.21	−107.91 ±4.53	48.79 ±3.62	−59.11 ±2.95	11.67 ±2.06	−47.44 ±3.60
PI3Kγ-C118	−59.50 ±2.88	−51.98 ±5.11	65.58 ±4.12	−7.23 ±0.26	−111.49 ±4.24	58.34 ±4.07	−53.14 ±3.13	13.39 ±0.04	−39.75 ±3.13
PI3Kγ-C124	−60.09 ±3.19	−61.38 ±10.17	76.25 ±8.99	−7.12 ±0.23	−121.47 ±10.99	69.12 ±8.89	−52.34 ±3.71	10.34 ±1.59	−41.58 ±4.67
PI3Kγ-C129	−59.87 ±3.12	−36.29 ±5.69	61.99 ±5.14	−7.99 ±0.29	−96.18 ±5.95	54.01 ±5.11	−42.18 ±3.49	6.68 ±0.04	−35.50 ±3.49
PI3Kγ-C150	−72.71 ±3.14	−66.26 ±5.44	79.80 ±4.40	−8.84 ±0.20	−138.97 ±5.47	79.80 ±4.40	−68.02 ±3.56	10.66 ±0.78	−57.35 ±3.65
PI3Kγ-C182	−59.35 ±2.95	−33.72 ±6.10	47.78 ±5.09	−7.09 ±0.32	−93.07 ±5.77	40.69 ±5.14	−52.38 ±3.18	14.68 ±0.05	−37.70 ±3.18
PI3Kγ-C195	−41.90 ±3.22	−42.54 ±4.11	61.18 ±3.83	−7.94 ±0.21	−104.96 ±4.54	53.23 ±3.84	−51.73 ±3.22	9.83 ±0.03	−41.90 ±3.22
PI3Kγ-C215	−63.11 ±3.48	−64.01 ±6.16	73.01 ±4.90	−7.84 ±0.31	−127.13 ±6.60	65.16 ±4.84	−61.97 ±4.01	14.29 ±0.04	−37.68 ±4.01

VDW: van der Waals contribution from MM; E_EL_: electrostatic energy as calculated by the MM force field; E_PB/GB_: electrostatic contribution to the solvation free energy; E_SURF_: nonpolar solvation free energy; ΔG_gas_: ΔG in the gas phase; ΔG_solv_: ΔG in solvation state; ΔTOTAL: total binding free energy from MM-PB/GBSA, TΔS: entropy term; ΔG_bind_: final binding free energy.

**Table 3:** Per-residue MM-PB/GBSA binding energy decomposition in kcal/mol.

Compounds in Complex with PI3Kγ																		
Residues	C01	C22	C34	C41	C60	C62	C72	C79	C81	C99	C103	C118	C124	C129	C150	C182	C195	C215
M804	-	-	−1.04	-	-	−0.72	−3.33	−2.66	−1.56	−2.01	−3.68	-	-	−1.79	−2.16	−1.21	−1.32	−1.18
A805	-	-	-	-	-	-	−1.20	-	-		-	-	-		-	-	-	-
W812	−0.72	−0.54	−0.86	−0.71	−0.85	−1.03	−1.59	-	−0.89	−0.98	−0.87	-	-	−0.79	−1.01	−1.01	−0.92	−0.85
I831	−1.70	−1.72	−2.18	−1.61	−1.77	−1.63	−1.82	−1.83	−1.72	−1.92	-	−2.12	−1.23	−2.28	−2.15	−1.83	-	−2.06
K833	−3.10	−1.86	−1.34	−1.40	−2.62	−1.93	−1.33	−1.72	−1.55	−1.61	-	−2.52	−2.35	−2.04	−2.59	−2.14	−1.37	−1.74
Y867	−1.75	-	−2.03	−1.29	-	−1.56	−1.37	−1.69	−2.10	−1.85	-	−1.44	−1.41	-	−1.60	−2.09	−2.21	−2.05
I879	−2.98	−2.53	−2.74	−2.75	−2.98	−2.85	−2.07	−3.05	−3.01	−3.03	−2.99	−3.09	−3.03	−3.19	−3.02	−2.74	−3.20	−2.79
I881	−2.29	−0.57	−2.53	−2.21	−2.15	−2.19	−2.07	−3.02	−2.44	−2.32	−2.17	−2.21	−3.48	−1.65	−2.44	−2.56	−2.60	−2.69
V882	−3.80	-	−3.17	−2.90	−3.26	−3.22	−3.26	−3.45	−3.57	−3.27	−3.21	−3.90	−3.85	-	−3.63	−1.63	−1.55	−3.88
T886	-	−3.57	-	-	-	-	-	-	-	-	-	-	-	-	-	-	-	−0.83
A885	-	-	−0.96	−1.20	−1.41	-	-	-		-	-	-	-	-	-	-	-	-
M953	−1.06	−1.81	−1.11	−1.04	−1.57	−1.44	−1.55	−1.24	−1.65	−1.15	−1.33	−1.31	−1.69	−1.01	−2.35	−1.39	−1.43	−2.04
I963	−2.42	−2.38	−3.08	−2.18	−2.01	−2.51	−2.93	−2.51	−2.27	−2.19	−2.19	−2.68	−3.73	−3.13	−3.40	−2.77	−2.90	−2.11

(-): Residues that were more than 4Å from the compounds or contributed negligible binding energy to the ligands were kept blank.

**Table 4:** Binding energy calculation of the selected compounds with PI3K isoforms.

Complexes	MM-PB/GBSA Binding Energy Terms in kcal/mol								
	VDW (±SD)	E_EL_ (±SD)	E_GB_ (±SD)	E_SURF_ (±SD)	ΔG_gas_ (±SD)	ΔG_solv_ (±SD)	ΔTOTAL (±SD)	TΔS (±SD)	ΔG_bind_ (±SD)
PI3Kδ-C190	−49.96 ±2.96	−30.85 ±3.91	35.11 ±3.07	−6.36 ±0.17	−80.83 ±3.62	28.74 ±3.12	−52.08 ±3.35	10.89 ±0.04	−41.19 ±3.35
PI3Kδ-Idelalisib	−51.73 ±2.26	−18.71 ±3.18	36.47 ±3.21	−5.81 ±0.17	−70.45 ±3.84	30.65 ±3.20	−39.79 ±2.60	7.31 ±0.03	−32.48 ±2.60
PI3Kγ-Idelalisib	−40.74 ±3.61	−12.50 ±5.78	28.81 ±5.53	−4.51 ±0.35	−53.25 ±7.20	24.29 ±5.36	−28.95 ±3.79	12.47 ±1.86	−16.48 ±4.22

VDW: van der Waals contribution from MM; E_EL_: electrostatic energy as calculated by the MM force field; E_PB/GB_: electrostatic contribution to the solvation free energy; E_SURF_: nonpolar solvation free energy; ΔG_gas_: ΔG in the gas phase; ΔG_solv_: ΔG in solvation state; ΔTOTAL: total binding free energy from MM-PB/GBSA, TΔS: entropy term; ΔG_bind_: final binding free energy.

**Table 5:** Per-residue MM-PB/GBSA binding energy decomposition in kcal/mol.

Complexes	Residues										
	M752	W760	I777	L784	Y813	I825	V827	V828	T833	M900	I910
PI3Kδ-C190	−0.96	-	−1.65	0.87	-	−3.00	−2.44	−4.23	-	−1.05	-
PI3Kδ-Idelalisib	−2.54	−2.98	−2.54	-	−1.46	-	−3.32	−2.42	−1.26	−2.21	−1.63
PI3Kγ-Idelalisib	M804	W812	I831	I881	V882	M953					
	−2.37	−3.38	−2.15	−2.82	−1.23	−1.50					

(-): Residues that were more than 4Å from the compounds or contributed negligible binding energy to the ligands were kept blank.

**Table 6:** Statistics of the CoMFA and CoMSIA models by including all compounds from the dataset of PI3Kγ.

**3D-QSAR (All Compounds)**	Statistical Parameters															
	q^2^	ONC	SEP	r^2^	SEE	F-Value	BS-r^2^	BS-SD	χ^2^	RMSE	MAE	k	k’	|r_0_^2^-r’_0_^2^|	$\frac{r^{2}-r_{0}^{\prime 2}}{r^{2}}$	$r\prime_{m}^{2\;}$
CoMFA	0.612	6	0.460	0.800	0.330	137.507	0.854	0.025	0.391	0.324	<0.001	1.000	0.998	0.050	0.062	0.621
CoMSIA (SEAD)	0.630	6	0.448	0.784	0.344	123.686	0.833	0.024	0.446	0.338	<0.001	1.000	0.998	0.060	0.079	0.588
Threshold values	>0.5			>0.6	<<1	>100			<0.5	<0.3	≈0	0.85 ≤ k ≤ 1.15	0.85 ≤ k’ ≤ 1.15	<0.3	<0.1	>0.5

q^2^: squared cross-validated correlation coefficient; ONC: optimal number of components; SEP: standard error of prediction; r^2^: squared correlation coefficient; SEE: standard error of estimation; F-value: F-test value; BS-r^2^: bootstrapping squared correlation coefficient; χ^2^: Chi-square value; RMSE: root mean square error; MAE: mean absolute error; k: slope of the predicted vs. observed activity at zero intercepts; k’: slope of the observed vs. predicted activity at zero intercepts; r_0_^2^: squared correlation coefficient between predicted and observed activity; r’_0_^2^: squared correlation coefficient between predicted and observed activity; ${r^{\prime }}_{m}^{2\;}$: ${r^{\prime }}_{m}^{2\;}$ matrix.

**Table 7:** Statistics of the CoMFA models of training set compounds of PI3Kγ.

CoMFA (Training Set Compounds)											
Statistical Parameters	SET-A	SET-B	SET-C	SET-D	Threshold Values	Statistical Parameters	SET-A	SET-B	SET-C	SET-D	Threshold Values
q^2^	0.655	0.608	0.598	0.540	>0.5	k _Test_	1.008	0.997	1.002	1.006	0.85 ≤ k ≤ 1.15
ONC	6	6	6	5		k’ _Test_	0.995	0.999	0.994	0.990	
SEP	0.456	0.462	0.498	0.488		r^2^_Test_	0.684	0.566	0.586	0.710	
r^2^	0.854	0.842	0.831	0.762	>0.6	r_0_^2^_Test_	0.640	0.566	0.522	0.699	≈r^2^
SEE	0.296	0.294	0.323	0.351	<<1	r’_0_^2^_Test_	0.664	0.223	0.540	0.666	
F-value	148.372	134.954	124.355	97.725	>100	|r_0_^2^ − r’_0_^2^| _Test_	0.024	0.343	0.018	0.033	<0.3
BS-r^2^	0.894	0.889	0.881	0.818		$\frac{r^{2}-r_{0}^{2}}{r^{2}}$ _Test_	0.064	NA	0.109	0.015	<0.1
BS-SD	0.021	0.021	0.021	0.033		$\frac{r^{2}-r_{0}^{\prime 2}}{r^{2}}$ _Test_	0.030	0.60	0.078	0.061	
χ^2^	0.227	0.221	0.269	0.256	<1.0	$r_{m}^{2\;}$ _Test_	0.540	NA	0.437	0.635	>0.5
RMSE	0.289	0.287	0.315	0.307	<0.5	${r^{\prime }}_{m}^{2\;}$ _Test_	0.587	0.234	0.460	0.561	
MAE	<0.001	<0.001	<0.001	<0.001	≈ 0	$\bar{r_{m}^{2}}$ _Test_	0.563	0.117	0.448	0.598	>0.5
RSS	13.335	13.120	15.83	15.02		Δr_m_^2^_Test_	0.024	0.234	0.012	0.037	<0.2
k *_Train_*	0.999	1.000	1.000	1.000	0.85 ≤ k ≤ 1.15	$r_{\mathrm{pred}}^{2}$	0.635	0.565	0.522	0.694	>0.5
k’_*Train*_	0.998	0.998	0.998	0.998		$Q_{F1}^{2}$	0.635	0.565	0.522	0.694	
r_0_^2^ *_Train_*	0.854	0.841	0.830	0.810	≈r^2^	$Q_{F2}^{2}$	0.628	0.565	0.520	0.693	
r’_0_^2^ *_Train_*	0.829	0.812	0.796	0.766		$Q_{F3}^{2}$	0.635	0.565	0.520	0.694	
|r_0_^2^-r’_0_^2^|_*Train*_	0.025	0.029	0.034	0.044	<0.3	$Q_{\mathrm{ccc}}^{2}$	0.820	0.740	0.730	0.838	
$\frac{r^{2}-r_{0}^{\prime 2}}{r^{2}}$ _*Train*_	0.029	0.035	0.042	0.052	<0.1	S (%)	44.8	43.7	43.7	44.9	
${r^{\prime }}_{m}^{2\;}$ _*Train*_	0.718	0.696	0.676	0.609	>0.5	E (%)	55.2	56.3	56.3	55.1	

q^2^: squared cross-validated correlation coefficient; ONC: optimal number of components; SEP: standard error of prediction; r^2^: squared correlation coefficient; SEE: standard error of estimation; F-value: F-test value; BS-r^2^: bootstrapping squared correlation coefficient; χ^2^: Chi-square value; RMSE: root mean square error; MAE: mean absolute error; k: slope of the predicted vs. observed activity at zero intercepts; k’: slope of the observed vs. predicted activity at zero intercepts; r_0_^2^: squared correlation coefficient between predicted and observed activity; r’_0_^2^: squared correlation coefficient between predicted and observed activity; ${r^{\prime }}_{m}^{2\;}$: ${r^{\prime }}_{m}^{2\;}$ matrix; $r_{\mathrm{pred}}^{2}$: predictive correlation coefficient; S: steric; E: electrostatic.

**Table 8:** Statistics of the CoMSIA models of training set compounds of PI3Kγ.

CoMSIA (Training Set Compounds)													
Statistical Parameters	SET-A			SET-B			SET-C			SET-D			Threshold Values
	SED	SEAD	SEHAD	SD	SED	SEAD	SD	SED	SEAD	SD	SEAD	SHAD	
q^2^	0.653	0.655	0.652	0.604	0.608	0.597	0.607	0.610	0.603	0.568	0.566	0.581	>0.5
ONC	6	5	6	6	5	6	6	5	6	6	6	6	
SEP	0.457	0.454	0.457	0.465	0.461	0.469	0.492	0.489	0.494	0.475	0.476	0.468	
r^2^	0.817	0.804	0.824	0.763	0.788	0.789	0.775	0.788	0.814	0.754	0.790	0.796	>0.6
SEE	0.332	0.342	0.324	0.360	0.339	0.339	0.372	0.360	0.338	0.359	0.331	0.326	<<1
F-value	113.148	125.375	120.235	81.648	113.465	94.986	87.143	113.508	111.033	77.440	95.264	98.962	>100
BS- r^2^	0.867	0.842	0.878	0.813	0.825	0.842	0.824	0.824	0.868	0.801	0.848	0.852	
BS-SD	0.024	0.027	0.021	0.028	0.027	0.025	0.027	0.027	0.020	0.031	0.025	0.024	
χ^2^	0.303	0.325	0.289	0.359	0.331	0.326	0.390	0.370	0.317	0.363	0.297	0.292	<1.0
RMSE	0.324	0.335	0.316	0.351	0.332	0.331	0.364	0.353	0.330	0.350	0.322	0.318	<0.5
MAE	<0.001	<0.001	<0.001	<0.001	<0.001	<0.001	<0.001	<0.001	<0.001	<0.001	<0.001	<0.001	≈0
RSS	16.725	17.935	15.915	19.65	17.628	17.478	21.078	19.865	17.338	19.56	16.680	16.178	
k *_Train_*	0.999	1.000	0.999	0.999	0.999	0.999	0.999	0.999	1.000	0.999	0.999	0.999	0.85 ≤ k ≤ 1.15
k’_*Train*_	0.998	0.998	0.998	0.998	0.998	0.998	0.998	0.998	0.998	0.998	0.998	0.998	
r_0_^2^ *_Train_*	0.816	0.803	0.825	0.781	0.807	0.791	0.795	0.821	0.843	0.764	0.811	0.805	≈r^2^
r’_0_^2^ *_Train_*	0.776	0.756	0.789	0.677	0.721	0.708	0.707	0.740	0.786	0.645	0.730	0.735	
|r_0_^2^-r’_0_^2^|_*Train*_	0.04	0.020	0.036	0.104	0.085	0.083	0.087	0.080	0.057	0.119	0.081	0.069	<0.3
$\frac{r^{2}-r_{0}^{\prime 2}}{r^{2}}$ _*Train*_	0.050	0.063	0.042	0.112	0.084	0.100	0.087	0.060	0.034	0.143	0.075	0.075	<0.1
${r^{\prime }}_{m}^{2\;}$ _*Train*_	0.651	0.627	0.669	0.539	0.585	0.565	0.573	0.616	0.677	0.505	0.597	0.600	>0.5
k_*Test*_	1.000	1.001	1.000	1.003	1.004	1.000	0.991	1.004	1.001	1.003	0.999	1.000	0.85 ≤ k ≤ 1.15
k’_*Test*_	0.997	0.996	0.996	0.992	0.992	0.995	1.006	0.993	0.996	0.993	0.998	0.996	
r^2^ *_Test_*	0.613	0.631	0.634	0.539	0.541	0.559	0.572	0.582	0.610	0.716	0.729	0.695	
r_0_^2^ *_Test_*	0.592	0.609	0.609	0.533	0.537	0.556	0.524	0.555	0.580	0.717	0.729	0.695	≈r^2^
r’_0_^2^ *_Test_*	0.529	0.562	0.572	0.332	0.330	0.341	0.512	0.494	0.540	0.642	0.648	0.604	
|r_0_^2^-r’_0_^2^|_*Test*_	0.063	0.047	0.037	0.201	0.207	0.215	0.012	0.060	0.031	0.075	0.081	0.090	<0.3
$\frac{r^{2}-r_{0}^{2}}{r^{2}}$ *_Test_*	0.034	0.034	0.039	0.011	0.007	0.004	0.083	0.045	0.049	-	-	-	<0.1
$\frac{r^{2}-r_{0}^{\prime 2}}{r^{2}}$ *_Test_*	0.137	0.109	0.097	0.384	0.389	0.389	0.103	0.149	0.101	0.102	0.110	0.129	
$r_{m}^{2\;}$ _*Test*_	0.524	0.537	0.533	0.498	0.510	0.530	0.447	0.487	0.504	-	-	-	>0.5
${r^{\prime }}_{m}^{2\;}$ _*Test*_	0.435	0.465	0.476	0.293	0.292	0.298	0.432	0.410	0.458	0.522	0.522	0.486	
$\bar{r_{m}^{2}}$ _*Test*_	0.479	0.501	0.504	0.395	0.401	0.414	0.440	0.448	0.481	-	-	-	
Δr_m_^2^_*Test*_	0.089	0.072	0.057	0.204	0.217	0.231	0.014	0.077	0.046	-	-	-	<0.2
$r_{\mathrm{pred}}^{2}$	0.599	0.615	0.616	0.526	0.529	0.554	0.517	0.546	0.577	0.713	0.729	0.693	>0.5
$Q_{F1}^{2}$	0.599	0.615	0.616	0.526	0.529	0.554	0.517	0.546	0.577	0.713	0.729	0.693	
$Q_{F2}^{2}$	0.592	0.608	0.609	0.526	0.529	0.554	0.517	0.546	0.576	0.713	0.729	0.693	
$Q_{F3}^{2}$	0.599	0.615	0.616	0.526	0.529	0.554	0.517	0.546	0.577	0.713	0.729	0.693	
$Q_{\mathrm{ccc}}^{2}$	0.781	0.793	0.795	0.722	0.723	0.733	0.747	0.761	0.781	0.840	0.845	0.693	
S (%)	20.6	15.9	13.5	33.6	23.1	23.3	32.1	21.4	15.3	30.5	15.3	15.7	
E (%)	35.8	26.4	23.6	-	37.4	-	-	34.7	25.7	-	23.5	-	
H (%)	-	-	14.7	-	-	39.2	-	-		-	-	20.9	
A (%)	-	26.7	21.6	-	-	-	-	-	24.4	-	25.7	28.4	
D (%)	43.6	31.0	26.6	66.4	39.4	47.2	67.9	43.9	34.6	69.5	35.4	35.1	

q^2^: squared cross-validated correlation coefficient; ONC: optimal number of components; SEP: standard error of prediction; r^2^: squared correlation coefficient; SEE: standard error of estimation; F-value: F-test value; BS-r^2^: bootstrapping squared correlation coefficient; χ^2^: Chi-square value; RMSE: root mean square error; MAE: mean absolute error; k: slope of the predicted vs. observed activity at zero intercepts; k’: slope of the observed vs. predicted activity at zero intercepts; r_0_^2^: squared correlation coefficient between predicted and observed activity; r’_0_^2^: squared correlation coefficient between predicted and observed activity; ${r^{\prime }}_{m}^{2\;}$: ${r^{\prime }}_{m}^{2\;}$ matrix; $r_{\mathrm{pred}}^{2}$: predictive correlation coefficient; S: steric; E: electrostatic; H: hydrophobic; A: H-bond acceptor; D: H-bond donor.

**Table 9:** Progressive scrambling results from the CoMFA and CoMSIA of PI3Kγ.

Components	CoMFA			CoMSIA (SEHAD)		
	SET-A			SET-A		
	Q^2^	cSDEP	d*q*^2^/d*r*^2^*_yy’_*	Q^2^	cSDEP	d*q*^2^/d*r*^2^*_yy’_*
1	0.083	0.730	0.095	0.245	0.662	0.179
2	0.347	0.618	0.407	0.329	0.626	0.306
3	0.380	0.608	0.376	0.451	0.568	0.533
4	0.405	0.593	0.565	0.480	0.555	0.568
5	0.400	0.599	0.502	0.507	0.542	0.735
6	0.433	0.581	0.590	0.508	0.541	0.757
7	0.333	0.634	0.681	0.489	0.555	0.787
8	0.315	0.644	0.543	0.462	0.571	0.874

**Table 10:** Statistics of the CoMFA and CoMSIA models of PI3Kδ.

Statistical Parameters	CoMFA	CoMSIA (SA)	Threshold Values	Statistical Parameters	CoMFA	CoMSIA	Threshold Values
	SET-D	SET-D			SET-D	SET-D	
q^2^	0.547	0.537	>0.5	r^2^ *_Test_*	0.627	0.577	>0.5
ONC	6	5		r_0_^2^ *_Test_*	0.616	0.566	
SEP	0.653	0.658		r’_0_^2^ *_Test_*	0.501	0.349	
r^2^	0.699	0.680	>0.6	|r_0_^2^ − r’_0_^2^|_*Test*_	0.114	0.217	<0.3
SEE	0.532	0.556	<<1	$\frac{r^{2}-r_{0}^{2}}{r^{2}}$ *_Test_*	0.017	0.018	<0.1
F-value	58.178	52.427		$\frac{r^{2}-r_{0}^{\prime 2}}{r^{2}}$ *_Test_*	0.199	0.394	
BS- r^2^	0.756	0.713		$r_{m}^{2\;}$ _*Test*_	0.561	0.517	
BS-SD	0.039	0.042		${r^{\prime }}_{m}^{2\;}$ _*Test*_	0.405	0.301	
χ^2^	0.631	0.683	<1.0	$\bar{r_{m}^{2}}$ _*Test*_	0.156	0.215	
RMSE	0.482	0.495	<0.5	Δr_m_^2^_*Test*_	0.483	0.409	
MAE	<0.001	<0.001	≈0	$r_{\mathrm{pred}}^{2}$	0.615	0.562	>0.5
RSS	36.61	38.59		$Q_{F1}^{2}$	0.615	0.562	
k *_Train_*	1.001	1.001	0.85 ≤ *k* ≤ 1.15	$Q_{F2}^{2}$	0.615	0.562	
k’_*Train*_	0.994	0.995		$Q_{F3}^{2}$	0.615	0.562	
r_0_^2^ *_Train_*	0.742	0.728	≈r^2^	$Q_{\mathrm{ccc}}^{2}$	0.785	0.744	
r’_0_^2^ *_Train_*	0.633	0.615		S (%)	76.2	47.9	
|r_0_^2^ − r’_0_^2^|_*Train*_	0.109	0.112	<0.3	E (%)	23.8	-	
$\frac{r^{2}-r_{0}^{\prime 2}}{r^{2}}$ _*Train*_	0.093	0.094	<0.1	A (%)	-	52.1	
${r^{\prime }}_{m}^{2\;}$ _*Train*_	0.520	0.507	>0.5				
k_*Test*_	0.989	0.985	0.85 ≤ *k* ≤ 1.15				
k’_*Test*_	1.005	1.008					

q^2^: squared cross-validated correlation coefficient; ONC: optimal number of components; SEP: standard error of prediction; r^2^: squared correlation coefficient; SEE: standard error of estimation; F-value: F-test value; BS-r^2^: bootstrapping squared correlation coefficient; χ^2^: Chi-square value; RMSE: root mean square error; MAE: mean absolute error; k: slope of the predicted vs. observed activity at zero intercepts; k’: slope of the observed vs. predicted activity at zero intercepts; r_0_^2^: squared correlation coefficient between predicted and observed activity; r’_0_^2^: squared correlation coefficient between predicted and observed activity; ${r^{\prime }}_{m}^{2\;}$: ${r^{\prime }}_{m}^{2\;}$ matrix; $r_{\mathrm{pred}}^{2}$: predictive correlation coefficient; S: steric; E: electrostatic; A: H-bond acceptor.

**Table 11:** Progressive scrambling results from the CoMFA and CoMSIA of PI3Kδ.

Components	CoMFA			CoMSIA (SA)		
	SET-A			SET-A		
	Q^2^	cSDEP	d*q*^2^/d*r*^2^*_yy’_*	Q^2^	cSDEP	d*q*^2^/d*r*^2^*_yy’_*
1	0.380	0.750	0.229	0.388	0.746	0.160
2	0.410	0.735	0.191	0.432	0.726	0.247
3	0.434	0.722	0.257	0.457	0.707	0.271
4	0.468	0.709	0.234	0.463	0.706	0.299
5	0.467	0.702	0.239	0.471	0.702	0.380
6	0.475	0.702	0.377	0.448	0.719	0.386
7	0.458	0.716	0.372	0.441	0.727	0.426
8	0.422	0.742	0.402	0.436	0.733	0.760

**Table 12:** SA score prediction and binding energy calculation of the newly designed compounds.

Complexes	Compound’s SA Score (1–10 Scale)	MM-PB/GBSA Binding Energy Terms in kcal/mol								
		VDW (±SD)	E_EL_ (±SD)	E_GB_ (±SD)	E_SURF_ (±SD)	ΔG_gas_ (±SD)	ΔG_solv_ (±SD)	ΔTOTAL (±SD)	TΔS (±SD)	ΔG_bind_ (±SD)
PI3Kγ-D21	5.21	−74.71 ±3.49	−63.37 ±7.34	79.14 ±5.06	−9.34 ±0.28	−138.07 ±7.70	69.80 ±5.07	−68.27 ±4.51	4.89 ±0.05	−63.37 ±4.51
PI3Kγ-D22	5.24	−70.91 ±3.32	−32.79 ±4.35	43.05 ±4.38	−8.46 ±0.29	−103.68 ±5.15	34.58 ±4.31	−69.10 ±3.49	12.16 ±0.04	−56.93 ±3.49
PI3Kγ-D23	6.49	−80.03 ±3.06	−32.53 ±5.05	57.58 ±3.75	−9.84 ±0.31	−112.55 ±5.62	47.74 ±3.75	−64.81 ±4.00	10.04 ±0.04	−54.76 ±4.00
PI3Kγ-D24	6.49	−76.56 ±2.74	−30.97 ±4.42	51.69 ±3.94	−7.99 ±0.32	−107.53 ±5.00	43.70 ±3.88	−63.83 ±2.73	8.26 ±0.07	−55.57 ±2.74
PI3Kγ-D25	6.29	−74.62 ±3.43	−41.99 ±5.22	62.27 ±5.12	−9.07 ±0.23	−116.60 ±5.81	53.19 ±5.04	−63.40 ±3.79	10.38 ±0.05	−53.01 ±3.79
PI3Kγ-D81	5.90	−75.02 ±3.51	−57.42 ±4.84	74.08 ±3.66	−9.02 ±0.27	−132.43 ±4.94	65.06 ±3.69	−67.37 ±4.07	14.29 ±1.08	−53.08 ±4.21
PI3Kγ-D82	5.90	−76.94 ±3.66	−60.04 ±5.31	75.03 ±3.68	−8.49 ±0.33	−126.98 ±5.36	66.54 ±3.70	−70.43 ±3.96	11.51 ±0.04	−58.92 ±3.96
PI3Kγ-D83	6.25	−79.62 ±3.20	−49.95 ±5.07	63.36 ±4.33	−9.47 ±0.22	−129.56 ±5.40	53.89 ±4.31	−75.66 ±3.82	9.58 ±0.05	−66.08 ±3.82
PI3Kγ-D84	6.24	−71.58 ±3.28	−55.07 ±4.53	72.55 ±3.45	−9.38 ±0.30	−126.65 ±4.67	63.17 ±3.48	−63.47 ±3.48	11.45 ±1.33	−52.02 ±3.72
PI3Kγ-D85	6.16	−74.15 ±2.92	−55.01 ±4.98	73.14 ±4.18	−9.04 ±0.22	−129.15 ±4.81	64.10 ±4.19	−65.05 ±3.44	6.69 ±0.03	−58.36 ±3.44
PI3Kγ-D87	5.82	−71.01 ±3.59	−49.86 ±4.95	67.38 ±3.96	−8.83 ±0.24	−120.88 ±5.87	58.54 ±3.87	−62.33 ±3.54	11.61 ±1.81	−50.71 ±3.98
PI3Kδ-D25	6.29	−59.63 ±2.58	−37.07 ±7.05	57.23 ±5.88	−8.70 ±0.23	−96.70 ±6.53	48.52 ±5.93	−48.17 ±2.79	16.20 ±0.05	−31.97 ±2.79

SA: synthetic accessibility; VDW: van der Waals contribution from MM; E_EL_: electrostatic energy as calculated by the MM force field; E_PB/GB_: electrostatic contribution to the solvation free energy; E_SURF_: nonpolar solvation free energy; ΔG_gas_: ΔG in the gas phase; ΔG_solv_: ΔG in solvation state; ΔTOTAL: total binding free energy from MM-PB/GBSA, TΔS: entropy term; ΔG_bind_: final binding free energy.

## Data Availability

Data available within the article or its supplementary information.

## Supplementary Materials

The following supporting information can be downloaded at: https://www.mdpi.com/article/10.3390/biomedicines10040813/s1. Figure S1: Ramachandran Plot analysis of loop modeled PI3K isoforms. The residues of the PI3Kγ and PI3Kδ were within the well-accepted regions; Figure S2: Molecular Docking analysis of the compounds C01, C22, C34, C41, C60, C62, C72, C79, C81, C99, C103, C118, C124, C129, C150, C182, C195, and C215, respectively. The H-bond, hydrophobic, π-Sulphur, π-sigma, and π-π interactions were highlighted by different color schemes by the 2D diagrams; Figure S3: RMSD plots from Molecular Dynamics simulation study of the receptor-ligand complexes. The RMSDs of the ligand and α-carbon of the receptors were shown in slate and grey; Figure S4: The final 1 ns average MD pose of compounds C01, C22, C34, C41, C60, C62, C72, C79, C81, C99, C103, C118, C124, C129, C150, C182, C195, and C215, respectively, in complex with PI3Kγ. The receptor, interacting residues, and ligands were shown in light-grey, salmon, and slate respectively. The residues are selected from the per-residue MM-PB/GBSA binding energy decomposition analysis (Table 3, in the main text); Figure S5: RMSD plots from the Molecular Dynamics simulation study of (a)PI3Kδ-CZ2, (b)PI3Kδ-Idelalisib, and (c) PI3Kγ-Idelalisib complexes; Figure S6: Random sampling table to select the Test set compounds from the dataset; Figure S7: Random sampling table to select the Test set compounds from the dataset of PI3Kδ; Figure S8: The standard deviation of coefficient contour maps was obtained from the CoMSIA model of PI3Kδ. The contour maps indicate the favorable and unfavorable substitution for hydrophobic, H-bond acceptor, and H-bond donor groups over the compound idelalisib; Figure S9: RMSD plots from molecular dynamics simulation study of the receptor and designed compounds. The RMSDs of the ligand and α-carbon of the receptors were shown in slate and grey; Table S1: Structure and activity values of isoindolin-1-one based PI3Kγ inhibitors; Table S2: Brief statistical analysis to generate the best CoMSIA model using various combinations of the descriptor fields; Table S3: Actual pIC50 vs. Predicted pIC50 of the compounds from the CoMFA and CoMSIA by taking every compound from the dataset; Table S4: Statistical analysis to generate the best CoMSIA model using various combinations of the descriptors field for SET-A compounds; Table S5: Statistical analysis to generate the best CoMSIA model using various combinations of the descriptors field for SET-B compounds; Table S6: Statistical analysis to generate the best CoMSIA model using various combinations of the descriptors field for SET-C compounds; Table S7: Statistical analysis to generate the best CoMSIA model using various combinations of the descriptors field for SET-D compounds; Table S8: Actual pIC50 vs Predicted pIC50 of the compounds from the CoMFA and CoMSIA training set compounds of SET-A; Table S9: Actual pIC50 vs Predicted pIC50 of the compounds from the CoMFA and CoMSIA training set compounds of SET-B; Table S10: Actual pIC50 vs. Predicted pIC50 of the compounds from the CoMFA and CoMSIA training set compounds of SET-C; Table S11: Actual pIC50 vs. Predicted pIC50 of the compounds from the CoMFA and CoMSIA training set compounds of SET-D; Table S12. Structure and activity values of PI3Kδ inhibitors; Table S13: Statistical analysis to generate the best CoMSIA model using various combinations of the descriptors field for SET-D compounds; Table S14: Actual pIC50 vs. Predicted pIC50 of the compounds from the CoMFA and CoMSIA training set compounds of SET-D; Table S15: Newly designed compounds and their predicted pIC50 values against PI3Kγ and PI3Kδ.

## Abbreviations

PI3K	Phosphoinositol-3-kinase.
GC	Gastric Carcinoma.
TAM	Tumor-associated macrophage.
MM-PB/GBSA	Molecular Mechanics-Poison–Boltzmann/generalized Born Surface Area.
LIE	Linear Interaction Energy.
BE	Binding Energy.
CoMFA	Comparative Molecular Field Analysis.
CoMSIA	Comparative Molecular Similarity Indices Analysis.
3D-QSAR	3-Dimensional Structure–Activity Relationship.
