# Supplementary material for: Structural Insights from Molecular Modeling of Isoindolin-1-One Derivatives as PI3Kγ Inhibitors against Gastric Carcinoma
Source: Biomedicines. 2022 Mar 30;10(4):813. doi: 10.3390/biomedicines10040813 (PMC9030798; doi:10.3390/biomedicines10040813)
Supplement: Supplementary file 1 [file biomedicines-10-00813-s001.zip › biomedicines-1609317-supplementary.pdf]

Supplementary Data: Structural insights from molecular modeling of isoindolin-1-one derivatives as PI3Kγ inhibitors against gastric carcinoma

Suparna Ghosh[1], Seung Joo Cho [1,2,*]

[1]Department of Biomedical Sciences, College of Medicine, Chosun University, Gwangju 501-759, Republic of Korea; s.ghosh@chosun.kr (S.G); chosj@chosun.ac.kr (S.J.C)
[2]Department of Cellular and Molecular Medicine, College of Medicine, Chosun University, Gwangju 501-759, Republic of Korea

[*]Correspondence: chosj@chosun.ac.kr; Tel.: +82-62-230-7482 or +82-11-479-1010

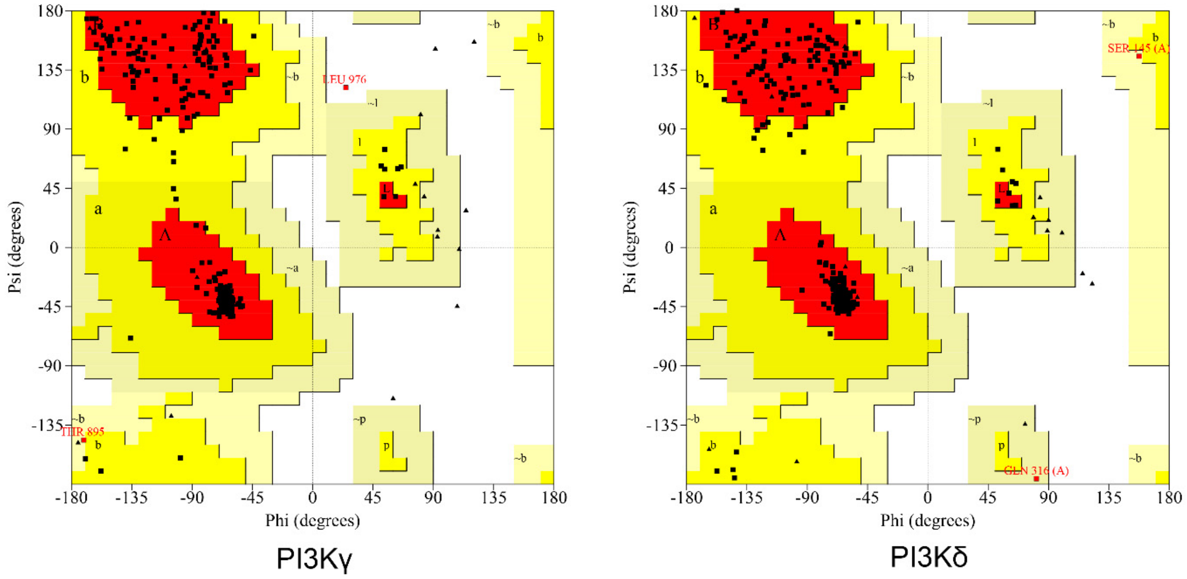

Figure S1. Ramachandran Plot analysis of loop modeled PI3K isoforms. The residues of the PI3Kγ and PI3Kδ were within the well-accepted regions.

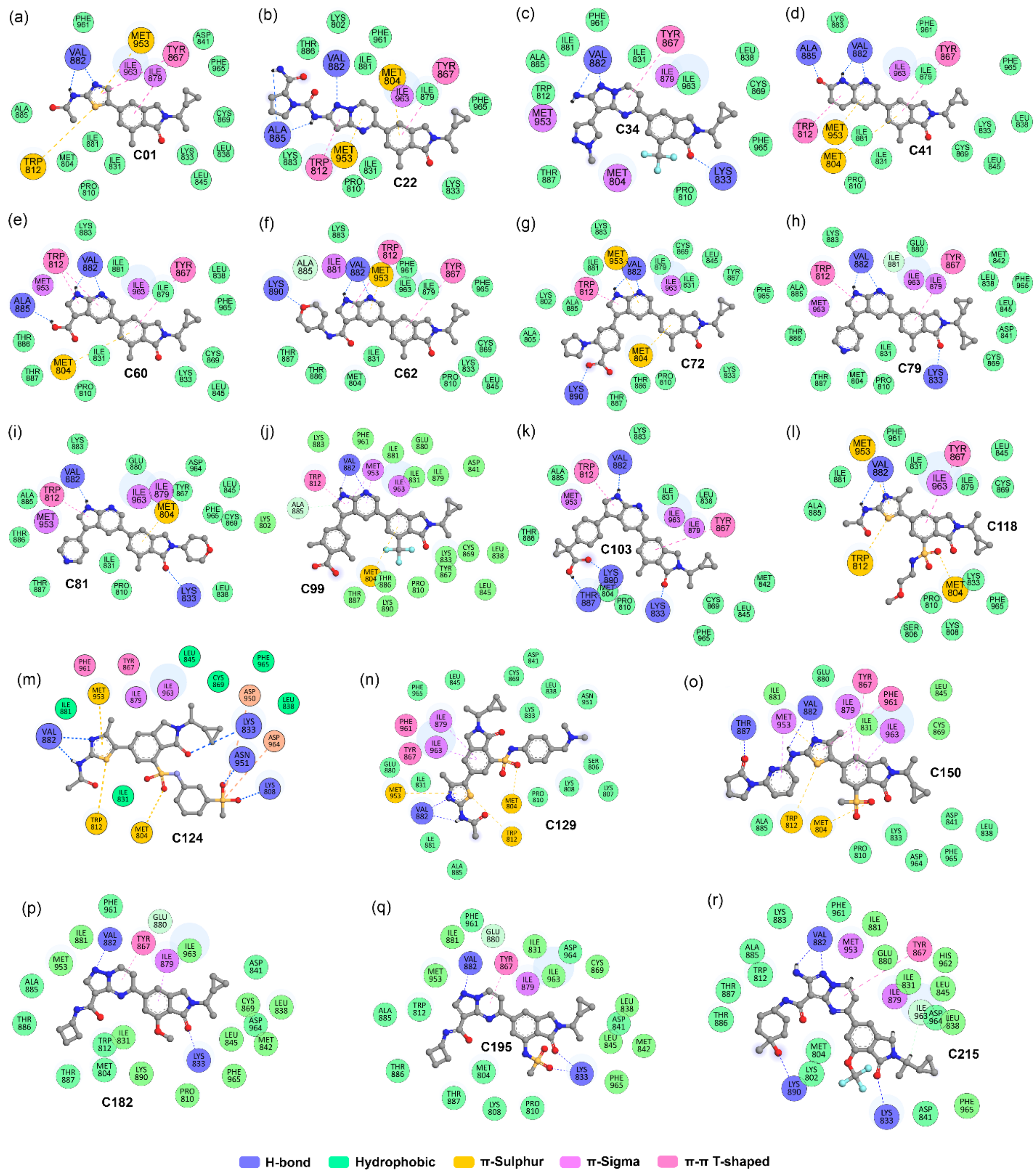

Figure S2. Molecular Docking analysis of the compounds C01, C22, C34, C41, C60, C62, C72, C79, C81, C99, C103, C118, C124, C129, C150, C182, C195, and C215, respectively. The H-bond, hydrophobic, π-Sulphur, π-sigma, and π-π interactions were highlighted by different color schemes by the 2D diagrams.

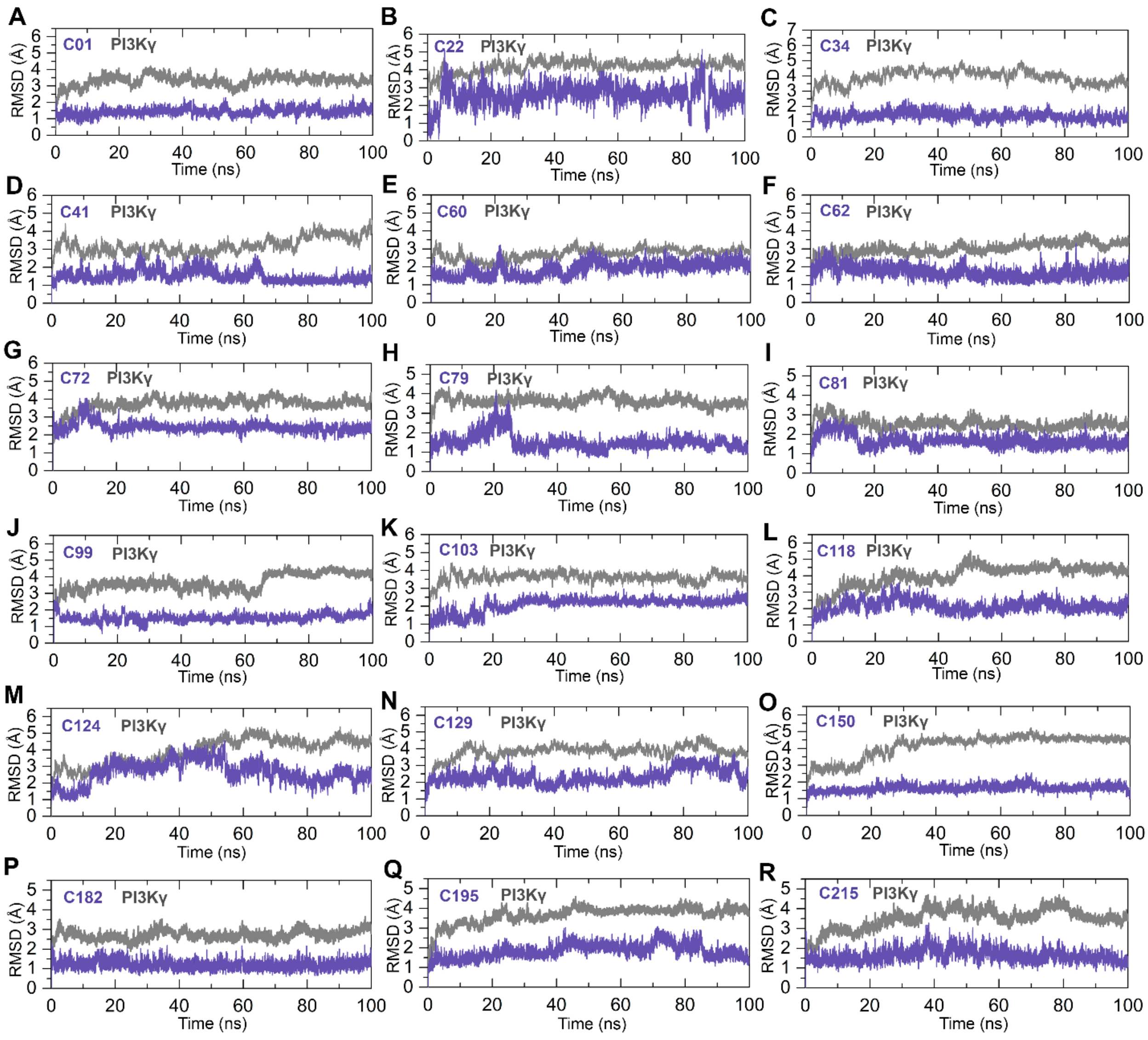

Figure S3. RMSD plots from Molecular Dynamics simulation study of the receptor-ligand complexes. The RMSDs of the ligand and α-carbon of the receptors were shown in slate and grey.

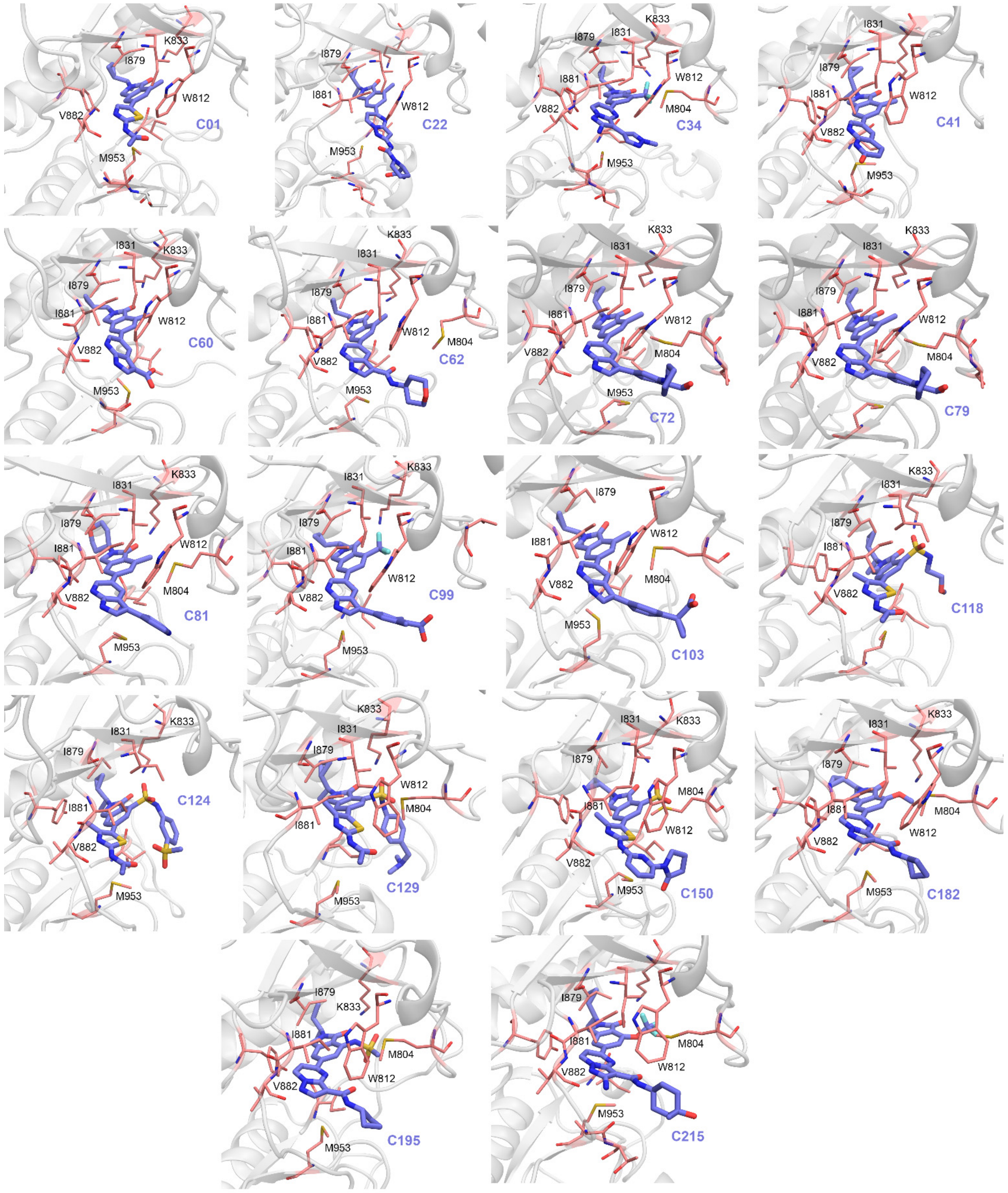

Figure S4. The final 1 ns average MD pose of compounds C01, C22, C34, C41, C60, C62, C72, C79, C81, C99, C103, C118, C124, C129, C150, C182, C195, and C215, respectively, in complex with PI3Kγ. The receptor, interacting residues, and ligands were shown in light-grey, salmon, and slate respectively. The residues are selected from the per-residue MM-PB/GBSA binding energy decomposition analysis (Table 3, in the main text).

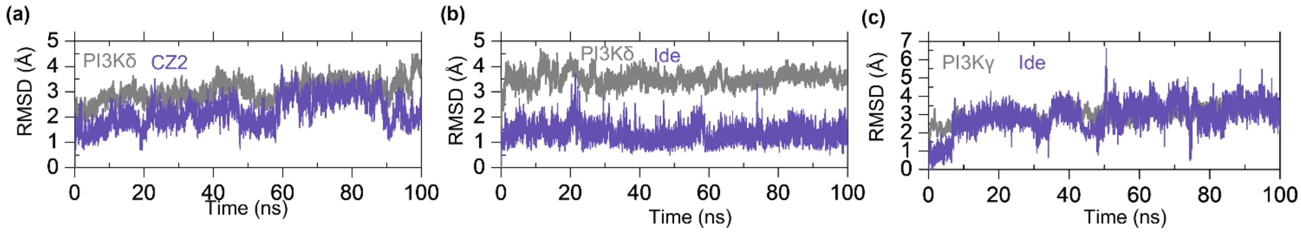

Figure S5. RMSD plots from the Molecular Dynamics simulation study of (a)PI3Kδ-CZ2, (b)PI3Kδ-Idelalisib, and (c) PI3Kγ-Idelalisib complexes.

Table S1: Structure and activity values of isoindolin-1-one based PI3Kγ inhibitors.

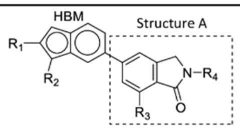

#Cpd	Structure	R_1	R_2	R_3	HBM	R_4	pIC_{50}
01	A	HN, Me, O	-	CH_3	R_1, N, S, Me	Me	8.44
02	A	H_2N	-	CH_3	R_1, H, N, N	Me	NA
03	A	HN, Me, O	-	CH_3	R_1, H, N, N	Me	6.31
04	A	H_2N	-	CH_3	R_1, N, N, N	Me	7.30
05	A	HN, Me, O	-	CH_3	R_1, N, N, N	Me	7.09
06	A	H_2N	-	CH_3	R_1, N, N, N	Me	6.53
07	A	HN, Me, O	-	CH_3	R_1, N, N, N	Me	7.11
08	A	H_2N	H	CH_3	R_1, N, N, N	Me	6.37
09	A	HN, Me, O	H	CH_3	R_1, N, N, N	Me	8.25
10	A	H_2N	Me, N, N	CH_3	R_1, N, N, N	Me	7.76

11	A	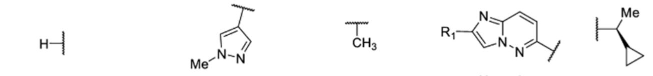	7.82
12	A	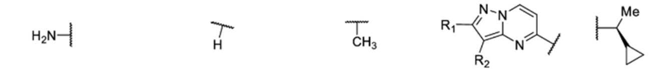	7.22
13	A	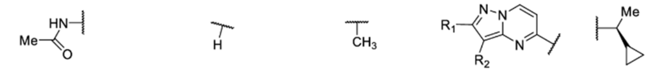	8.35
14	A	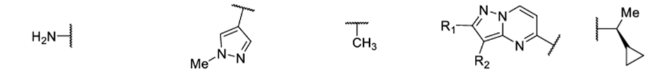	8.53
15	A	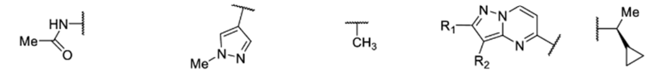	6.36
16	A	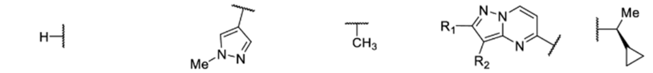	8.04
17	A	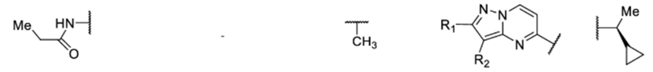	8.13
18	A	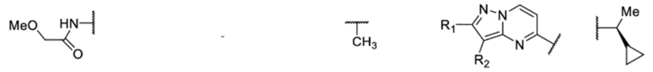	7.85
19	A	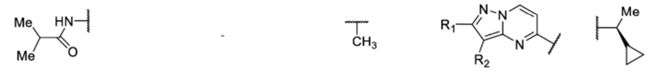	7.15
20	A	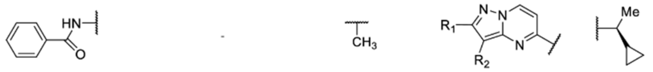	7.03
21	A	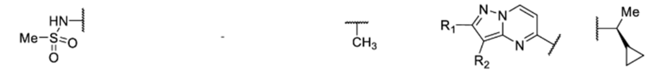	5.27
22	A	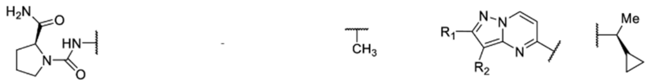	8.53
23	A	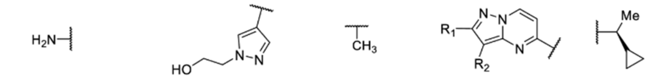	8.19
24	A	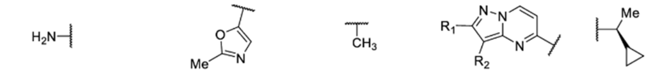	8.22
25	A	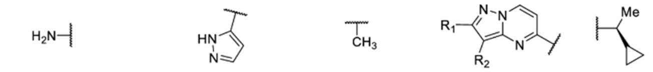	7.35
26	A	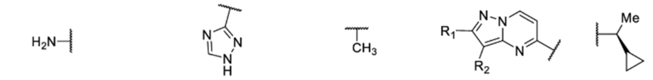	7.50
27	A	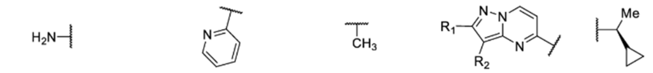	7.19
28	A	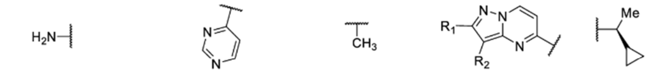	8.12
29	A	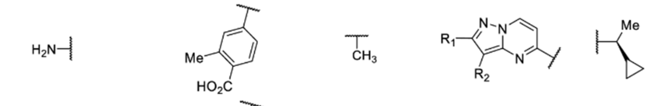	8.65
30	A	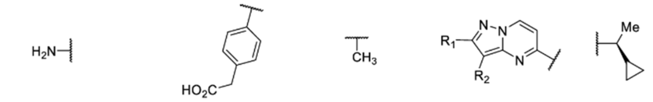	8.67
31	A	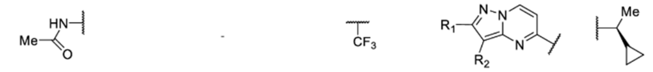	8.65
32	A	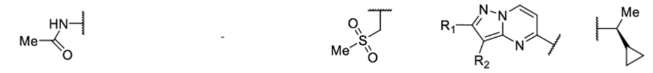	8.88
33	A	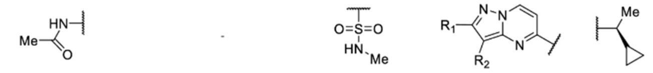	8.56

34	A	H_2N–	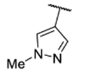	CF_3	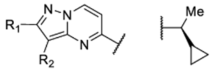	8.72
35	A	H_2N–	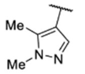	CF_3	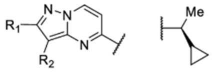	8.18
36	A	H_2N–	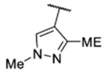	CF_3	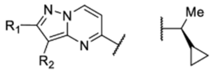	8.09
37	A	H_2N–	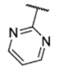	CF_3	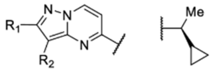	7.27
38	A	H_2N–	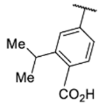	CF_3	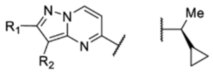	9.00
39	A	H–	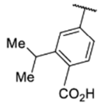	CF_3	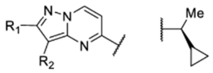	8.67
40	A	-	-	Me	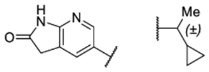	6.22
41	A	-	-	Me	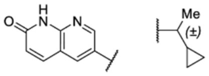	6.27
42	A	-	-	Me	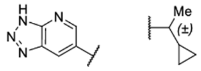	5.79
43	A	-	-	Me	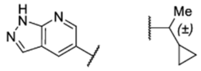	7.07
44	A	-	-	Me	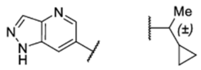	6.30
45	A	-	H–	Me	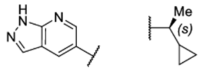	7.45
46	A	-	H–	Me	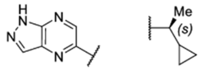	7.09
47	A	-	H–	Me	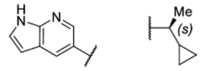	7.30
48	A	-	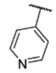	Me	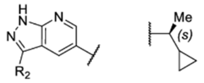	8.09
49	A	-	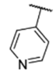	Me	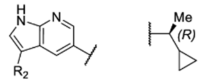	8.46
50	A	-	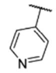	Me	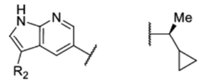	8.04
51	A	-	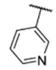	Me	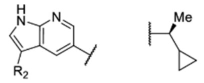	8.15
52	A	-	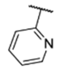	Me	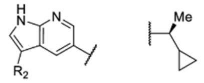	7.48
53	A	-	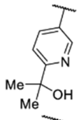	Me	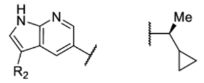	8.48
54	A	-	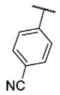	Me	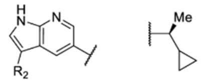	7.37

**55**	A	-	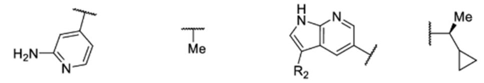	8.23
**56**	A	-	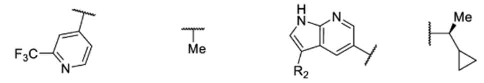	7.79
**57**	A	-	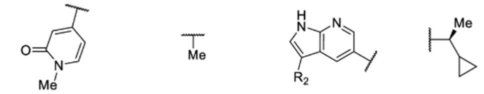	8.58
**58**	A	-	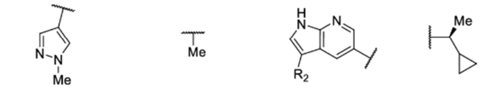	8.48
**59**	A	-	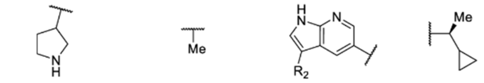	6.55
**60**	A	-	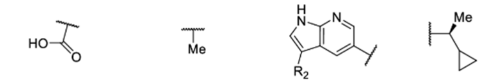	7.88
**61**	A	-	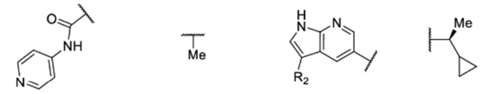	7.92
**62**	A	-	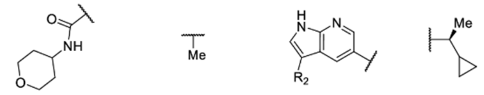	8.34
**63**	A	-	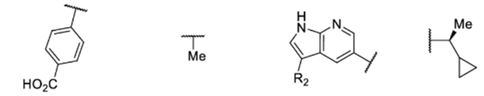	8.60
**64**	A	-	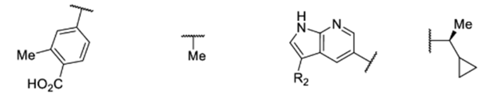	8.55
**65**	A	-	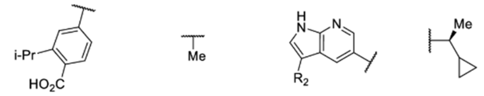	8.67
**66**	A	-	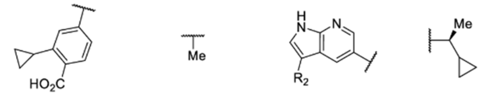	8.79
**67**	A	-	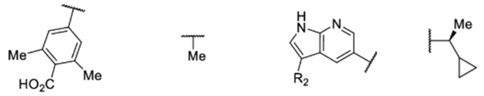	8.65
**68**	A	-	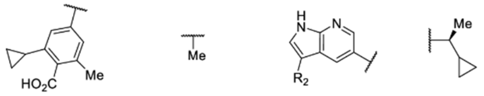	8.92
**69**	A	-	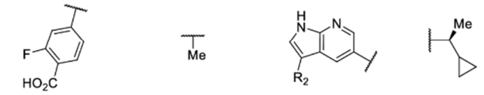	8.50
**70**	A	-	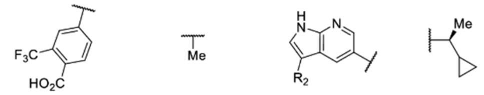	8.78
**71**	A	-	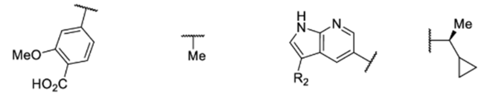	8.82
**72**	A	-	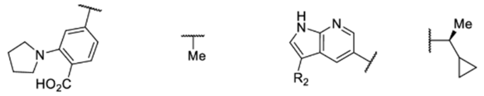	8.39
**73**	A	-	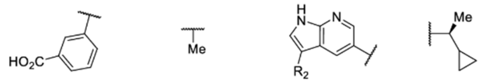	8.56

**74**	A	-	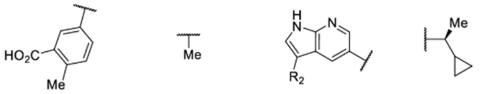				8.30
**75**	A	-	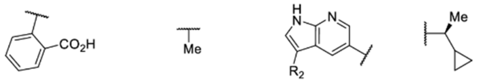				6.74
**76**	A	-	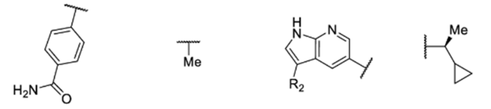				8.58
**77**	A	-	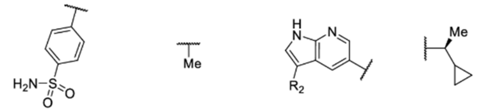				8.30
**78**	A	-	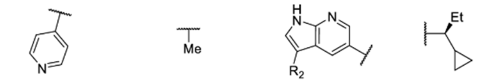				8.22
**79**	A	-	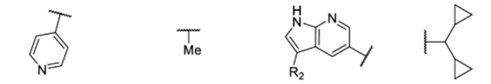				8.16
**80**	A	-	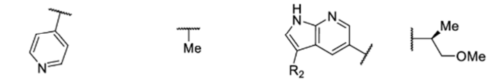				8.04
**81**	A	-	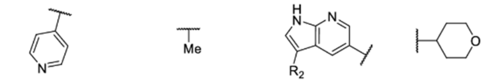				7.30
**82**	A	-	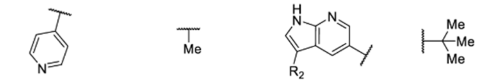				7.58
**83**	A	-	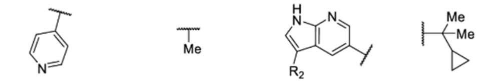				7.55
**84**	A	-	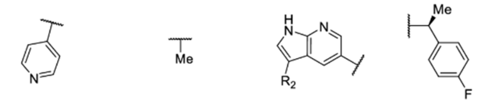				7.39
**85**	A	-	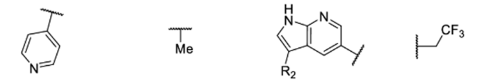				7.59
**86**	A	-	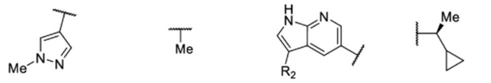				8.48
**87**	A	-	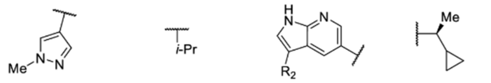				8.37
**88**	A	-	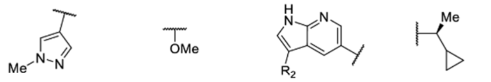				8.36
**89**	A	-	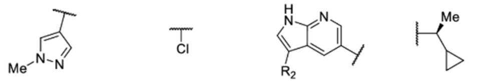				8.61
**90**	A	-	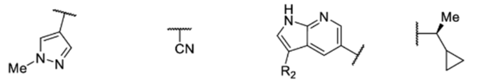				8.43
**91**	A	-	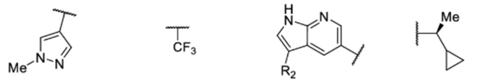				8.61
**92**	A	-	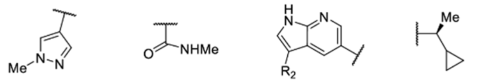				8.1
**93**	A	-	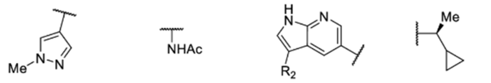				8.60

94	A	-	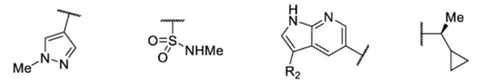		8.39
95	A	-	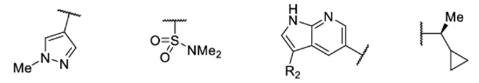		8.58
96	A	-	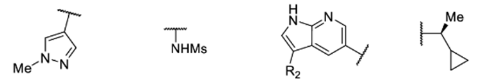		8.39
97	A	-	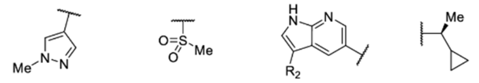		8.82
98	A	-	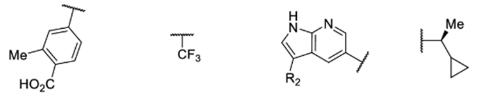		8.92
99	A	-	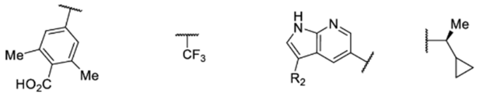		8.55
100	A	-	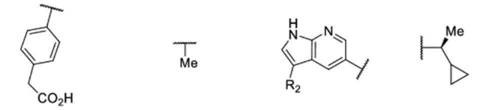		8.58
101	A	-	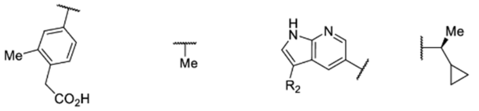		8.48
102	A	-	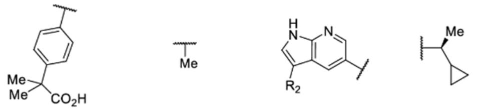		8.67
103	A	-	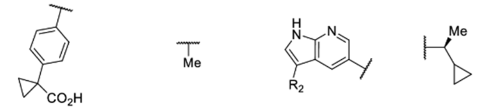		8.48
104	A	-	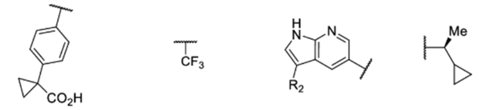		8.44
105	A	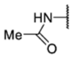	-	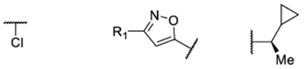	6.80
106	A	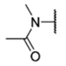	-	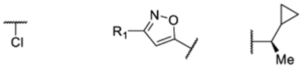	NA
107	A	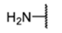	-	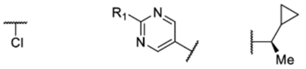	8.00
108	A	-	-	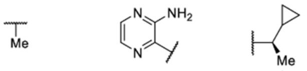	7.60
109	A	-	-	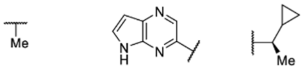	8.20
110	A	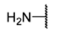	-	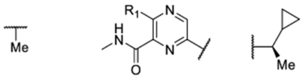	8.10
111	A	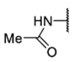	-	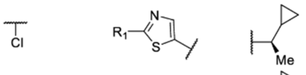	8.90
112	A	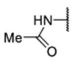	-	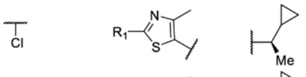	9.10
113	A	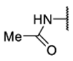	-	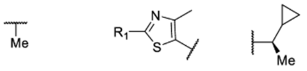	9.20

**114**	A	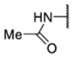	-	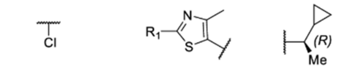	8.90
**115**	A	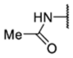	-	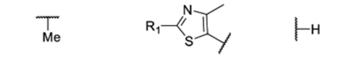	7.20
**116**	A	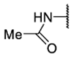	-	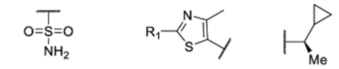	9.20
**117**	A	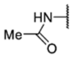	-	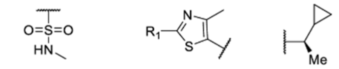	9.10
**118**	A	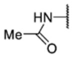	-	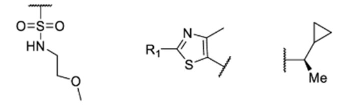	8.90
**119**	A	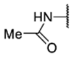	-	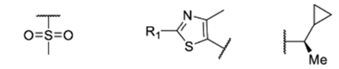	9.10
**120**	A	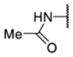	-	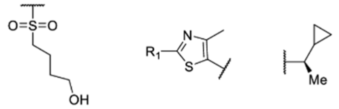	8.90
**121**	A	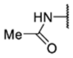	-	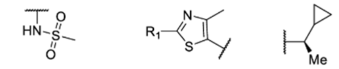	9.20
**122**	A	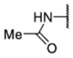	-	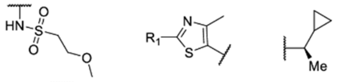	9.00
**123**	A	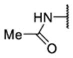	-	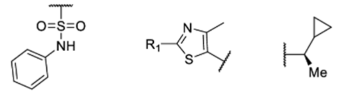	9.0
**124**	A	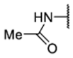	-	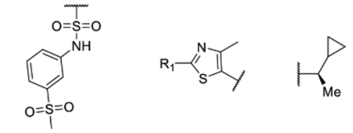	8.9
**125**	A	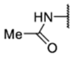	-	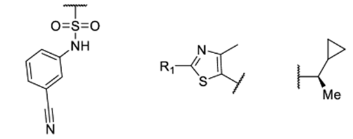	9.1
**126**	A	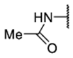	-	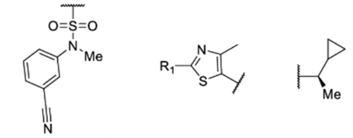	9.1
**127**	A	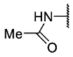	-	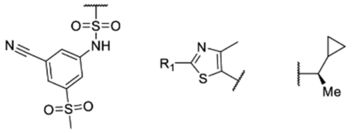	8.9
**128**	A	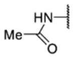	-	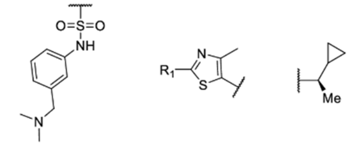	8.7
**129**	A	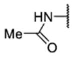	-	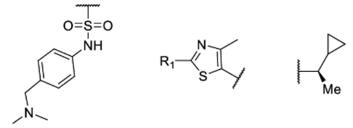	8.6

**130**	A	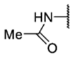	-	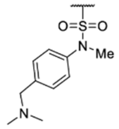	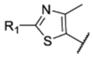	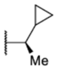	8.6
**131**	A	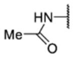	-	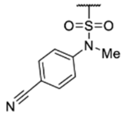	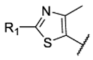	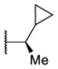	9.0
**132**	A	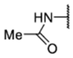	-	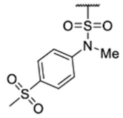	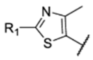	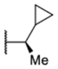	9.0
**133**	A	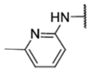	-	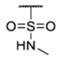	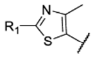	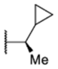	9.0
**134**	A	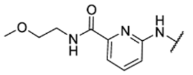	-	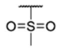	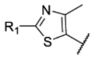	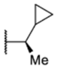	9.1
**135**	A	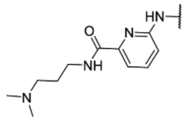	-	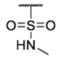	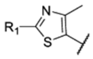	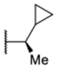	8.5
**136**	A	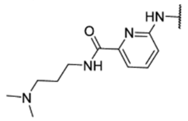	-	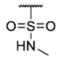	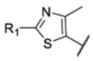	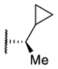	8.9
**137**	A	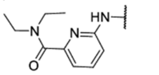	-	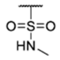	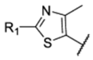	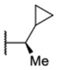	8.8
**138**		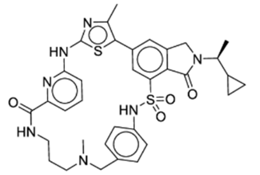					8.1
**139**		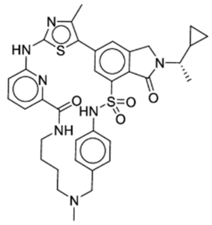					8.0
**140**		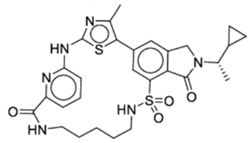					8.6
**141**		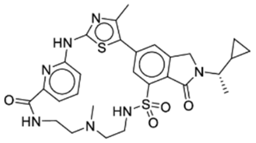					8.9
**142**		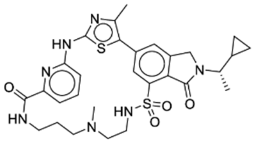					8.8

143		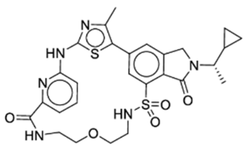			9.1
144	A	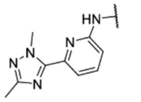	-	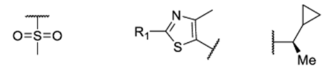	9.0
145	A	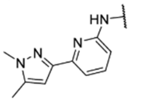	-	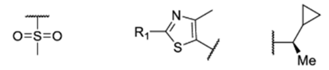	9.0
146	A	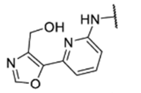	-	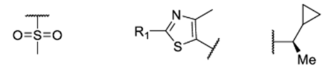	9.2
147	A	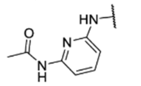	-	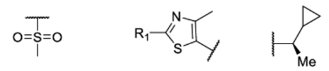	9.0
148	A	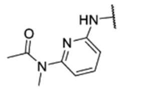	-	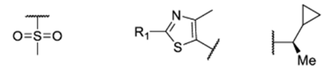	9.1
149	A	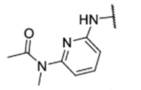	-	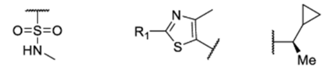	9.1
150	A	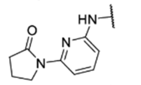	-	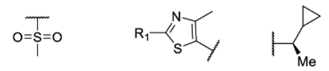	9.1
151	A	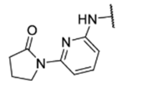	-	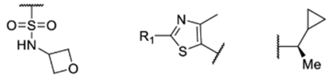	8.9
152	A	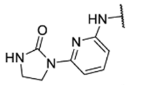	-	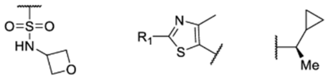	9.0
153	A	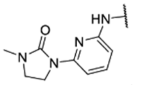	-	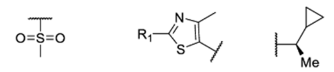	8.9
154	A	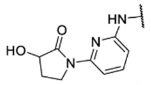	-	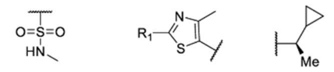	9.0
155	A	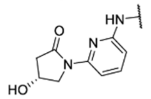	-	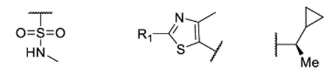	9.0
156	A	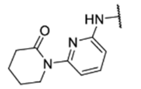	-	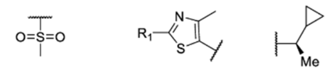	9.0
157	A	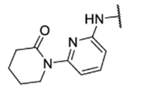	-	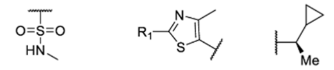	9.0
158	A	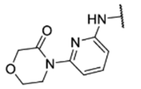	-	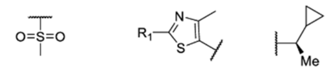	9.1
159	A	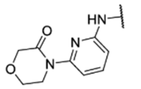	-	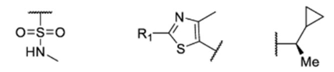	9.1
160	A	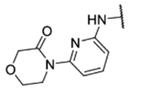	-	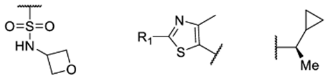	9.0
161	A	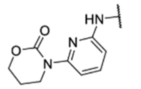	-	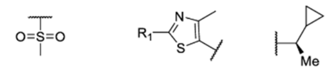	8.9

162	A	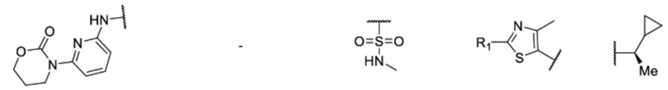	9.0
163	A	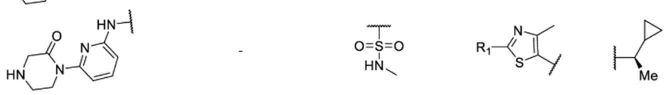	8.9
164	A	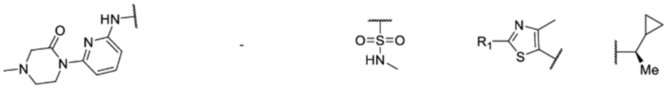	9.0
165	A	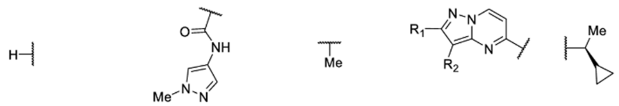	7.46
166		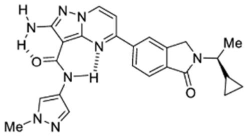	8.46
167	A	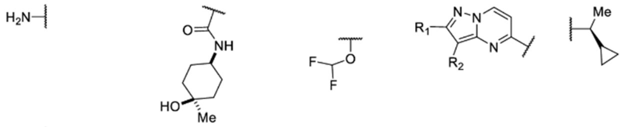	7.46
168	A	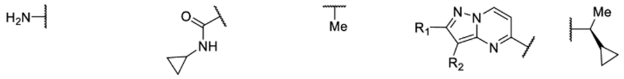	8.44
169	A	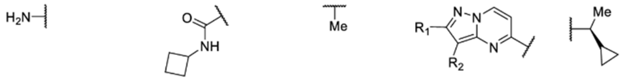	8.63
170	A	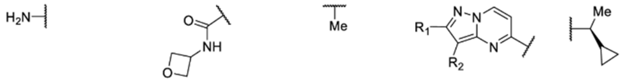	8.74
171	A	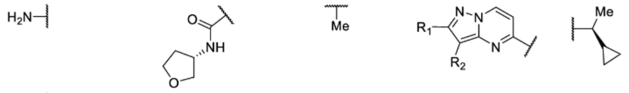	8.74
172	A	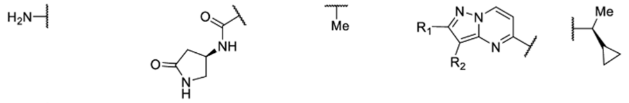	8.74
173	A	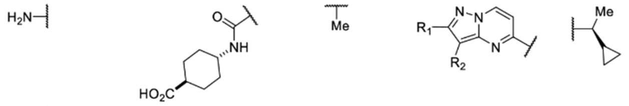	8.67
174	A	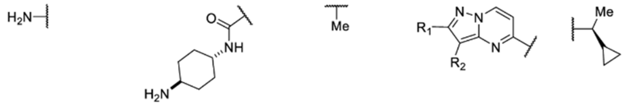	8.69
175	A	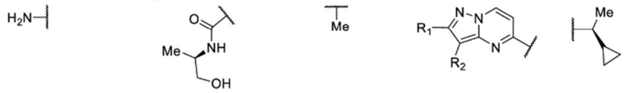	8.45
176	A	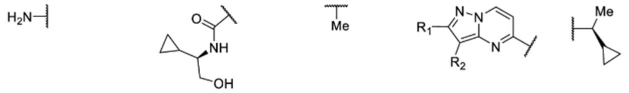	8.53
177	A	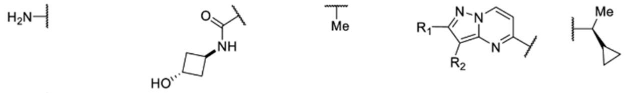	8.72
178	A	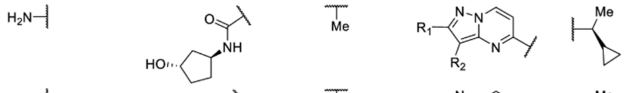	8.37
179	A	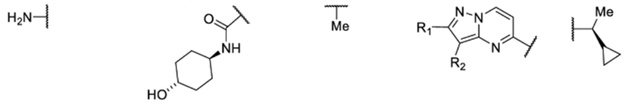	8.92

180	A	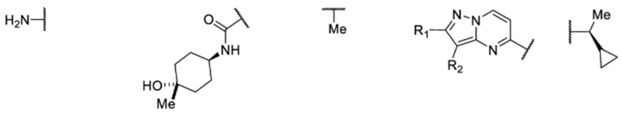	8.52
181	A	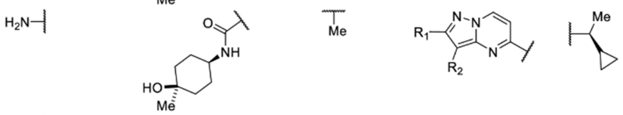	8.31
182	A	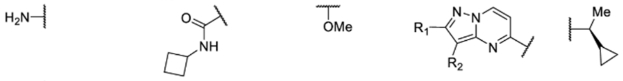	8.42
183	A	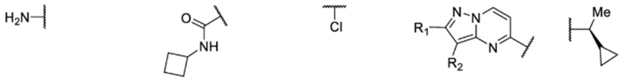	8.61
184	A	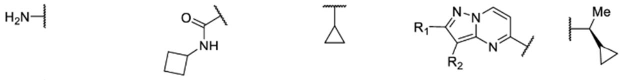	8.32
185	A	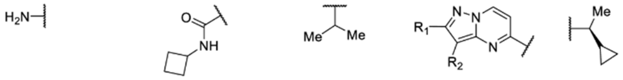	8.33
186	A	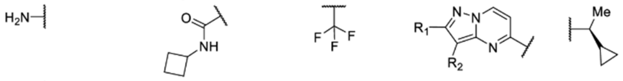	8.44
187	A	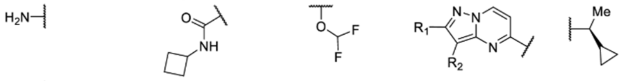	8.74
188	A	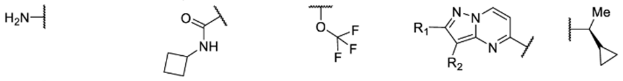	8.44
189	A	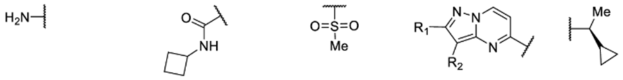	8.61
190	A	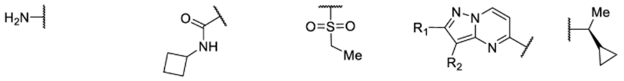	8.31
191	A	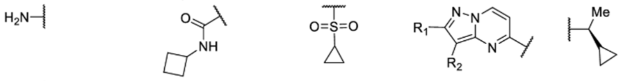	8.76
192	A	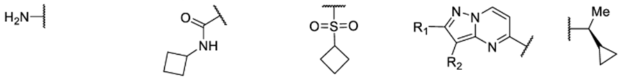	8.52
193	A	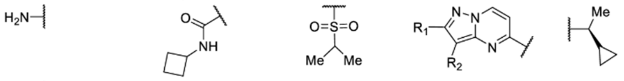	8.37
194	A	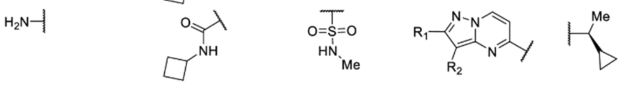	8.56
195	A	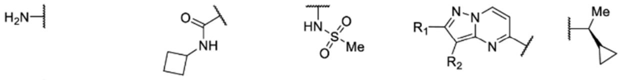	8.56
196	A	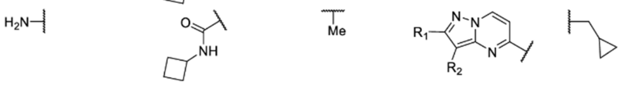	7.99
197	A	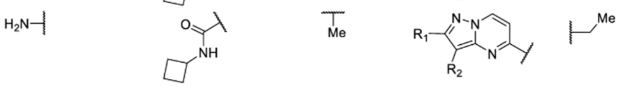	8.25
198	A	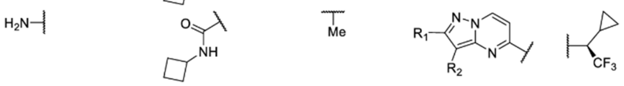	7.75
199	A	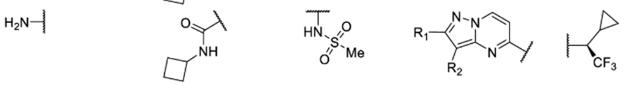	8.76
200	A	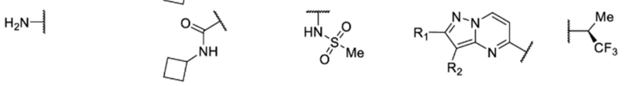	9.09

201	A	H2N—	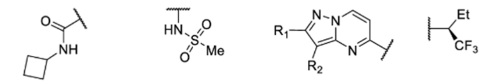	8.48
202	A	H2N—	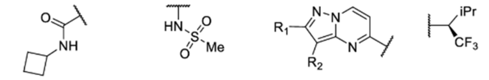	8.07
203	A	H2N—	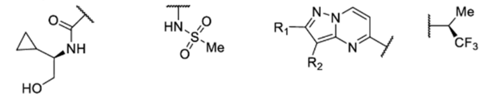	8.45
204	A	H2N—	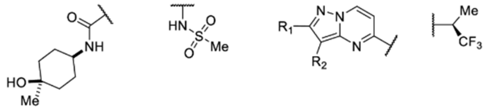	8.49
205	A	H2N—	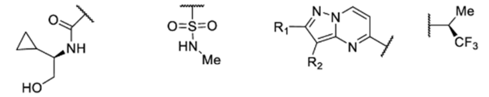	8.88
206	A	H2N—	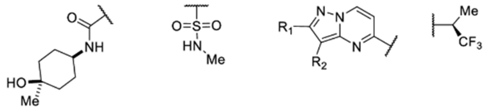	9.04
207	A	H2N—	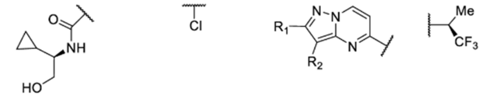	8.5
208	A	H2N—	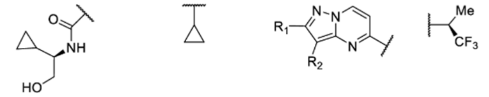	8.6
209	A	H2N—	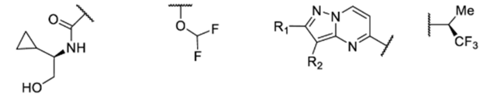	8.46
210	A	H2N—	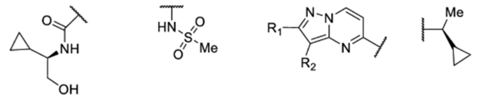	9.09
211	A	H2N—	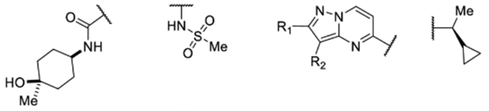	9.04
212	A	H2N—	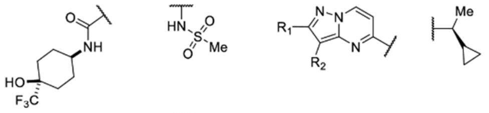	8.67
213	A	H2N—	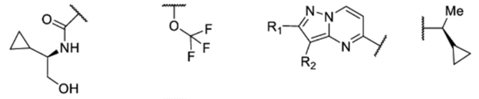	8.55
214	A	H2N—	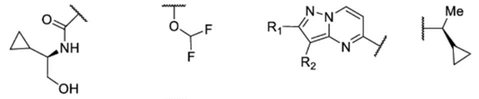	8.5
215	A	H2N—	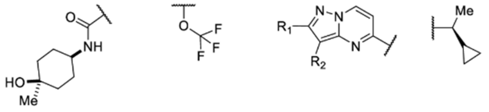	8.55

#Cpd: Compounds

Low piC_{50}	Medium piC_{50}	High piC_{50}	
0.01765 C16	0.00086 C135	0.023074 C133	
0.033976 C79	0.005611 C99	0.031989 C211	
0.059913 C36	0.005853 C169	0.037339 C146	
0.065843 C27	0.031875 C194	0.059498 C111	
0.066136 C92	0.060205 C214	0.061911 C132	
0.095008 C37	0.067761 C30	0.066305 C68	
0.100538 C45	0.072359 C100	0.068893 C122	
0.106822 C198	0.107424 C14	0.069315 C38	Set_A
0.109345 C05	0.121919 C91	0.077651 C170	Test Set
0.138942 C105	0.124881 C180	0.079698 C160	
0.147638 C85	0.136643 C76	0.097758 C200	
0.14874 C56	0.139579 C89	0.1092 C191	
0.162259 C139	0.150753 C53	0.132456 C162	
0.175384 C55	0.164347 C213	0.136551 C136	
0.187422 C196	0.183509 C01	0.139506 C131	
0.204673 C60	0.194492 C204	0.210311 C153	
0.205061 C10	0.226091 C209	0.218177 C128	
0.206046 C59	0.228355 C94	0.218648 C113	
0.212193 C181	0.264171 C57	0.219092 C97	
0.227279 C54	0.269907 C73	0.225364 C156	
0.259206 C03	0.27226 C212	0.247291 C154	
0.279573 C09	0.279446 C182	0.255794 C179	
0.281645 C51	0.289754 C58	0.267802 C127	
0.319439 C18	0.309622 C64	0.280574 C172	
0.329823 C77	0.320964 C102	0.296325 C205	
0.332477 C20	0.325783 C33	0.334168 C149	
0.343475 C46	0.361893 C67	0.365883 C120	Set_B
0.365132 C75	0.367123 C31	0.397083 C117	Test Set
0.405358 C23	0.373521 C176	0.453954 C32	
0.417724 C26	0.378487 C90	0.465152 C144	
0.420072 C82	0.385211 C86	0.47444 C145	
0.423577 C44	0.390515 C29	0.505028 C119	
0.426897 C12	0.39193 C184	0.522105 C187	
0.443717 C138	0.400025 C208	0.5489 C159	
0.447084 C202	0.41186 C168	0.561856 C151	

Low piC_{50}	Medium piC_{50}	High piC_{50}	
0.448198 C11	0.412033 C166	0.569628 C71	
0.480936 C83	0.431211 C62	0.577074 C210	
0.49216 C81	0.438617 C104	0.600328 C150	
0.493277 C61	0.44698 C96	0.600501 C121	
0.500965 C48	0.46959 C193	0.606047 C124	
0.502401 C80	0.512813 C22	0.606085 C66	
0.507699 C41	0.519212 C201	0.642962 C65	
0.511569 C52	0.534719 C39	0.660447 C142	Set_C
0.534753 C167	0.568166 C87	0.665153 C34	Test Set
0.54931 C165	0.596092 C189	0.666049 C171	
0.554155 C108	0.601779 C49	0.678306 C161	
0.555269 C17	0.6166 C93	0.682506 C163	
0.558918 C47	0.645673 C13	0.687748 C147	
0.564246 C78	0.668397 C101	0.691397 C148	
0.609308 C35	0.697762 C72	0.699671 C114	
0.628583 C50	0.722362 C215	0.703101 C141	
0.648751 C28	0.726918 C130	0.70669 C199	
0.651622 C19	0.749068 C183	0.777984 C118	**common**
0.669656 C08	0.76003 C69	0.786919 C134	
0.684476 C06	0.776172 C129	0.789938 C125	
0.703904 C107	0.779696 C192	0.790672 C155	
0.730612 C115	0.803751 C203	0.793448 C123	
0.755389 C43	0.808174 C173	0.80417 C158	
0.762125 C84	0.810399 C207	0.853321 C174	
0.785481 C197	0.81159 C175	0.856726 C157	
0.799715 C40	0.828184 C88	0.870773 C112	
0.802135 C25	0.838347 C103	0.873265 C137	Set_D
0.805379 C24	0.847056 C63	0.891955 C70	Test Set
0.842419 C109	0.856203 C195	0.910908 C206	
0.874096 C74	0.858105 C186	0.926568 C164	
0.887532 C04	0.869255 C185	0.940145 C143	
0.889019 C07	0.89479 C95	0.952019 C116	
0.894948 C190	0.923106 C140	0.952636 C177	
0.924051 C110	0.938145 C178	0.987712 C126	
0.937485 C15	0.948691 C188	0.997186 C98	
		0.99753 C152	

Figure S6. Random sampling table to select the Test set compounds from the dataset.

Table S2: Brief statistical analysis to generate the best CoMSIA model using various combinations of the descriptor fields.

CoMSIA	q^2	ONC	SEP	r^2	SEE	F-value	Field Contribution				
							S	E	H	A	D
S	0.591	6	0.472	0.719	0.392	87.682	100	-	-	-	-
E	0.464	6	0.541	0.634	0.447	59.378	-	100	-	-	-
H	0.478	6	0.534	0.677	0.420	71.952	-	-	100	-	-
A	0.500	6	0.522	0.683	0.416	74.096	-	-	-	100	-
D	0.415	5	0.563	0.546	0.496	49.802	-	-	-	-	100
SE	0.544	6	0.499	0.714	0.395	85.840	33.3	66.7	-	-	-
EH	0.493	6	0.526	0.705	0.401	81.866	-	56.0	44.0	-	-
EA	0.530	6	0.506	0.725	0.387	90.415	-	49.3	-	50.7	-
ED	0.538	6	0.502	0.718	0.392	87.390	-	50.1	-	50.7	-
SH	0.547	6	0.497	*0.719*	0.319	88.026	35.8	-	64.2	-	-
SA	0.583	6	0.477	0.755	0.366	105.603	37.6	-	-	62.4	-
SD	0.609	6	0.461	0.732	0.382	93.868	32.6	-	-	-	67.4
HA	0.563	6	0.488	0.750	0.369	103.273	-	-	48.6	51.4	-
HD	0.537	6	0.502	0.716	0.394	86.569	-	-	45.2	54.8	-
AD	0.571	6	0.484	0.730	0.383	92.981	-	-	-	45.5	54.5
SHE	0.540	5	0.500	0.699	0.404	96.213	21.6	45.2	33.2	-	-
SEA	0.567	6	0.486	0.762	0.361	109.717	21.8	40.6	-	37.6	
SED	0.617	6	0.457	0.772	0.352	116.566	21.1	35.2	-	-	43.8
EHA	0.546	6	0.497	0.750	0.369	103.141	-	35.3	29.2	35.5	-
EHD	0.564	6	0.487	0.758	0.363	107.761	-	35.4	26.5		38.2
SHA	0.582	6	0.477	0.755	0.366	105.632	21.7	-	35.6	42.7	-
SHD	0.608	6	0.463	0.757	0.364	106.976	22.0	-	30.5	-	47.5
EAD	0.591	6	0.472	0.770	0.354	115.216	-	32.0	-	30.2	37.8
HAD	0.617	6	0.457	0.780	0.346	122.013	-	-	27.9	32.8	39.3
SEHD	0.603	6	0.465	0.768	0.356	113.785	15.9	30.2	19.8	-	34.1
SEHA	0.572	6	0.483	0.746	0.372	100.954	15.8	31.4	23.2	29.6	-
SEAD	0.630	6	0.448	0.784	0.344	123.686	16.0	25.8	-	24.5	33.7
EHAD	0.603	6	0.466	0.786	0.341	126.451	-	25.6	20.1	23.8	30.6
SHAD	0.629	6	0.450	0.783	0.344	124.240	15.8	-	20.5	27.6	36.1
SEHAD	0.620	6	0.455	0.780	0.346	122.054	12.6	22.7	15.2	21.2	28.2

q^2: squared cross-validated correlation coefficient; **ONC**: optimal number of components; **SEP**: standard error of prediction; **r^2**: squared correlation coefficient; **SEE**: standard error of estimation; **F-value**: F-test value; **r^2_{pred}**: predictive r^2; **S**: Steric; **E**: Electrostatic; **H**: Hydrophobic; **A**: H-bond acceptor; **D**: H-bond donor. Green highlighted box showing the final selection of the CoMSIA model.

Table S3: Actual pIC50 vs. Predicted pIC50 of the compounds from the CoMFA and CoMSIA by taking every compound from the dataset.

#Cpd	Actual pIC_{50}	Predicted pIC_{50} (CoMFA)	Residuals	Predicted pIC_{50} (CoMSIA-SEAD)	Residuals
C01	8.44	8.51	-0.07	8.44	8.577
C03	6.31	7.326	-1.016	6.31	6.789
C04	7.3	7.481	-0.181	7.3	7.509
C05	7.09	6.718	0.372	7.09	6.613
C06	6.53	6.6	-0.07	6.53	6.509
C07	7.11	6.648	0.462	7.11	6.547
C08	6.37	6.945	-0.575	6.37	6.835
C09	8.25	8.089	0.161	8.25	8.138
C10	7.76	8.102	-0.342	7.76	8.023
C11	7.82	7.948	-0.128	7.82	8.041
C12	7.22	7.203	0.017	7.22	7.226
C13	8.35	8.177	0.173	8.35	8.184
C14	8.53	8.1	0.43	8.53	8.081
C15	6.36	6.638	-0.278	6.36	7.483
C16	8.04	7.999	0.041	8.04	8.098
C17	8.13	8.475	-0.345	8.13	8.198
C18	7.85	7.179	0.671	7.85	6.969
C19	7.15	6.809	0.341	7.15	7.003
C20	7.03	7.074	-0.044	7.03	7.129
C21	5.27	6.019	-0.749	5.27	6.314
C22	8.53	8.744	-0.214	8.53	8.635
C23	8.19	8.091	0.099	8.19	8.105
C24	8.22	8.012	0.208	8.22	7.852
C25	7.35	7.634	-0.284	7.35	7.629
C26	7.5	7.281	0.219	7.5	7.311
C27	7.19	7.59	-0.4	7.19	7.692
C28	8.12	7.683	0.437	8.12	7.807
C29	8.65	8.515	0.135	8.65	8.354
C30	8.67	8.205	0.465	8.67	8.681
C31	8.65	8.866	-0.216	8.65	8.39
C32	8.88	9.121	-0.241	8.88	8.748
C33	8.56	8.879	-0.319	8.56	8.398
C34	8.72	8.387	0.333	8.72	8.327
C35	8.18	8.6	-0.42	8.18	8.296
C36	8.09	7.689	0.401	8.09	8.221
C37	7.27	7.505	-0.235	7.27	7.827
C38	9	8.988	0.012	9	8.681
C39	8.67	8.738	-0.068	8.67	8.725
C40	6.22	6.232	-0.012	6.22	6.345
C41	6.27	6.579	-0.309	6.27	6.742
C42	5.79	6.157	-0.367	5.79	6.695
C43	7.07	6.936	0.134	7.07	6.559

C44	6.3	6.699	-0.399	6.3	6.626
C45	7.45	7.128	0.322	7.45	6.789
C46	7.09	7.407	-0.317	7.09	6.998
C47	7.3	7.302	-0.002	7.3	7.186
C48	8.09	7.766	0.324	8.09	8.024
C49	8.46	8.097	0.363	8.46	8.47
C50	8.04	8.287	-0.247	8.04	8.7
C51	8.15	7.682	0.468	8.15	7.907
C52	7.48	7.305	0.175	7.48	7.262
C53	8.48	8.27	0.21	8.48	8.58
C54	7.37	8.059	-0.689	7.37	7.873
C55	8.23	8.163	0.067	8.23	8.227
C56	7.79	8.355	-0.565	7.79	7.886
C57	8.58	8.324	0.256	8.58	7.723
C58	8.48	7.817	0.663	8.48	8.009
C59	6.55	7.585	-1.035	6.55	7.587
C60	7.88	7.453	0.427	7.88	7.337
C61	7.92	7.915	0.005	7.92	7.539
C62	8.34	7.989	0.351	8.34	7.778
C63	8.6	8.283	0.317	8.6	8.676
C64	8.55	8.554	-0.004	8.55	8.738
C65	8.67	8.63	0.04	8.67	8.81
C66	8.79	8.692	0.098	8.79	8.776
C67	8.65	8.471	0.179	8.65	8.68
C68	8.92	8.567	0.353	8.92	8.716
C69	8.5	8.482	0.018	8.5	8.6
C70	8.78	8.599	0.181	8.78	8.534
C71	8.82	8.607	0.213	8.82	8.704
C72	8.39	8.625	-0.235	8.39	8.677
C73	8.56	8.291	0.269	8.56	8.367
C74	8.3	8.427	-0.127	8.3	8.404
C75	6.74	7.592	-0.852	6.74	7.249
C76	8.58	8.237	0.343	8.58	8.689
C77	8.3	8.393	-0.093	8.3	8.641
C78	8.22	7.756	0.464	8.22	7.839
C79	8.16	7.638	0.522	8.16	7.821
C80	8.04	7.816	0.224	8.04	7.923
C81	7.3	7.373	-0.073	7.3	7.493
C82	7.58	7.64	-0.06	7.58	7.726
C83	7.55	7.87	-0.32	7.55	7.875
C84	7.39	7.72	-0.33	7.39	7.851
C85	7.59	7.363	0.227	7.59	7.573
C86	8.48	8.144	0.336	8.48	8.067
C87	8.37	8.325	0.045	8.37	8.052
C88	8.36	8.444	-0.084	8.36	8.194
C89	8.61	8.323	0.287	8.61	8.168
C90	8.43	8.456	-0.026	8.43	8.301
C91	8.61	8.402	0.208	8.61	8.301
C92	8.1	8.378	-0.278	8.1	8.234
C93	8.6	8.162	0.438	8.6	8.453
C94	8.39	8.464	-0.074	8.39	8.447
C95	8.58	8.566	0.014	8.58	8.612
C96	8.39	8.546	-0.156	8.39	8.377

C97	8.82	8.486	0.334	8.82	8.694
C98	8.92	8.835	0.085	8.92	8.972
C99	8.55	8.742	-0.192	8.55	8.914
C100	8.58	8.137	0.443	8.58	8.472
C101	8.48	8.46	0.02	8.48	8.552
C102	8.67	8.328	0.342	8.67	8.524
C103	8.48	8.326	0.154	8.48	8.499
C104	8.44	8.6	-0.16	8.44	8.733
C105	6.8	7.577	-0.777	6.8	7.64
C107	8	7.059	0.941	8	7.71
C108	7.6	7.649	-0.049	7.6	7.396
C109	8.2	7.755	0.445	8.2	7.755
C110	8.1	7.967	0.133	8.1	8.322
C111	8.9	8.675	0.225	8.9	8.684
C112	9.1	8.978	0.122	9.1	8.71
C113	9.2	8.811	0.389	9.2	8.606
C114	8.9	8.76	0.14	8.9	8.476
C115	7.2	8.195	-0.995	7.2	8.31
C116	9.2	8.995	0.205	9.2	9.286
C117	9.1	9.125	-0.025	9.1	9.119
C118	8.9	9.154	-0.254	8.9	9.153
C119	9.1	8.994	0.106	9.1	8.804
C120	8.9	9.078	-0.178	8.9	8.919
C121	9.2	9.198	0.002	9.2	8.908
C122	9	9.292	-0.292	9	9.086
C123	9	9.014	-0.014	9	9.024
C124	8.9	8.871	0.029	8.9	8.666
C125	9.1	8.99	0.11	9.1	8.728
C126	9.1	8.939	0.161	9.1	8.975
C127	8.9	8.861	0.039	8.9	8.67
C128	8.7	8.877	-0.177	8.7	8.653
C129	8.6	8.659	-0.059	8.6	8.621
C130	8.6	8.619	-0.019	8.6	8.868
C131	9	8.861	0.139	9	8.954
C132	9	8.757	0.243	9	8.933
C133	9	8.684	0.316	9	8.677
C134	9.1	8.738	0.362	9.1	8.953
C135	8.5	8.63	-0.13	8.5	8.708
C136	8.9	8.839	0.061	8.9	8.679
C137	8.8	8.866	-0.066	8.8	8.924
C138	8.1	8.698	-0.598	8.1	8.523
C139	8	8.504	-0.504	8	8.439
C140	8.6	8.39	0.21	8.6	8.489
C141	8.9	8.458	0.442	8.9	8.547
C142	8.8	8.543	0.257	8.8	8.642
C143	9.1	8.529	0.571	9.1	8.684
C144	9	8.875	0.125	9	9.262
C145	9	9.039	-0.039	9	9.038
C146	9.2	9.145	0.055	9.2	9.204
C147	9	8.994	0.006	9	9.065
C148	9.1	9.083	0.017	9.1	9.184
C149	9.1	8.971	0.129	9.1	9.016
C150	9.1	9.049	0.051	9.1	9.202

C151	8.9	8.917	-0.017	8.9	8.997
C152	9	8.785	0.215	9	8.819
C153	8.9	9.035	-0.135	8.9	8.965
C154	9	9.03	-0.03	9	9.119
C155	9	9.044	-0.044	9	8.92
C156	9	9.171	-0.171	9	9.242
C157	9	9.06	-0.06	9	9.069
C158	9.1	9.128	-0.028	9.1	9.258
C159	9.1	9.033	0.067	9.1	9.073
C160	9	8.79	0.21	9	9.069
C161	8.9	9	-0.1	8.9	9.064
C162	9	8.904	0.096	9	8.879
C163	8.9	9.082	-0.182	8.9	9.006
C164	9	9.097	-0.097	9	9.06
C165	7.46	8.352	-0.892	7.46	8.236
C166	8.46	8.371	0.089	8.46	8.121
C167	7.46	8.336	-0.876	7.46	8.701
C168	8.44	8.232	0.208	8.44	8.335
C169	8.63	8.473	0.157	8.63	8.308
C170	8.74	8.506	0.234	8.74	8.465
C171	8.74	8.479	0.261	8.74	8.652
C172	8.74	8.626	0.114	8.74	8.636
C173	8.67	8.447	0.223	8.67	8.606
C174	8.69	8.455	0.235	8.69	8.489
C175	8.45	8.447	0.003	8.45	8.793
C176	8.53	8.298	0.232	8.53	8.794
C177	8.72	8.503	0.217	8.72	8.453
C178	8.37	8.546	-0.176	8.37	8.721
C179	8.92	8.511	0.409	8.92	8.498
C180	8.52	8.378	0.142	8.52	8.716
C181	8.31	8.431	-0.121	8.31	8.682
C182	8.42	8.633	-0.213	8.42	8.294
C183	8.61	8.635	-0.025	8.61	8.411
C184	8.32	8.697	-0.377	8.32	8.289
C185	8.33	8.67	-0.34	8.33	8.285
C186	8.44	8.712	-0.272	8.44	8.541
C187	8.74	8.807	-0.067	8.74	8.565
C188	8.44	8.714	-0.274	8.44	8.486
C189	8.61	8.694	-0.084	8.61	8.483
C190	8.31	8.668	-0.358	8.31	8.471
C191	8.76	8.749	0.011	8.76	8.529
C192	8.52	8.725	-0.205	8.52	8.512
C193	8.37	8.742	-0.372	8.37	8.507
C194	8.56	8.596	-0.036	8.56	8.613
C195	8.56	8.79	-0.23	8.56	8.497
C196	7.99	8.422	-0.432	7.99	8.458
C197	8.25	8.065	0.185	8.25	8.2
C198	7.75	8.449	-0.699	7.75	8.376
C199	8.76	8.656	0.104	8.76	8.451
C200	9.09	8.636	0.454	9.09	8.288
C201	8.48	8.676	-0.196	8.48	8.398
C202	8.07	8.715	-0.645	8.07	8.456
C203	8.45	8.679	-0.229	8.45	8.448

C204	8.49	8.588	-0.098	8.49	8.578
C205	8.88	8.646	0.234	8.88	8.694
C206	9.04	8.472	0.568	9.04	8.569
C207	8.5	8.629	-0.129	8.5	8.38
C208	8.6	8.617	-0.017	8.6	8.24
C209	8.46	8.663	-0.203	8.46	8.347
C210	9.09	8.772	0.318	9.09	8.783
C211	9.04	8.67	0.37	9.04	8.943
C212	8.67	8.598	0.072	8.67	9.042
C213	8.55	8.769	-0.219	8.55	8.741
C214	8.5	8.746	-0.246	8.5	8.664
C215	8.55	8.634	-0.084	8.55	8.759

#Cpd: Compounds.

Table S4: Statistical analysis to generate the best CoMSIA model using various combinations of the descriptors field for SET-A compounds.

CoMSIA	q^2	ONC	SEP	r^2	SEE	F-value	Field Contribution				
							S	E	H	A	D
**S**	0.616	6	0.480	0.766	0.375	82.828	100	-	-	-	-
**E**	0.478	6	0.560	0.703	0.423	59.865	-	100	-	-	-
**H**	0.511	6	0.542	0.731	0.402	68.840	-	-	100	-	-
**A**	0.494	6	0.552	0.706	0.420	60.942	-	-	-	100	-
**D**	0.421	4	0.586	0.548	0.518	46.691	-	-	-	-	100
**SE**	0.583	6	0.501	0.776	0.3367	87.592	31.9	68.1	-	-	-
**EH**	0.532	6	0.531	0.754	0.384	77.839	-	58.3	41.7	-	-
**EA**	0.560	6	0.514	0.770	0.372	84.581	-	49.4	-	50.6	-
**ED**	0.565	6	0.512	0.758	0.382	79.305	-	50.8	-	-	49.2
**SH**	0.591	6	0.496	0.778	0.365	88.835	36.3	-	63.7	-	-
**SA**	0.619	6	0.478	0.813	0.336	109.813	39.9	-	-	60.1	-
**SD**	0.612	5	0.481	0.749	0.387	91.296	31.8	-	-	-	60.1
**HA**	0.601	6	0.490	0.806	0.342	105.280	-	-	50.8	49.2	-
**HD**	0.523	6	0.535	0.748	0.389	75.262	-	-	44.9	-	55.1
**AD**	0.546	6	0.523	0.756	0.383	78.698	-	-	-	46.3	53.7
**SHE**	0.576	6	0.505	0.788	0.357	94.037	22.8	46.5	31.8	-	-
**SEA**	0.619	6	0.479	0.819	0.330	114.369	22.8	40.1	-	37.0	-
**SED**	0.653	6	0.457	0.817	0.332	113.148	20.6	35.8	-	-	43.6
**EHA**	0.582	5	0.500	0.765	0.375	99.712	-	37.3	25.4	36.8	-
**EHD**	0.597	6	0.492	0.796	0.350	98.719	-	37.5	25.4	-	37.1
**SHA**	0.639	6	0.466	0.818	0.331	114.369	23.3	-	35.9	40.8	-
**SHD**	0.615	6	0.481	0.797	0.349	99.434	22.3	-	29.3	-	48.4
**EAD**	0.598	6	0.492	0.800	0.347	101.136	-	34.0	-	30.2	35.8
**HAD**	0.607	6	0.486	0.810	0.338	108.206	-	-	28.9	32.6	38.5
**SEHD**	0.641	6	0.465	0.819	0.330	114.617	16.1	30.5	18.5	-	34.9
**SEHA**	0.623	6	0.476	0.806	0.341	105.567	16.0	31.4	21.6	31.0	-
**SEAD**	0.655	5	0.454	0.804	0.342	125.375	15.9	26.4	-	26.7	31.0
**EHAD**	0.622	5	0.483	0.827	0.322	121.322	-	27.0	20.2	23.5	29.3
**SHAD**	0.641	6	0.465	0.827	0.323	120.848	17.3	-	21.2	27.2	34.3
**SEHAD**	0.652	6	0.457	0.826	0.324	120.235	13.5	23.6	14.7	21.6	26.6

Table S5: Statistical analysis to generate the best CoMSIA model using various combinations of the descriptors field for SET-B compounds.

CoMSIA	q^2	ONC	SEP	r^2	SEE	F-value	Field Contribution				
							S	E	H	A	D
S	0.606	6	0.464	0.762	0.361	81.023	100	-	-	-	-
E	0.430	5	0.556	0.661	0.430	49.362	-	100	-	-	-
H	0.458	4	0.541	0.612	0.457	60.831	-	-	100	-	-
A	0.420	6	0.563	0.685	0.415	55.128	-	-	-	100	-
D	0.401	5	0.570	0.571	0.482	40.765	-	-	-	-	100
SE	0.539	5	0.500	0.714	0.394	76.352	36.4	63.6			
EH	0.483	4	0.528	0.632	0.446	65.980		60.7	39.3		
EA	0.481	5	0.530	0.673	0.421	62.939		49.5		50.5	
ED	0.503	6	0.521	0.742	0.375	72.981		52.7			47.3
SH	0.543	6	0.499	0.752	0.368	76.673	37.3		62.7		
SA	0.537	6	0.503	0.777	0.349	88.217	38.7			61.3	
SD	0.604	6	0.465	0.763	0.360	81.648	33.6				66.4
HA	0.489	4	0.525	0.656	0.431	73.405			46.9	53.1	
HD	0.513	6	0.516	0.732	0.383	69.120			43.7		56.3
AD	0.529	6	0.507	0.761	0.361	80.690				45.5	54.5
SHE	0.530	4	0.504	0.676	0.418	80.398	24.5	47.4	28.1	-	-
SEA	0.541	5	0.499	0.744	0.373	88.842	23.4	42.5		34.1	
SED	0.608	5	0.461	0.788	0.339	113.465	23.1	37.4			39.4
EHA	0.506	4	0.516	0.692	0.408	86.402		40.5	26.3	33.2	
EHD	0.530	5	0.505	0.756	0.364	94.689		39.1	25.8		35.2
SHA	0.530	6	0.507	0.776	0.350	87.667	23.3		36.4	40.3	
SHD	0.597	6	0.469	0.789	0.339	94.986	23.6		29.2		47.2
EAD	0.536	5	0.502	0.735	0.379	85.066		31.7		32.0	36.3
HAD	0.567	6	0.486	0.800	0.330	101.466			26.9	31.7	41.4
SEHD	0.579	6	0.480	0.801	0.330	101.841	17.8	31.5	17.5		33.2
SEHA	0.535	6	0.504	0.778	0.348	88.668	18.2	33.7	22.1	26.0	-
SEAD	0.594	6	0.471	0.810	0.322	108.147	17.5	28.1		22.2	32.1
EHAD	0.551	5	0.494	0.768	0.355	101.428		27.9	19.0	23.9	29.2
SHAD	0.595	6	0.470	0.816	0.317	112.472	16.5		20.5	26.1	36.9
SEHAD	0.579	6	0.480	0.806	0.325	105.483	14.2	24.9	14.5	18.9	27.5

Table S6: Statistical analysis to generate the best CoMSIA model using various combinations of the descriptors field for SET-C compounds.

CoMSIA	q^2	ONC	SEP	r^2	SEE	F-value	Field Contribution				
							S	E	H	A	D
**S**	0.610	6	0.490	0.762	0.383	81.099	100	-	-	-	-
**E**	0.459	6	0.577	0.684	0.441	54.858	-	100	-	-	-
**H**	0.446	6	0.584	0.697	0.432	58.180	-	-	100	-	-
**A**	0.468	6	0.572	0.713	0.420	63.042	-	-	-	100	-
**D**	0.390	5	0.611	0.595	0.498	44.988	-	-	-	-	100
**SE**	0.533	6	0.536	0.743	0.398	73.281	33.4	66.6	-	-	-
**EH**	0.474	6	0.569	0.737	0.402	71.155	-	54.8	45.2	-	-
**EA**	0.517	5	0.551	0.699	0.429	71.159	-	49.3	-	50.7	-
**ED**	0.540	6	0.532	0.776	0.371	87.991	-	49.2	-	-	50.8
**SH**	0.519	6	0.544	0.742	0.399	72.675	36.7	-	63.3	-	-
**SA**	0.548	6	0.527	0.793	0.357	96.816	36.9	-	-	63.1	-
**SD**	0.607	6	0.492	0.775	0.372	87.143	32.1	-	-	-	67.9
**HA**	0.525	5	0.539	0.719	0.414	78.426	-	-	47.6	52.4	-
**HD**	0519	6	0.544	0.756	0.388	78.374	-	-	43.6	-	56.4
**AD**	0.549	6	0.527	0.766	0.380	82.914	-	-	-	44.8	55.2
**SHE**	0.513	6	0.548	0.745	0.396	74.094	21.8	44.5	33.7	-	-
**SEA**	0.550	5	0.525	0.742	0.397	87.968	20.1	41.3	-	38.6	-
**SED**	0.610	5	0.489	0.788	0.360	113.508	21.4	34.7	-	-	43.9
**EHA**	0.531	5	0.535	0.739	0.400	86.555	-	36.5	28.2	35.3	-
**EHD**	0.552	5	0.523	0.771	0.375	102.843	-	36.7	25.9	-	37.4
**SHA**	0.539	6	0.533	0.781	0.367	90.539	20.9	-	36.3	42.8	-
**SHD**	0.583	6	0.507	0.784	0.365	91.691	21.3	-	30.3	-	48.4
**EAD**	0.568	6	0.516	0.805	0.346	104.642	-	32.0	-	29.6	38.4
**HAD**	0.597	6	0.498	0.813	0.340	109.959	-	-	27.2	32.7	40.1
**SEHD**	0.580	6	0.508	0.799	0.352	100.898	15.1	28.9	20.3	-	35.7
**SEHA**	0.540	6	0.532	0.768	0.378	83.932	15.1	31.6	23.4	29.9	-
**SEAD**	0.603	6	0.494	0.814	0.338	111.033	15.3	25.7	-	24.4	34.6
**EHAD**	0.581	6	0.508	0.815	0.338	111.366	-	25.7	19.8	23.6	30.8
**SHAD**	0.602	6	0.495	0.810	0.342	108.035	14.7	-	20.2	28.0	37.1
**SEHAD**	0.591	6	0.502	0.809	0.343	107.409	11.8	22.2	15.7	21.4	28.8

Table S7: Statistical analysis to generate the best CoMSIA model using various combinations of the descriptors field for SET-D compounds.

**CoMSIA**	q^2	**ONC**	**SEP**	r^2	**SEE**	**F-value**	**Field Contribution**				
							**S**	**E**	**H**	**A**	**D**
**S**	0.495	6	0.514	0.690	0.402	56.357	100	-	-	-	-
**E**	0.338	6	0.588	0.614	0.449	40.245	-	100	-	-	-
**H**	0.396	6	0.562	0.697	0.398	58.368	-	-	100	-	-
**A**	0.405	6	0.557	0.652	0.426	47.475	-	-	-	100	-
**D**	0.340	6	0.587	0.606	0.454	38.981	-	-	-	-	100
**SE**	0.450	6	0.536	0.698	0.397	58.593	34.2	65.8	-	-	-
**EH**	0.407	6	0.556	0.704	0.393	60.138	-	54.6	45.4	-	-
**EA**	0.447	6	0.537	0.734	0.373	69.737	-	46.6	-	53.4	-
**ED**	0.442	6	0.540	0.724	0.379	66.545	-	46.1	-	-	53.9
**SH**	0.460	6	0.531	0.716	0.385	63.973	35.1	-	64.9	-	-
**SA**	0.499	6	0.511	0.744	0.366	73.524	34.0	-	-	66.0	-
**SD**	0.568	6	0.475	0.754	0.359	77.440	30.5	-	-	-	69.5
**HA**	0.496	6	0.513	0.763	0.352	81.554	-	-	47.9	52.1	-
**HD**	0.477	6	0.523	0.743	0.366	73.249	-	-	44.6	-	55.4
**AD**	0.495	6	0.513	0.738	0.370	71.375	-	-	-	43.7	56.3
**SHE**	0.447	6	0.537	0.718	0.384	64.464	22.1	44.2	33.7	-	-
**SEA**	0.501	6	0.510	0.763	0.352	81.463	21.0	38.6	-	40.4	-
**SED**	0.538	5	0.489	0.787	0.334	93.450	20.6	33.1	-	-	46.3
**EHA**	0.470	6	0.526	0.757	0.357	78.755	-	33.3	29.3	37.4	-
**EHD**	0.482	6	0.520	0.767	0.349	83.331	-	34.0	27.0	-	39.1
**SHA**	0.517	6	0.502	0.757	0.357	78.737	21.2	-	35.1	43.7	-
**SHD**	0.566	6	0.476	0.783	0.337	91.481	21.3	-	29.9	-	48.7
**EAD**	0.516	6	0.503	0.781	0.338	90.312	-	31.0	-	30.0	39.0
**HAD**	0.548	6	0.486	0.785	0.335	92.707	-	-	27.9	33.5	38.6
**SEHD**	0.533	6	0.494	0.777	0.341	88.414	15.8	28.4	20.3	-	35.6
**SEHA**	0.502	6	0.510	0.754	0.359	77.500	15.8	29.2	24.0	31.0	-
**SEAD**	0.566	6	0.476	0.790	0.331	95.264	15.3	23.5	-	25.7	35.4
**EHAD**	0.527	6	0.497	0.798	0.325	100.124	-	24.8	19.5	24.8	30.9
**SHAD**	0.581	6	0.468	0.796	0.326	98.962	15.7	-	20.9	28.4	35.1
**SEHAD**	0.555	6	0.482	0.790	0.331	95.554	12.5	20.8	15.7	22.1	28.9

q^2: squared cross-validated correlation coefficient; **ONC**: optimal number of components; **SEP**: standard error of prediction; r^2: squared correlation coefficient; **SEE**: standard error of estimation; **F-value**: F-test value; r^2_{pred}: predictive r^2; **S**: Steric; **E**: Electrostatic; **H**: Hydrophobic; **A**: H-bond acceptor; **D**: H-bond donor. Green highlighted box showing the final selection of the CoMSIA model.

Table S8: Actual pIC_{50} vs Predicted pIC_{50} of the compounds from the CoMFA and CoMSIA training set compounds of SET-A.

#Cpd	Actual pIC_{50}	CoMFA		Predicted pIC_{50} (CoMSIA)					
		Predicted pIC_{50}	Residuals	SED	Residuals	SEAD	Residuals	SEHAD	Residuals
C59	6.55	6.896	-0.346	8.536	-0.571	6.877	-0.327	6.897	-0.347
C105	6.8	6.316	0.484	8.484	-0.754	7.241	-0.441	7.239	-0.439
C05	7.09	6.559	0.531	8.504	0.585	6.699	0.391	6.385	0.705
C27	7.19	7.478	-0.288	8.449	-0.628	7.81	-0.62	7.793	-0.603
C37	7.27	7.675	-0.405	8.529	-0.578	7.918	-0.648	7.657	-0.387
C45	7.45	6.999	0.451	8.781	0.613	6.79	0.66	6.858	0.592
C85	7.59	7.4	0.19	9.145	0.061	7.659	-0.069	7.643	-0.053
C198	7.75	8.52	-0.77	8.472	-0.711	8.553	-0.803	8.637	-0.887
C10	7.76	8.078	-0.318	7.868	-0.086	7.849	-0.089	7.953	-0.193
C56	7.79	8.251	-0.461	9.067	-0.152	7.918	-0.128	7.916	-0.126
C60	7.88	7.394	0.486	8.825	0.527	7.252	0.628	7.31	0.57
C196	7.99	8.443	-0.453	8.875	-0.525	8.627	-0.637	8.652	-0.662
C139	8	8.586	-0.586	8.295	-0.627	8.548	-0.548	8.573	-0.573
C16	8.04	8.027	0.013	8.845	-0.207	8.214	-0.174	8.212	-0.172
C36	8.09	7.591	0.499	8.251	-0.045	8.18	-0.09	8.36	-0.27
C92	8.1	8.523	-0.423	8.468	-0.358	8.492	-0.392	8.475	-0.375
C79	8.16	7.827	0.333	8.361	0.397	7.719	0.441	7.887	0.273
C55	8.23	8.179	0.051	7.923	0.114	8.076	0.154	8.148	0.082
C94	8.39	8.554	-0.164	8.845	-0.146	8.732	-0.342	8.668	-0.278
C01	8.44	8.388	0.052	8.224	-0.044	8.341	0.099	8.379	0.061
C209	8.46	8.704	-0.244	8.553	-0.044	8.431	0.029	8.549	-0.089
C53	8.48	8.29	0.19	8.957	0.031	8.418	0.062	8.397	0.083
C204	8.49	8.553	-0.063	9.127	-0.039	8.651	-0.161	8.566	-0.076
C214	8.5	8.759	-0.259	8.638	-0.281	8.693	-0.193	8.824	-0.324
C135	8.5	8.905	-0.405	8.756	-0.645	9.081	-0.581	9.063	-0.563
C180	8.52	8.326	0.194	8.55	0.048	8.526	-0.006	8.654	-0.134
C14	8.53	8.04	0.49	9.037	0.662	7.877	0.653	7.952	0.578
C99	8.55	8.788	-0.238	8.955	-0.517	8.939	-0.389	8.948	-0.398
C213	8.55	8.826	-0.276	8.97	-0.275	8.806	-0.256	8.966	-0.416
C194	8.56	8.574	-0.014	8.928	-0.315	8.932	-0.372	8.828	-0.268
C100	8.58	7.972	0.608	8.647	0.285	8.41	0.17	8.386	0.194
C76	8.58	8.154	0.426	8.981	-0.265	8.694	-0.114	8.618	-0.038
C89	8.61	8.408	0.202	8.757	0.359	8.149	0.461	8.354	0.256
C91	8.61	8.502	0.108	8.384	0.142	8.302	0.308	8.493	0.117

C169	8.63	8.428	0.202	8.524	0.269	8.322	0.308	8.368	0.262
C30	8.67	7.57	1.1	8.957	0.747	8.168	0.502	8	0.67
C128	8.7	8.861	-0.161	8.536	-0.145	8.851	-0.151	8.751	-0.051
C170	8.74	8.551	0.189	8.484	0.516	8.444	0.296	8.5	0.24
C191	8.76	8.7	0.06	8.504	0.207	8.712	0.048	8.579	0.181
C153	8.9	9.113	-0.213	8.449	-0.057	8.818	0.082	8.86	0.04
C136	8.9	9.122	-0.222	8.529	-0.227	9.064	-0.164	9.003	-0.103
C111	8.9	8.636	0.264	8.781	0.262	8.473	0.427	8.584	0.316
C68	8.92	8.456	0.464	9.145	0.164	8.689	0.231	8.649	0.271
C38	9	8.907	0.093	8.472	0.45	8.528	0.472	8.528	0.472
C160	9	8.763	0.237	7.868	-0.037	8.963	0.037	9.051	-0.051
C162	9	8.912	0.088	9.067	0.045	8.922	0.078	8.855	0.145
C122	9	9.148	-0.148	8.825	0.03	9.082	-0.082	9.075	-0.075
C132	9	8.63	0.37	8.875	0.072	8.988	0.012	8.881	0.119
C133	9	8.579	0.421	8.295	0.353	8.707	0.293	8.667	0.333
C131	9	8.754	0.246	8.845	0.019	9.009	-0.009	8.904	0.096
C211	9.04	8.556	0.484	8.251	0.283	8.922	0.118	8.83	0.21
C200	9.09	8.537	0.553	8.468	0.706	8.223	0.867	8.095	0.995
C113	9.2	8.73	0.47	8.361	0.676	8.371	0.829	8.511	0.689
C146	9.2	9.123	0.077	7.923	0.243	9.016	0.184	8.994	0.206
C21	5.27	5.72	-0.45	6.252	-0.982	6.238	-0.968	6.187	-0.917
C42	5.79	5.904	-0.114	6.472	-0.682	6.731	-0.941	6.533	-0.743
C40	6.22	6.137	0.083	6.428	-0.208	6.364	-0.144	6.216	0.004
C41	6.27	6.487	-0.217	6.814	-0.544	6.81	-0.54	6.649	-0.379
C44	6.3	6.7	-0.4	6.593	-0.293	6.718	-0.418	6.569	-0.269
C03	6.31	7.005	-0.695	6.438	-0.128	6.526	-0.216	6.527	-0.217
C15	6.36	6.368	-0.008	7.227	-0.867	7.369	-1.009	7.321	-0.961
C08	6.37	7.023	-0.653	6.826	-0.456	6.967	-0.597	6.964	-0.594
C06	6.53	6.602	-0.072	6.647	-0.117	6.602	-0.072	6.643	-0.113
C75	6.74	7.54	-0.8	7.716	-0.976	7.139	-0.399	7.18	-0.44
C20	7.03	6.987	0.043	6.897	0.133	7.033	-0.003	6.91	0.12
C43	7.07	6.929	0.141	6.59	0.48	6.619	0.451	6.519	0.551
C46	7.09	7.347	-0.257	6.942	0.148	7.013	0.077	7.054	0.036
C07	7.11	6.53	0.58	6.463	0.647	6.464	0.646	6.431	0.679
C19	7.15	6.707	0.443	6.731	0.419	6.919	0.231	6.823	0.327
C115	7.2	8.103	-0.903	7.925	-0.725	8.031	-0.831	8.013	-0.813
C12	7.22	7.114	0.106	6.966	0.254	7.129	0.091	7.149	0.071
C04	7.3	7.427	-0.127	7.367	-0.067	7.343	-0.043	7.323	-0.023
C81	7.3	7.447	-0.147	7.455	-0.155	7.54	-0.24	7.401	-0.101
C47	7.3	7.28	0.02	7.168	0.132	7.249	0.051	7.329	-0.029
C25	7.35	7.814	-0.464	7.537	-0.187	7.687	-0.337	7.683	-0.333
C54	7.37	8.05	-0.68	8.024	-0.654	7.921	-0.551	7.932	-0.562
C84	7.39	7.66	-0.27	7.803	-0.413	7.85	-0.46	7.796	-0.406
C165	7.46	8.357	-0.897	8.095	-0.635	8.221	-0.761	8.251	-0.791
C167	7.46	8.362	-0.902	8.484	-1.024	8.549	-1.089	8.609	-1.149
C52	7.48	7.324	0.156	7.581	-0.101	7.288	0.192	7.269	0.211

C26	7.5	7.459	0.041	7.494	0.006	7.34	0.16	7.362	0.138
C83	7.55	7.885	-0.335	7.825	-0.275	7.87	-0.32	7.943	-0.393
C82	7.58	7.693	-0.113	7.653	-0.073	7.749	-0.169	7.696	-0.116
C108	7.6	7.675	-0.075	7.469	0.131	7.645	-0.045	7.575	0.025
C11	7.82	8.012	-0.192	8.228	-0.408	8.187	-0.367	8.212	-0.392
C18	7.85	7.149	0.701	6.952	0.898	6.846	1.004	6.832	1.018
C61	7.92	7.885	0.035	7.623	0.297	7.469	0.451	7.558	0.362
C107	8	7.34	0.66	7.795	0.205	7.766	0.234	7.822	0.178
C80	8.04	7.836	0.204	7.865	0.175	7.91	0.13	7.909	0.131
C50	8.04	8.308	-0.268	8.702	-0.662	8.624	-0.584	8.702	-0.662
C202	8.07	8.634	-0.564	8.601	-0.531	8.355	-0.285	8.309	-0.239
C48	8.09	7.791	0.299	8.251	-0.161	7.976	0.114	8.028	0.062
C138	8.1	8.664	-0.564	8.806	-0.706	8.637	-0.537	8.816	-0.716
C110	8.1	8.008	0.092	8.359	-0.259	8.281	-0.181	8.306	-0.206
C28	8.12	7.849	0.271	7.695	0.425	7.875	0.245	7.837	0.283
C17	8.13	8.372	-0.242	8.484	-0.354	8.352	-0.222	8.347	-0.217
C51	8.15	7.735	0.415	8.01	0.14	7.891	0.259	7.914	0.236
C35	8.18	8.605	-0.425	8.348	-0.168	8.109	0.071	8.2	-0.02
C23	8.19	8.221	-0.031	8.029	0.161	8.124	0.066	8.153	0.037
C109	8.2	7.839	0.361	7.805	0.395	7.965	0.235	7.962	0.238
C24	8.22	8.136	0.084	7.911	0.309	7.857	0.363	7.706	0.514
C78	8.22	7.783	0.437	7.787	0.433	7.835	0.385	7.925	0.295
C197	8.25	8.157	0.093	8.219	0.031	8.424	-0.174	8.317	-0.067
C09	8.25	8.166	0.084	8.454	-0.204	8.311	-0.061	8.303	-0.053
C77	8.3	8.33	-0.03	8.423	-0.123	8.612	-0.312	8.543	-0.243
C74	8.3	8.429	-0.129	8.258	0.042	8.351	-0.051	8.374	-0.074
C190	8.31	8.604	-0.294	8.537	-0.227	8.671	-0.361	8.527	-0.217
C181	8.31	8.418	-0.108	8.42	-0.11	8.506	-0.196	8.681	-0.371
C184	8.32	8.661	-0.341	8.414	-0.094	8.304	0.016	8.238	0.082
C185	8.33	8.604	-0.274	8.428	-0.098	8.312	0.018	8.241	0.089
C62	8.34	7.967	0.373	7.752	0.588	7.681	0.659	7.855	0.485
C13	8.35	8.201	0.149	8.462	-0.112	8.329	0.021	8.329	0.021
C88	8.36	8.514	-0.154	8.411	-0.051	8.168	0.192	8.259	0.101
C193	8.37	8.68	-0.31	8.566	-0.196	8.697	-0.327	8.524	-0.154
C87	8.37	8.313	0.057	8.176	0.194	8.019	0.351	8.082	0.288
C178	8.37	8.597	-0.227	8.625	-0.255	8.726	-0.356	8.723	-0.353
C72	8.39	8.559	-0.169	8.487	-0.097	8.593	-0.203	8.541	-0.151
C96	8.39	8.544	-0.154	8.508	-0.118	8.505	-0.115	8.461	-0.071
C182	8.42	8.672	-0.252	8.445	-0.025	8.357	0.063	8.319	0.101
C90	8.43	8.594	-0.164	8.351	0.079	8.346	0.084	8.472	-0.042
C104	8.44	8.525	-0.085	8.734	-0.294	8.747	-0.307	8.784	-0.344
C168	8.44	8.25	0.19	8.233	0.207	8.23	0.21	8.339	0.101
C186	8.44	8.766	-0.326	8.723	-0.283	8.603	-0.163	8.703	-0.263
C188	8.44	8.722	-0.282	8.645	-0.205	8.524	-0.084	8.645	-0.205
C203	8.45	8.57	-0.12	8.494	-0.044	8.398	0.052	8.353	0.097
C175	8.45	8.522	-0.072	8.494	-0.044	8.722	-0.272	8.787	-0.337

C166	8.46	8.382	0.078	7.912	0.548	7.984	0.476	8.083	0.377
C49	8.46	8.236	0.224	8.454	0.006	8.453	0.007	8.363	0.097
C58	8.48	8.002	0.478	7.924	0.556	7.932	0.548	8.026	0.454
C201	8.48	8.589	-0.109	8.532	-0.052	8.311	0.169	8.24	0.24
C101	8.48	8.313	0.167	8.379	0.101	8.477	0.003	8.458	0.022
C86	8.48	8.151	0.329	8.105	0.375	8.021	0.459	8.158	0.322
C103	8.48	8.163	0.317	8.372	0.108	8.466	0.014	8.449	0.031
C69	8.5	8.523	-0.023	8.439	0.061	8.543	-0.043	8.485	0.015
C207	8.5	8.719	-0.219	8.428	0.072	8.482	0.018	8.629	-0.129
C192	8.52	8.662	-0.142	8.574	-0.054	8.701	-0.181	8.545	-0.025
C176	8.53	8.311	0.219	8.487	0.043	8.7	-0.17	8.811	-0.281
C22	8.53	8.765	-0.235	8.79	-0.26	8.72	-0.19	8.744	-0.214
C64	8.55	8.539	0.011	8.609	-0.059	8.687	-0.137	8.634	-0.084
C215	8.55	8.597	-0.047	8.594	-0.044	8.614	-0.064	8.726	-0.176
C73	8.56	8.374	0.186	8.287	0.273	8.37	0.19	8.368	0.192
C195	8.56	8.661	-0.101	8.586	-0.026	8.369	0.191	8.289	0.271
C33	8.56	8.45	0.11	8.781	-0.221	8.642	-0.082	8.528	0.032
C57	8.58	8.393	0.187	7.877	0.703	7.725	0.855	7.853	0.727
C95	8.58	8.626	-0.046	8.61	-0.03	8.719	-0.139	8.688	-0.108
C208	8.6	8.597	0.003	8.33	0.27	8.337	0.263	8.343	0.257
C129	8.6	8.502	0.098	8.758	-0.158	8.797	-0.197	8.655	-0.055
C140	8.6	8.512	0.088	8.515	0.085	8.371	0.229	8.704	-0.104
C130	8.6	8.454	0.146	8.884	-0.284	8.937	-0.337	8.804	-0.204
C63	8.6	8.235	0.365	8.561	0.039	8.639	-0.039	8.589	0.011
C93	8.6	8.152	0.448	8.64	-0.04	8.472	0.128	8.46	0.14
C183	8.61	8.676	-0.066	8.509	0.101	8.451	0.159	8.568	0.042
C189	8.61	8.693	-0.083	8.498	0.112	8.669	-0.059	8.534	0.076
C67	8.65	8.427	0.223	8.705	-0.055	8.658	-0.008	8.613	0.037
C31	8.65	8.876	-0.226	8.805	-0.155	8.594	0.056	8.623	0.027
C29	8.65	8.425	0.225	8.172	0.478	8.266	0.384	8.243	0.407
C39	8.67	8.807	-0.137	8.881	-0.211	8.845	-0.175	8.776	-0.106
C212	8.67	8.507	0.163	8.872	-0.202	8.99	-0.32	8.86	-0.19
C173	8.67	8.421	0.249	8.44	0.23	8.403	0.267	8.528	0.142
C102	8.67	8.265	0.405	8.409	0.261	8.491	0.179	8.471	0.199
C65	8.67	8.589	0.081	8.689	-0.019	8.748	-0.078	8.683	-0.013
C174	8.69	8.484	0.206	8.513	0.177	8.37	0.32	8.505	0.185
C34	8.72	8.389	0.331	8.247	0.473	8.174	0.546	8.301	0.419
C177	8.72	8.528	0.192	8.493	0.227	8.598	0.122	8.636	0.084
C171	8.74	8.476	0.264	8.322	0.418	8.56	0.18	8.639	0.101
C172	8.74	8.649	0.091	8.639	0.101	8.681	0.059	8.697	0.043
C187	8.74	8.862	-0.122	8.778	-0.038	8.617	0.123	8.681	0.059
C199	8.76	8.581	0.179	8.591	0.169	8.353	0.407	8.328	0.432
C70	8.78	8.655	0.125	8.499	0.281	8.553	0.227	8.452	0.328
C66	8.79	8.608	0.182	8.66	0.13	8.719	0.071	8.677	0.113
C137	8.8	8.966	-0.166	9.063	-0.263	9.092	-0.292	9.039	-0.239
C142	8.8	8.586	0.214	8.794	0.006	8.561	0.239	8.86	-0.06

C97	8.82	8.57	0.25	8.622	0.198	8.791	0.029	8.791	0.029
C71	8.82	8.62	0.2	8.517	0.303	8.618	0.202	8.618	0.202
C32	8.88	8.979	-0.099	8.837	0.043	8.987	-0.107	8.87	0.01
C205	8.88	8.699	0.181	8.759	0.121	8.99	-0.11	8.894	-0.014
C118	8.9	9.153	-0.253	9.26	-0.36	9.231	-0.331	9.243	-0.343
C141	8.9	8.574	0.326	8.575	0.325	8.396	0.504	8.709	0.191
C161	8.9	8.999	-0.099	9.054	-0.154	8.929	-0.029	8.932	-0.032
C151	8.9	8.844	0.056	8.965	-0.065	8.976	-0.076	9.027	-0.127
C127	8.9	8.803	0.097	8.854	0.046	8.808	0.092	8.712	0.188
C124	8.9	8.838	0.062	8.855	0.045	8.813	0.087	8.718	0.182
C163	8.9	9.054	-0.154	8.833	0.067	9.069	-0.169	9.008	-0.108
C114	8.9	8.903	-0.003	8.421	0.479	8.327	0.573	8.362	0.538
C120	8.9	9.094	-0.194	8.772	0.128	8.91	-0.01	8.894	0.006
C179	8.92	8.548	0.372	8.4	0.52	8.362	0.558	8.498	0.422
C98	8.92	8.906	0.014	8.97	-0.05	8.968	-0.048	8.97	-0.05
C145	9	9.062	-0.062	9.055	-0.055	8.912	0.088	8.897	0.103
C155	9	9.116	-0.116	8.908	0.092	8.995	0.005	8.949	0.051
C154	9	9.113	-0.113	9.049	-0.049	9.009	-0.009	9.015	-0.015
C152	9	8.739	0.261	8.893	0.107	8.8	0.2	8.883	0.117
C144	9	8.886	0.114	9.061	-0.061	9.097	-0.097	9.02	-0.02
C157	9	9.028	-0.028	8.952	0.048	9.126	-0.126	9.05	-0.05
C123	9	8.951	0.049	9.034	-0.034	9.077	-0.077	8.965	0.035
C164	9	9.088	-0.088	8.963	0.037	9.109	-0.109	9.046	-0.046
C156	9	9.119	-0.119	9.049	-0.049	9.127	-0.127	9.119	-0.119
C147	9	9.04	-0.04	9.125	-0.125	9.095	-0.095	9.096	-0.096
C206	9.04	8.443	0.597	8.633	0.407	8.607	0.433	8.613	0.427
C210	9.09	8.705	0.385	8.803	0.287	8.68	0.41	8.649	0.441
C148	9.1	9.077	0.023	9.017	0.083	9.09	0.01	9.064	0.036
C134	9.1	8.983	0.117	9.242	-0.142	9.113	-0.013	9.146	-0.046
C149	9.1	8.987	0.113	8.92	0.18	9.09	0.01	8.997	0.103
C150	9.1	9.029	0.071	9.02	0.08	9.098	0.002	9.09	0.01
C112	9.1	8.979	0.121	8.673	0.427	8.501	0.599	8.712	0.388
C143	9.1	8.621	0.479	8.588	0.512	8.515	0.585	8.825	0.275
C117	9.1	9.138	-0.038	9.211	-0.111	9.135	-0.035	9.129	-0.029
C125	9.1	8.935	0.165	8.878	0.222	8.886	0.214	8.777	0.323
C159	9.1	9.028	0.072	8.95	0.15	9.086	0.014	9.029	0.071
C126	9.1	8.882	0.218	9.003	0.097	9.026	0.074	8.926	0.174
C158	9.1	9.116	-0.016	9.049	0.051	9.093	0.007	9.106	-0.006
C119	9.1	8.956	0.144	8.663	0.437	8.731	0.369	8.686	0.414
C116	9.2	8.997	0.203	9.493	-0.293	9.227	-0.027	9.25	-0.05
C121	9.2	9.103	0.097	8.917	0.283	8.853	0.347	8.817	0.383

#Cpd: Compounds

Table S9: Actual pIC_{50} vs Predicted pIC_{50} of the compounds from the CoMFA and CoMSIA training set compounds of SET-B.

#Cpd		CoMFA	Predicted pIC_{50} (CoMSIA)

	Actual pIC_{50}	Predicted pIC_{50}	Residuals	SD	Residuals	SED	Residuals	SEAD	Residuals
C44	6.3	6.923	-0.623	7.132	-0.832	7.047	-0.747	7.049	-0.749
C03	6.31	7.974	-1.664	6.501	-0.191	6.876	-0.566	6.645	-0.335
C59	6.55	7.982	-1.432	8.168	-1.618	8.11	-1.56	8.203	-1.653
C75	6.74	8.057	-1.317	8.145	-1.405	8.155	-1.415	8.177	-1.437
C20	7.03	7.21	-0.18	6.888	0.142	7.121	-0.091	7.033	-0.003
C46	7.09	7.388	-0.298	7.16	-0.07	7.084	0.006	7.391	-0.301
C12	7.22	6.979	0.241	7.492	-0.272	7.27	-0.05	7.61	-0.39
C54	7.37	8.247	-0.877	8.067	-0.697	8.107	-0.737	8.105	-0.735
C26	7.5	7.24	0.26	7.564	-0.064	7.554	-0.054	7.556	-0.056
C82	7.58	7.775	-0.195	7.672	-0.092	7.633	-0.053	7.508	0.072
C18	7.85	7.202	0.648	6.766	1.084	7.027	0.823	6.857	0.993
C202	8.07	8.932	-0.862	8.89	-0.82	8.745	-0.675	8.863	-0.793
C138	8.1	9.031	-0.931	9.015	-0.915	8.674	-0.574	8.976	-0.876
C51	8.15	7.871	0.279	7.936	0.214	8.019	0.131	7.801	0.349
C23	8.19	7.916	0.274	7.885	0.305	7.768	0.422	7.795	0.395
C09	8.25	7.867	0.383	8.082	0.168	7.85	0.4	7.965	0.285
C77	8.3	8.485	-0.185	8.685	-0.385	8.491	-0.191	8.54	-0.24
C181	8.31	8.373	-0.063	8.545	-0.235	8.392	-0.082	8.624	-0.314
C184	8.32	8.624	-0.304	8.407	-0.087	8.428	-0.108	8.218	0.102
C94	8.39	8.356	0.034	7.927	0.463	8.422	-0.032	8.185	0.205
C182	8.42	8.608	-0.188	8.351	0.069	8.46	-0.04	8.365	0.055
C90	8.43	8.508	-0.078	8.017	0.413	8.25	0.18	8.152	0.278
C168	8.44	8.305	0.135	8.206	0.234	8.242	0.198	8.336	0.104
C58	8.48	7.938	0.542	7.925	0.555	7.9	0.58	7.901	0.579
C86	8.48	8.199	0.281	8.027	0.453	8.065	0.415	8.065	0.415
C176	8.53	8.199	0.331	8.379	0.151	8.365	0.165	8.565	-0.035
C64	8.55	8.596	-0.046	8.741	-0.191	8.724	-0.174	8.667	-0.117
C73	8.56	8.291	0.269	8.35	0.21	8.238	0.322	8.29	0.27
C33	8.56	7.966	0.594	8.623	-0.063	7.282	1.278	8.464	0.096
C57	8.58	8.386	0.194	8.168	0.412	7.837	0.743	8.28	0.3
C208	8.6	8.475	0.125	8.361	0.239	8.277	0.323	8.329	0.271
C67	8.65	8.613	0.037	8.816	-0.166	8.816	-0.166	8.777	-0.127
C31	8.65	8.555	0.095	8.063	0.587	8.117	0.533	7.976	0.674
C29	8.65	8.39	0.26	8.743	-0.093	8.337	0.313	8.876	-0.226
C212	8.67	8.59	0.08	8.847	-0.177	9.214	-0.544	8.922	-0.252
C102	8.67	8.358	0.312	8.622	0.048	8.44	0.23	8.574	0.096
C172	8.74	8.469	0.271	8.509	0.231	8.504	0.236	8.405	0.335
C187	8.74	8.682	0.058	8.438	0.302	8.761	-0.021	8.488	0.252
C97	8.82	8.384	0.436	8.107	0.713	8.579	0.241	8.266	0.554
C32	8.88	8.744	0.136	8.135	0.745	8.24	0.64	8.054	0.826
C205	8.88	8.417	0.463	8.497	0.383	8.726	0.154	8.624	0.256
C151	8.9	9.046	-0.146	9.16	-0.26	8.963	-0.063	9.022	-0.122
C127	8.9	8.924	-0.024	8.784	0.116	8.832	0.068	8.702	0.198
C120	8.9	8.951	-0.051	8.525	0.375	8.491	0.409	8.732	0.168

C179	8.92	8.43	0.49	8.338	0.582	8.407	0.513	8.426	0.494
C145	9	8.92	0.08	9.185	-0.185	9.254	-0.254	9.271	-0.271
C154	9	8.982	0.018	9.209	-0.209	9.192	-0.192	9.186	-0.186
C144	9	8.88	0.12	9.132	-0.132	9.247	-0.247	9.207	-0.207
C156	9	9.153	-0.153	9.214	-0.214	9.245	-0.245	9.187	-0.187
C149	9.1	8.919	0.181	8.807	0.293	8.934	0.166	8.86	0.24
C117	9.1	8.971	0.129	9.011	0.089	8.943	0.157	9.144	-0.044
C159	9.1	9.01	0.09	8.826	0.274	9.031	0.069	8.887	0.213
C119	9.1	8.894	0.206	8.476	0.624	8.471	0.629	8.638	0.462
C113	9.2	8.785	0.415	8.447	0.753	8.435	0.765	8.618	0.582
C21	5.27	5.991	-0.721	6.543	-1.273	6.552	-1.282	6.599	-1.329
C42	5.79	6.193	-0.403	6.665	-0.875	6.606	-0.816	6.505	-0.715
C40	6.22	6.14	0.08	6.742	-0.522	6.531	-0.311	6.481	-0.261
C41	6.27	6.459	-0.189	6.775	-0.505	6.84	-0.57	6.538	-0.268
C15	6.36	6.509	-0.149	7.313	-0.953	7.407	-1.047	7.361	-1.001
C08	6.37	6.691	-0.321	6.748	-0.378	6.673	-0.303	6.74	-0.37
C06	6.53	6.457	0.073	6.697	-0.167	6.509	0.021	6.681	-0.151
C105	6.8	7.738	-0.938	7.266	-0.466	7.436	-0.636	7.378	-0.578
C43	7.07	7.064	0.006	6.844	0.226	6.706	0.364	6.738	0.332
C05	7.09	6.932	0.158	6.653	0.437	6.695	0.395	6.648	0.442
C07	7.11	6.829	0.281	6.652	0.458	6.586	0.524	6.651	0.459
C19	7.15	6.872	0.278	6.721	0.429	6.93	0.22	6.782	0.368
C27	7.19	7.579	-0.389	7.534	-0.344	7.745	-0.555	7.664	-0.474
C115	7.2	8.052	-0.852	7.623	-0.423	7.852	-0.652	7.728	-0.528
C37	7.27	7.407	-0.137	7.768	-0.498	7.864	-0.594	7.767	-0.497
C04	7.3	7.637	-0.337	7.433	-0.133	7.702	-0.402	7.554	-0.254
C81	7.3	7.474	-0.174	7.515	-0.215	7.356	-0.056	7.205	0.095
C47	7.3	7.386	-0.086	7.169	0.131	7.223	0.077	7.21	0.09
C25	7.35	7.585	-0.235	7.282	0.068	7.394	-0.044	7.365	-0.015
C84	7.39	7.783	-0.393	7.902	-0.512	7.812	-0.422	7.705	-0.315
C45	7.45	7.147	0.303	7.158	0.292	6.977	0.473	7.245	0.205
C165	7.46	8.182	-0.722	8.31	-0.85	8.065	-0.605	8.177	-0.717
C167	7.46	8.275	-0.815	8.581	-1.121	8.451	-0.991	8.55	-1.09
C52	7.48	7.403	0.077	7.933	-0.453	7.706	-0.226	7.77	-0.29
C83	7.55	7.973	-0.423	7.904	-0.354	7.805	-0.255	7.888	-0.338
C85	7.59	7.35	0.24	7.527	0.063	7.406	0.184	7.445	0.145
C108	7.6	7.542	0.058	7.326	0.274	7.301	0.299	7.298	0.302
C198	7.75	8.392	-0.642	8.355	-0.605	8.356	-0.606	8.402	-0.652
C10	7.76	8.152	-0.392	8.373	-0.613	7.989	-0.229	8.39	-0.63
C56	7.79	8.325	-0.535	8.082	-0.292	7.918	-0.128	8.069	-0.279
C11	7.82	7.871	-0.051	8.147	-0.327	7.9	-0.08	8.019	-0.199
C60	7.88	7.538	0.342	7.511	0.369	7.428	0.452	7.493	0.387
C61	7.92	7.931	-0.011	8.138	-0.218	7.716	0.204	8.101	-0.181
C196	7.99	8.316	-0.326	8.35	-0.36	8.405	-0.415	8.297	-0.307
C107	8	7.256	0.744	8.02	-0.02	7.621	0.379	8.024	-0.024
C139	8	8.691	-0.691	8.743	-0.743	8.39	-0.39	8.721	-0.721

C80	8.04	7.927	0.113	7.786	0.254	7.875	0.165	7.701	0.339
C16	8.04	7.899	0.141	8.148	-0.108	7.982	0.058	8.094	-0.054
C50	8.04	8.208	-0.168	8.489	-0.449	8.798	-0.758	8.425	-0.385
C36	8.09	7.717	0.373	7.746	0.344	8.011	0.079	8.103	-0.013
C48	8.09	7.779	0.311	8.489	-0.399	8.148	-0.058	8.433	-0.343
C92	8.1	8.35	-0.25	7.874	0.226	8.305	-0.205	8.045	0.055
C110	8.1	8.082	0.018	8.342	-0.242	8.161	-0.061	8.549	-0.449
C28	8.12	7.619	0.501	7.462	0.658	7.548	0.572	7.503	0.617
C17	8.13	8.486	-0.356	8.094	0.036	7.941	0.189	8.057	0.073
C79	8.16	7.701	0.459	7.823	0.337	7.727	0.433	7.841	0.319
C35	8.18	8.46	-0.28	8.477	-0.297	8.433	-0.253	8.479	-0.299
C109	8.2	7.718	0.482	7.285	0.915	7.653	0.547	7.296	0.904
C24	8.22	7.918	0.302	7.551	0.669	7.766	0.454	7.392	0.828
C78	8.22	7.844	0.376	7.858	0.362	7.756	0.464	7.889	0.331
C55	8.23	8.146	0.084	8.324	-0.094	8.141	0.089	8.264	-0.034
C197	8.25	7.987	0.263	7.973	0.277	8.108	0.142	7.798	0.452
C74	8.3	8.447	-0.147	8.421	-0.121	8.205	0.095	8.39	-0.09
C190	8.31	8.616	-0.306	8.457	-0.147	8.438	-0.128	8.421	-0.111
C185	8.33	8.561	-0.231	8.453	-0.123	8.442	-0.112	8.187	0.143
C62	8.34	7.994	0.346	8.22	0.12	7.799	0.541	8.277	0.063
C13	8.35	7.999	0.351	8.083	0.267	7.929	0.421	8.018	0.332
C88	8.36	8.433	-0.073	8.103	0.257	8.463	-0.103	8.219	0.141
C193	8.37	8.704	-0.334	8.466	-0.096	8.49	-0.12	8.379	-0.009
C87	8.37	8.345	0.025	8.163	0.207	8.137	0.233	7.996	0.374
C178	8.37	8.468	-0.098	8.678	-0.308	8.653	-0.283	8.589	-0.219
C72	8.39	8.754	-0.364	8.72	-0.33	8.591	-0.201	8.552	-0.162
C96	8.39	8.614	-0.224	8.347	0.043	8.609	-0.219	8.335	0.055
C104	8.44	8.558	-0.118	8.584	-0.144	8.638	-0.198	8.535	-0.095
C186	8.44	8.595	-0.155	8.383	0.057	8.598	-0.158	8.425	0.015
C01	8.44	8.601	-0.161	8.333	0.107	8.411	0.029	8.514	-0.074
C188	8.44	8.681	-0.241	8.443	-0.003	8.598	-0.158	8.539	-0.099
C203	8.45	8.679	-0.229	8.723	-0.273	8.577	-0.127	8.681	-0.231
C175	8.45	8.34	0.11	8.382	0.068	8.446	0.004	8.474	-0.024
C209	8.46	8.575	-0.115	8.302	0.158	8.468	-0.008	8.466	-0.006
C166	8.46	8.251	0.209	8.096	0.364	7.9	0.56	8.064	0.396
C49	8.46	8.125	0.335	8.175	0.285	8.526	-0.066	7.919	0.541
C53	8.48	8.335	0.145	8.587	-0.107	8.522	-0.042	8.321	0.159
C201	8.48	8.825	-0.345	8.784	-0.304	8.665	-0.185	8.714	-0.234
C101	8.48	8.522	-0.042	8.563	-0.083	8.481	-0.001	8.528	-0.048
C103	8.48	8.372	0.108	8.572	-0.092	8.41	0.07	8.509	-0.029
C204	8.49	8.545	-0.055	8.431	0.059	8.711	-0.221	8.467	0.023
C69	8.5	8.575	-0.075	8.59	-0.09	8.508	-0.008	8.522	-0.022
C214	8.5	8.77	-0.27	8.523	-0.023	8.758	-0.258	8.72	-0.22
C135	8.5	8.66	-0.16	8.666	-0.166	8.799	-0.299	8.852	-0.352
C207	8.5	8.549	-0.049	8.264	0.236	8.376	0.124	8.483	0.017
C180	8.52	8.314	0.206	8.584	-0.064	8.539	-0.019	8.685	-0.165

C192	8.52	8.714	-0.194	8.481	0.039	8.506	0.014	8.405	0.115
C14	8.53	8.099	0.431	8.374	0.156	8.072	0.458	8.463	0.067
C22	8.53	8.952	-0.422	8.477	0.053	8.491	0.039	8.626	-0.096
C99	8.55	8.795	-0.245	8.828	-0.278	9.044	-0.494	8.802	-0.252
C215	8.55	8.625	-0.075	8.551	-0.001	8.56	-0.01	8.631	-0.081
C213	8.55	8.752	-0.202	8.53	0.02	8.708	-0.158	8.732	-0.182
C194	8.56	8.512	0.048	8.43	0.13	8.743	-0.183	8.464	0.096
C195	8.56	8.835	-0.275	8.834	-0.274	8.761	-0.201	8.76	-0.2
C100	8.58	8.338	0.242	8.414	0.166	8.398	0.182	8.352	0.228
C76	8.58	8.389	0.191	9.009	-0.429	8.898	-0.318	8.733	-0.153
C95	8.58	8.492	0.088	8.247	0.333	8.587	-0.007	8.418	0.162
C129	8.6	8.668	-0.068	8.735	-0.135	8.777	-0.177	8.626	-0.026
C140	8.6	8.519	0.081	8.914	-0.314	8.414	0.186	8.953	-0.353
C130	8.6	8.623	-0.023	8.878	-0.278	8.89	-0.29	8.796	-0.196
C63	8.6	8.436	0.164	8.597	0.003	8.66	-0.06	8.508	0.092
C93	8.6	8.266	0.334	8.612	-0.012	8.485	0.115	8.518	0.082
C183	8.61	8.61	0	8.338	0.272	8.515	0.095	8.51	0.1
C89	8.61	8.417	0.193	7.995	0.615	8.208	0.402	8.177	0.433
C189	8.61	8.601	0.009	8.402	0.208	8.398	0.212	8.425	0.185
C91	8.61	8.37	0.24	8.039	0.571	8.294	0.316	8.09	0.52
C169	8.63	8.443	0.187	8.371	0.259	8.371	0.259	8.401	0.229
C39	8.67	8.553	0.117	8.827	-0.157	8.672	-0.002	8.763	-0.093
C30	8.67	8.7	-0.03	8.693	-0.023	8.728	-0.058	8.658	0.012
C173	8.67	8.41	0.26	8.373	0.297	8.529	0.141	8.489	0.181
C65	8.67	8.781	-0.111	8.873	-0.203	8.805	-0.135	8.783	-0.113
C174	8.69	8.394	0.296	8.536	0.154	8.493	0.197	8.573	0.117
C128	8.7	8.906	-0.206	8.761	-0.061	8.803	-0.103	8.702	-0.002
C34	8.72	8.261	0.459	8.386	0.334	8.317	0.403	8.488	0.232
C177	8.72	8.426	0.294	8.358	0.362	8.399	0.321	8.344	0.376
C171	8.74	8.437	0.303	8.407	0.333	8.31	0.43	8.408	0.332
C170	8.74	8.415	0.325	8.274	0.466	8.238	0.502	8.324	0.416
C191	8.76	8.704	0.056	8.444	0.316	8.482	0.278	8.439	0.321
C199	8.76	8.777	-0.017	8.848	-0.088	8.721	0.039	8.835	-0.075
C70	8.78	8.637	0.143	8.569	0.211	8.61	0.17	8.519	0.261
C66	8.79	8.749	0.041	8.814	-0.024	8.762	0.028	8.702	0.088
C137	8.8	8.923	-0.123	8.713	0.087	9.009	-0.209	8.805	-0.005
C142	8.8	8.694	0.106	8.822	-0.022	8.477	0.323	8.852	-0.052
C71	8.82	8.766	0.054	8.743	0.077	8.579	0.241	8.661	0.159
C118	8.9	9.082	-0.182	9.051	-0.151	8.929	-0.029	9.22	-0.32
C153	8.9	9.001	-0.101	9.187	-0.287	9.196	-0.296	9.086	-0.186
C141	8.9	8.597	0.303	9.041	-0.141	8.483	0.417	9.015	-0.115
C161	8.9	8.978	-0.078	9.166	-0.266	9.244	-0.344	9.16	-0.26
C136	8.9	8.909	-0.009	8.614	0.286	8.759	0.141	8.734	0.166
C124	8.9	8.934	-0.034	8.776	0.124	8.83	0.07	8.702	0.198
C111	8.9	8.766	0.134	8.331	0.569	8.562	0.338	8.655	0.245
C163	8.9	9.061	-0.161	8.7	0.2	8.978	-0.078	8.835	0.065

C114	8.9	8.847	0.053	8.097	0.803	8.305	0.595	8.199	0.701
C68	8.92	8.751	0.169	8.893	0.027	8.857	0.063	8.811	0.109
C98	8.92	8.787	0.133	8.753	0.167	8.952	-0.032	8.693	0.227
C38	9	8.842	0.158	8.945	0.055	8.744	0.256	9.02	-0.02
C155	9	8.979	0.021	8.832	0.168	8.94	0.06	8.853	0.147
C160	9	8.936	0.064	9.225	-0.225	9.065	-0.065	9.104	-0.104
C152	9	8.921	0.079	9.111	-0.111	9.051	-0.051	8.998	0.002
C162	9	8.877	0.123	8.803	0.197	9.01	-0.01	8.921	0.079
C122	9	9.219	-0.219	8.867	0.133	8.926	0.074	9.046	-0.046
C132	9	8.758	0.242	8.901	0.099	8.92	0.08	8.847	0.153
C133	9	8.697	0.303	9.178	-0.178	8.86	0.14	9.054	-0.054
C157	9	9.054	-0.054	8.861	0.139	9.019	-0.019	8.958	0.042
C123	9	9.02	-0.02	8.818	0.182	8.965	0.035	8.892	0.108
C164	9	9.057	-0.057	8.863	0.137	9.065	-0.065	8.948	0.052
C147	9	8.931	0.069	9.144	-0.144	9.081	-0.081	9.114	-0.114
C131	9	8.867	0.133	8.9	0.1	8.952	0.048	8.934	0.066
C211	9.04	8.65	0.39	8.846	0.194	9.047	-0.007	8.899	0.141
C206	9.04	8.468	0.572	8.372	0.668	8.479	0.561	8.46	0.58
C200	9.09	8.702	0.388	8.613	0.477	8.555	0.535	8.5	0.59
C210	9.09	8.885	0.205	8.938	0.152	8.873	0.217	8.971	0.119
C148	9.1	9.024	0.076	9.153	-0.053	9.156	-0.056	9.083	0.017
C134	9.1	8.753	0.347	9.042	0.058	9.065	0.035	9.036	0.064
C150	9.1	9.023	0.077	9.167	-0.067	9.182	-0.082	9.119	-0.019
C112	9.1	8.954	0.146	8.414	0.686	8.58	0.52	8.73	0.37
C143	9.1	8.677	0.423	8.937	0.163	8.478	0.622	8.902	0.198
C125	9.1	9.007	0.093	8.769	0.331	8.853	0.247	8.777	0.323
C126	9.1	8.954	0.146	8.91	0.19	8.964	0.136	8.944	0.156
C158	9.1	9.111	-0.011	9.189	-0.089	9.266	-0.166	9.126	-0.026
C146	9.2	9.212	-0.012	8.986	0.214	9.221	-0.021	8.943	0.257
C116	9.2	8.929	0.271	9.378	-0.178	9.141	0.059	9.393	-0.193
C121	9.2	9.166	0.034	8.764	0.436	8.992	0.208	8.896	0.304

#Cpd: Compounds

Table S10: Actual pIC_{50} vs. Predicted pIC_{50} of the compounds from the CoMFA and CoMSIA training set compounds of SET-C.

#Cpd	Actual pIC_{50}	CoMFA		Predicted pIC_{50} (CoMSIA)					
		Predicted pIC_{50}	Residuals	SD	Residuals	SED	Residuals	SEAD	Residuals
C41	6.27	6.709	-0.439	6.812	-0.542	7.103	-0.833	6.969	-0.699
C19	7.15	6.773	0.377	6.667	0.483	6.869	0.281	6.976	0.174
C81	7.3	7.236	0.064	7.462	-0.162	7.45	-0.15	7.529	-0.229
C47	7.3	7.361	-0.061	7.165	0.135	7.279	0.021	7.253	0.047
C165	7.46	8.581	-1.121	8.494	-1.034	8.229	-0.769	8.37	-0.91
C167	7.46	8.859	-1.399	8.96	-1.5	8.946	-1.486	8.966	-1.506
C52	7.48	7.272	0.208	7.948	-0.468	7.594	-0.114	7.226	0.254
C83	7.55	7.767	-0.217	7.944	-0.394	7.786	-0.236	7.848	-0.298

C108	7.6	7.703	-0.103	7.4	0.2	7.393	0.207	7.496	0.104
C11	7.82	8.145	-0.325	8.316	-0.496	8.099	-0.279	8.128	-0.308
C61	7.92	8.009	-0.089	8.239	-0.319	7.706	0.214	7.51	0.41
C80	8.04	7.644	0.396	7.733	0.307	7.796	0.244	7.858	0.182
C50	8.04	7.762	0.278	8.634	-0.594	8.004	0.036	8.327	-0.287
C48	8.09	7.685	0.405	8.634	-0.544	7.94	0.15	7.866	0.224
C28	8.12	7.62	0.5	7.494	0.626	7.446	0.674	7.637	0.483
C17	8.13	8.219	-0.089	8.436	-0.306	8.322	-0.192	8.137	-0.007
C35	8.18	8.704	-0.524	8.389	-0.209	8.344	-0.164	8.277	-0.097
C78	8.22	7.631	0.589	7.847	0.373	7.727	0.493	7.799	0.421
C62	8.34	8.022	0.318	8.395	-0.055	7.797	0.543	7.722	0.618
C13	8.35	8.068	0.282	8.444	-0.094	8.325	0.025	8.141	0.209
C193	8.37	8.792	-0.422	8.654	-0.284	8.618	-0.248	8.544	-0.174
C87	8.37	8.286	0.084	8.253	0.117	8.13	0.24	8.042	0.328
C72	8.39	8.646	-0.256	8.835	-0.445	8.59	-0.2	8.75	-0.36
C96	8.39	8.469	-0.079	8.119	0.271	8.315	0.075	8.175	0.215
C104	8.44	8.781	-0.341	8.624	-0.184	8.784	-0.344	8.825	-0.385
C166	8.46	8.563	-0.103	8.182	0.278	8.051	0.409	8.186	0.274
C49	8.46	7.409	1.051	8.185	0.275	7.701	0.759	8.046	0.414
C201	8.48	8.425	0.055	8.543	-0.063	8.337	0.143	8.272	0.208
C101	8.48	8.491	-0.011	8.578	-0.098	8.478	0.002	8.615	-0.135
C22	8.53	8.35	0.18	8.282	0.248	8.487	0.043	8.43	0.1
C215	8.55	8.926	-0.376	8.793	-0.243	8.94	-0.39	8.953	-0.403
C130	8.6	8.706	-0.106	9.094	-0.494	9	-0.4	8.944	-0.344
C93	8.6	8.054	0.546	8.613	-0.013	8.487	0.113	8.341	0.259
C183	8.61	8.634	-0.024	8.386	0.224	8.444	0.166	8.477	0.133
C189	8.61	8.77	-0.16	8.548	0.062	8.535	0.075	8.522	0.088
C39	8.67	8.914	-0.244	9.039	-0.369	8.795	-0.125	8.822	-0.152
C65	8.67	8.599	0.071	8.871	-0.201	8.738	-0.068	8.813	-0.143
C34	8.72	8.466	0.254	8.304	0.416	8.278	0.442	8.299	0.421
C171	8.74	8.402	0.338	8.507	0.233	8.359	0.381	8.702	0.038
C199	8.76	8.462	0.298	8.649	0.111	8.408	0.352	8.338	0.422
C66	8.79	8.721	0.069	8.802	-0.012	8.701	0.089	8.779	0.011
C142	8.8	8.329	0.471	8.785	0.015	8.368	0.432	8.488	0.312
C71	8.82	8.527	0.293	8.707	0.113	8.575	0.245	8.729	0.091
C118	8.9	9.15	-0.25	9.362	-0.462	9.257	-0.357	9.144	-0.244
C141	8.9	8.21	0.69	8.814	0.086	8.283	0.617	8.408	0.492
C161	8.9	9.07	-0.17	9.186	-0.286	9.217	-0.317	9.176	-0.276
C124	8.9	8.827	0.073	8.68	0.22	8.795	0.105	8.603	0.297
C163	8.9	9.138	-0.238	8.785	0.115	8.926	-0.026	9.046	-0.146
C114	8.9	8.617	0.283	8.084	0.816	8.354	0.546	8.476	0.424
C147	9	8.957	0.043	9.021	-0.021	8.916	0.084	9.068	-0.068
C210	9.09	8.486	0.604	8.703	0.387	8.549	0.541	8.612	0.478
C148	9.1	9.066	0.034	9.177	-0.077	9.1	0	9.209	-0.109
C150	9.1	9.068	0.032	9.18	-0.08	9.121	-0.021	9.226	-0.126
C121	9.2	9.1	0.1	8.598	0.602	8.796	0.404	8.723	0.477

C21	5.27	5.785	-0.515	6.542	-1.272	6.482	-1.212	6.217	-0.947
C42	5.79	6.162	-0.372	6.633	-0.843	6.539	-0.749	6.628	-0.838
C40	6.22	6.233	-0.013	6.724	-0.504	6.731	-0.511	6.435	-0.215
C44	6.3	6.549	-0.249	6.549	-0.249	6.428	-0.128	6.486	-0.186
C03	6.31	7.118	-0.808	6.338	-0.028	6.548	-0.238	6.631	-0.321
C15	6.36	6.504	-0.144	7.366	-1.006	7.287	-0.927	7.36	-1
C08	6.37	7.075	-0.705	6.769	-0.399	6.859	-0.489	6.87	-0.5
C06	6.53	6.748	-0.218	6.712	-0.182	6.53	0	6.47	0.06
C59	6.55	7.418	-0.868	7.607	-1.057	7.707	-1.157	7.526	-0.976
C75	6.74	7.382	-0.642	7.712	-0.972	7.807	-1.067	7.119	-0.379
C105	6.8	7.441	-0.641	7.268	-0.468	7.543	-0.743	7.563	-0.763
C20	7.03	7.157	-0.127	6.794	0.236	7.086	-0.056	7.126	-0.096
C43	7.07	6.799	0.271	6.692	0.378	6.684	0.386	6.534	0.536
C05	7.09	6.733	0.357	6.646	0.444	6.501	0.589	6.535	0.555
C46	7.09	7.44	-0.35	7.152	-0.062	7.118	-0.028	7.018	0.072
C07	7.11	6.773	0.337	6.645	0.465	6.539	0.571	6.526	0.584
C27	7.19	7.55	-0.36	7.575	-0.385	7.605	-0.415	7.569	-0.379
C115	7.2	8.057	-0.857	7.673	-0.473	7.913	-0.713	8.077	-0.877
C12	7.22	7.284	-0.064	7.374	-0.154	7.2	0.02	7.227	-0.007
C37	7.27	7.576	-0.306	7.819	-0.549	7.829	-0.559	7.775	-0.505
C04	7.3	7.373	-0.073	7.31	-0.01	7.335	-0.035	7.445	-0.145
C25	7.35	7.584	-0.234	7.181	0.169	7.358	-0.008	7.504	-0.154
C54	7.37	8.009	-0.639	8.1	-0.73	8.043	-0.673	7.882	-0.512
C84	7.39	7.542	-0.152	7.85	-0.46	7.732	-0.342	7.794	-0.404
C45	7.45	7.149	0.301	7.141	0.309	6.987	0.463	6.815	0.635
C26	7.5	7.237	0.263	7.542	-0.042	7.45	0.05	7.209	0.291
C82	7.58	7.497	0.083	7.646	-0.066	7.594	-0.014	7.666	-0.086
C85	7.59	7.244	0.346	7.458	0.132	7.408	0.182	7.473	0.117
C198	7.75	8.458	-0.708	8.446	-0.696	8.351	-0.601	8.413	-0.663
C10	7.76	8.118	-0.358	8.284	-0.524	7.992	-0.232	8.024	-0.264
C56	7.79	8.332	-0.542	8.121	-0.331	7.904	-0.114	7.846	-0.056
C18	7.85	7.204	0.646	6.72	1.13	7.059	0.791	7.086	0.764
C60	7.88	7.475	0.405	7.469	0.411	7.442	0.438	7.34	0.54
C196	7.99	8.415	-0.425	8.448	-0.458	8.411	-0.421	8.493	-0.503
C107	8	7.082	0.918	7.801	0.199	7.636	0.364	7.744	0.256
C139	8	8.291	-0.291	8.545	-0.545	8.015	-0.015	8.205	-0.205
C16	8.04	8.121	-0.081	8.317	-0.277	8.026	0.014	8.121	-0.081
C202	8.07	8.426	-0.356	8.641	-0.571	8.408	-0.338	8.335	-0.265
C36	8.09	7.706	0.384	7.851	0.239	8.116	-0.026	8.222	-0.132
C92	8.1	8.379	-0.279	7.963	0.137	8.25	-0.15	8.186	-0.086
C138	8.1	8.457	-0.357	8.781	-0.681	8.241	-0.141	8.281	-0.181
C110	8.1	7.992	0.108	8.334	-0.234	8.187	-0.087	8.26	-0.16
C51	8.15	7.637	0.513	7.95	0.2	7.975	0.175	7.927	0.223
C79	8.16	7.534	0.626	7.81	0.35	7.703	0.457	7.776	0.384
C23	8.19	8.143	0.047	7.993	0.197	7.825	0.365	8.043	0.147
C109	8.2	7.838	0.362	7.424	0.776	7.671	0.529	7.866	0.334

C24	8.22	7.991	0.229	7.599	0.621	7.668	0.552	7.772	0.448
C55	8.23	8.091	0.139	8.296	-0.066	8.251	-0.021	8.258	-0.028
C197	8.25	8.035	0.215	7.937	0.313	8.071	0.179	8.179	0.071
C09	8.25	8.074	0.176	8.444	-0.194	8.411	-0.161	8.16	0.09
C77	8.3	8.454	-0.154	8.564	-0.264	8.627	-0.327	8.67	-0.37
C74	8.3	8.495	-0.195	8.47	-0.17	8.311	-0.011	8.422	-0.122
C190	8.31	8.684	-0.374	8.614	-0.304	8.585	-0.275	8.505	-0.195
C181	8.31	8.548	-0.238	8.703	-0.393	8.575	-0.265	8.716	-0.406
C184	8.32	8.7	-0.38	8.561	-0.241	8.387	-0.067	8.344	-0.024
C185	8.33	8.681	-0.351	8.626	-0.296	8.408	-0.078	8.331	-0.001
C88	8.36	8.413	-0.053	8.133	0.227	8.327	0.033	8.176	0.184
C178	8.37	8.649	-0.279	8.803	-0.433	8.658	-0.288	8.791	-0.421
C94	8.39	8.565	-0.175	8.115	0.275	8.404	-0.014	8.402	-0.012
C182	8.42	8.732	-0.312	8.464	-0.044	8.438	-0.018	8.375	0.045
C90	8.43	8.515	-0.085	8.018	0.412	8.274	0.156	8.348	0.082
C168	8.44	8.105	0.335	8.253	0.187	8.25	0.19	8.35	0.09
C186	8.44	8.755	-0.315	8.481	-0.041	8.655	-0.215	8.626	-0.186
C01	8.44	8.436	0.004	8.484	-0.044	8.485	-0.045	8.608	-0.168
C188	8.44	8.684	-0.244	8.513	-0.073	8.564	-0.124	8.546	-0.106
C203	8.45	8.502	-0.052	8.507	-0.057	8.324	0.126	8.315	0.135
C175	8.45	8.427	0.023	8.482	-0.032	8.573	-0.123	8.839	-0.389
C209	8.46	8.608	-0.148	8.369	0.091	8.445	0.015	8.365	0.095
C53	8.48	8.353	0.127	8.631	-0.151	8.595	-0.115	8.615	-0.135
C58	8.48	7.804	0.676	7.913	0.567	7.93	0.55	8.026	0.454
C86	8.48	8.086	0.394	8.052	0.428	8.033	0.447	8.066	0.414
C103	8.48	8.391	0.089	8.606	-0.126	8.442	0.038	8.558	-0.078
C204	8.49	8.724	-0.234	8.23	0.26	8.602	-0.112	8.438	0.052
C69	8.5	8.518	-0.018	8.561	-0.061	8.567	-0.067	8.653	-0.153
C214	8.5	8.552	-0.052	8.552	-0.052	8.602	-0.102	8.624	-0.124
C135	8.5	8.638	-0.138	8.752	-0.252	8.744	-0.244	8.709	-0.209
C207	8.5	8.65	-0.15	8.311	0.189	8.4	0.1	8.444	0.056
C180	8.52	8.614	-0.094	8.903	-0.383	8.82	-0.3	8.893	-0.373
C192	8.52	8.764	-0.244	8.667	-0.147	8.633	-0.113	8.548	-0.028
C14	8.53	8.051	0.479	8.285	0.245	7.921	0.609	8.019	0.511
C176	8.53	8.293	0.237	8.523	0.007	8.607	-0.077	8.861	-0.331
C64	8.55	8.586	-0.036	8.732	-0.182	8.683	-0.133	8.748	-0.198
C99	8.55	8.894	-0.344	8.81	-0.26	9.109	-0.559	8.957	-0.407
C213	8.55	8.662	-0.112	8.559	-0.009	8.646	-0.096	8.741	-0.191
C194	8.56	8.634	-0.074	8.437	0.123	8.798	-0.238	8.556	0.004
C73	8.56	8.327	0.233	8.339	0.221	8.293	0.267	8.365	0.195
C195	8.56	8.615	-0.055	8.655	-0.095	8.466	0.094	8.386	0.174
C33	8.56	8.717	-0.157	8.862	-0.302	8.868	-0.308	8.434	0.126
C100	8.58	8.126	0.454	8.398	0.182	8.405	0.175	8.539	0.041
C57	8.58	8.345	0.235	8.218	0.362	7.919	0.661	7.782	0.798
C76	8.58	8.259	0.321	8.876	-0.296	8.891	-0.311	8.7	-0.12
C95	8.58	8.626	-0.046	8.356	0.224	8.561	0.019	8.617	-0.037

C208	8.6	8.596	0.004	8.463	0.137	8.313	0.287	8.274	0.326
C129	8.6	8.726	-0.126	8.66	-0.06	8.764	-0.164	8.584	0.016
C140	8.6	8.175	0.425	8.698	-0.098	8.141	0.459	8.342	0.258
C63	8.6	8.267	0.333	8.557	0.043	8.617	-0.017	8.681	-0.081
C89	8.61	8.313	0.297	7.978	0.632	8.159	0.451	8.18	0.43
C91	8.61	8.462	0.148	8.07	0.54	8.375	0.235	8.334	0.276
C169	8.63	8.399	0.231	8.462	0.168	8.313	0.317	8.359	0.271
C67	8.65	8.506	0.144	8.791	-0.141	8.767	-0.117	8.689	-0.039
C31	8.65	8.848	-0.198	8.44	0.21	8.636	0.014	8.373	0.277
C29	8.65	8.511	0.139	8.645	0.005	8.116	0.534	8.304	0.346
C30	8.67	8.169	0.501	8.635	0.035	8.763	-0.093	8.794	-0.124
C212	8.67	8.686	-0.016	8.693	-0.023	9.143	-0.473	8.982	-0.312
C173	8.67	8.615	0.055	8.558	0.112	8.65	0.02	8.755	-0.085
C102	8.67	8.369	0.301	8.664	0.006	8.476	0.194	8.583	0.087
C174	8.69	8.624	0.066	8.798	-0.108	8.808	-0.118	8.659	0.031
C128	8.7	8.858	-0.158	8.675	0.025	8.767	-0.067	8.595	0.105
C177	8.72	8.494	0.226	8.453	0.267	8.41	0.31	8.491	0.229
C172	8.74	8.69	0.05	8.701	0.039	8.692	0.048	8.73	0.01
C187	8.74	8.822	-0.082	8.506	0.234	8.706	0.034	8.626	0.114
C170	8.74	8.567	0.173	8.359	0.381	8.301	0.439	8.55	0.19
C191	8.76	8.804	-0.044	8.623	0.137	8.603	0.157	8.572	0.188
C70	8.78	8.66	0.12	8.647	0.133	8.604	0.176	8.58	0.2
C137	8.8	8.836	-0.036	8.902	-0.102	9.042	-0.242	8.921	-0.121
C97	8.82	8.566	0.254	8.175	0.645	8.539	0.281	8.645	0.175
C32	8.88	9.05	-0.17	8.586	0.294	8.605	0.275	8.642	0.238
C205	8.88	8.734	0.146	8.613	0.267	8.717	0.163	8.66	0.22
C153	8.9	9.068	-0.168	9.196	-0.296	9.151	-0.251	9.081	-0.181
C151	8.9	8.893	0.007	9.04	-0.14	9.047	-0.147	8.979	-0.079
C136	8.9	8.77	0.13	8.711	0.189	8.714	0.186	8.672	0.228
C127	8.9	8.814	0.086	8.687	0.213	8.796	0.104	8.602	0.298
C111	8.9	8.678	0.222	8.438	0.462	8.625	0.275	8.731	0.169
C120	8.9	9.112	-0.212	8.83	0.07	8.808	0.092	8.947	-0.047
C179	8.92	8.681	0.239	8.552	0.368	8.602	0.318	8.619	0.301
C68	8.92	8.592	0.328	8.858	0.062	8.784	0.136	8.717	0.203
C98	8.92	8.982	-0.062	8.751	0.169	9.024	-0.104	9.016	-0.096
C145	9	9.122	-0.122	9.203	-0.203	9.228	-0.228	9.138	-0.138
C38	9	9.1	-0.1	8.967	0.033	8.676	0.324	8.692	0.308
C155	9	9.124	-0.124	8.892	0.108	8.916	0.084	8.952	0.048
C154	9	9.096	-0.096	9.137	-0.137	9.139	-0.139	9.171	-0.171
C160	9	8.82	0.18	9.105	-0.105	9.156	-0.156	9.102	-0.102
C152	9	8.812	0.188	8.752	0.248	8.938	0.062	8.825	0.175
C162	9	8.998	0.002	8.995	0.005	9.087	-0.087	9.027	-0.027
C122	9	9.308	-0.308	8.89	0.11	8.888	0.112	8.97	0.03
C132	9	8.797	0.203	9.107	-0.107	9.019	-0.019	8.984	0.016
C133	9	8.665	0.335	8.952	0.048	8.653	0.347	8.61	0.39
C144	9	8.969	0.031	9.192	-0.192	9.195	-0.195	9.326	-0.326

C157	9	9.099	-0.099	9.035	-0.035	9.049	-0.049	9.131	-0.131
C123	9	9.018	-0.018	8.991	0.009	9.071	-0.071	9.094	-0.094
C164	9	9.153	-0.153	9.032	-0.032	9.11	-0.11	9.145	-0.145
C156	9	9.2	-0.2	9.206	-0.206	9.168	-0.168	9.263	-0.263
C131	9	8.88	0.12	9.1	-0.1	9.043	-0.043	9.009	-0.009
C211	9.04	8.801	0.239	8.688	0.352	8.97	0.07	8.863	0.177
C206	9.04	8.749	0.291	8.382	0.658	8.652	0.388	8.538	0.502
C200	9.09	8.42	0.67	8.354	0.736	8.212	0.878	8.147	0.943
C134	9.1	8.762	0.338	8.943	0.157	8.938	0.162	8.975	0.125
C149	9.1	8.966	0.134	9.015	0.085	8.985	0.115	9.083	0.017
C112	9.1	8.957	0.143	8.537	0.563	8.66	0.44	8.759	0.341
C143	9.1	8.298	0.802	8.706	0.394	8.218	0.882	8.58	0.52
C117	9.1	9.218	-0.118	9.27	-0.17	9.167	-0.067	9.089	0.011
C125	9.1	8.97	0.13	8.672	0.428	8.818	0.282	8.676	0.424
C159	9.1	9.124	-0.024	9.008	0.092	9.091	0.009	9.192	-0.092
C126	9.1	8.944	0.156	9.106	-0.006	9.053	0.047	9.036	0.064
C158	9.1	9.195	-0.095	9.2	-0.1	9.222	-0.122	9.341	-0.241
C119	9.1	9.058	0.042	8.705	0.395	8.76	0.34	8.834	0.266
C113	9.2	8.717	0.483	8.612	0.588	8.529	0.671	8.64	0.56
C146	9.2	9.21	-0.01	8.978	0.222	9.147	0.053	9.248	-0.048
C116	9.2	9.064	0.136	9.425	-0.225	9.335	-0.135	9.213	-0.013

#Cpd: Compounds

Table S11: Actual pIC_{50} vs. Predicted pIC_{50} of the compounds from the CoMFA and CoMSIA training set compounds of SET-D.

#Cpd	Actual pIC_{50}	CoMFA		Predicted pIC_{50} (CoMSIA)					
		Predicted pIC_{50}	Residuals	SD	Residuals	SEAD	Residuals	SHAD	Residuals
C40	6.22	6.287	-0.067	6.682	-0.462	6.328	-0.108	6.157	0.063
C15	6.36	7.233	-0.873	7.628	-1.268	7.861	-1.501	7.961	-1.601
C08	6.37	7.108	-0.738	6.941	-0.571	6.997	-0.627	7.148	-0.778
C06	6.53	6.7	-0.17	6.886	-0.356	6.714	-0.184	7.09	-0.56
C43	7.07	6.768	0.302	6.728	0.342	6.519	0.551	6.467	0.603
C07	7.11	6.509	0.601	6.717	0.393	6.672	0.438	7.024	0.086
C19	7.15	6.76	0.39	6.745	0.405	7.07	0.08	7.049	0.101
C115	7.2	8.34	-1.14	8.467	-1.267	8.548	-1.348	8.503	-1.303
C04	7.3	7.381	-0.081	7.371	-0.071	7.533	-0.233	7.234	0.066
C25	7.35	7.676	-0.326	7.443	-0.093	7.991	-0.641	7.936	-0.586
C84	7.39	8.066	-0.676	7.868	-0.478	7.935	-0.545	7.864	-0.474
C107	8	6.644	1.356	7.847	0.153	7.635	0.365	7.691	0.309
C110	8.1	7.702	0.398	8.189	-0.089	8.239	-0.139	8.325	-0.225
C109	8.2	7.541	0.659	7.157	1.043	7.669	0.531	7.391	0.809
C24	8.22	8.022	0.198	7.813	0.407	8.011	0.209	7.502	0.718
C197	8.25	7.97	0.28	8.026	0.224	8.189	0.061	7.966	0.284

C74	8.3	8.569	-0.269	8.511	-0.211	8.439	-0.139	8.393	-0.093
C190	8.31	8.619	-0.309	8.564	-0.254	8.478	-0.168	8.503	-0.193
C185	8.33	8.618	-0.288	8.571	-0.241	8.307	0.023	8.194	0.136
C88	8.36	8.516	-0.156	8.09	0.27	8.181	0.179	8.279	0.081
C178	8.37	8.369	0.001	8.577	-0.207	8.708	-0.338	8.66	-0.29
C186	8.44	8.491	-0.051	8.41	0.03	8.494	-0.054	8.31	0.13
C188	8.44	8.52	-0.08	8.456	-0.016	8.454	-0.014	8.369	0.071
C203	8.45	8.602	-0.152	8.555	-0.105	8.502	-0.052	8.613	-0.163
C175	8.45	8.322	0.128	8.514	-0.064	8.796	-0.346	8.721	-0.271
C103	8.48	8.585	-0.105	8.557	-0.077	8.486	-0.006	8.458	0.022
C69	8.5	8.597	-0.097	8.413	0.087	8.577	-0.077	8.574	-0.074
C207	8.5	8.305	0.195	8.175	0.325	8.339	0.161	8.465	0.035
C192	8.52	8.658	-0.138	8.622	-0.102	8.536	-0.016	8.529	-0.009
C195	8.56	8.716	-0.156	8.794	-0.234	8.542	0.018	8.535	0.025
C95	8.58	8.754	-0.174	8.284	0.296	8.635	-0.055	8.704	-0.124
C129	8.6	8.817	-0.217	8.84	-0.24	8.69	-0.09	8.585	0.015
C140	8.6	8.212	0.388	8.584	0.016	8.237	0.363	8.615	-0.015
C63	8.6	8.466	0.134	8.399	0.201	8.628	-0.028	8.579	0.021
C183	8.61	8.349	0.261	8.309	0.301	8.36	0.25	8.329	0.281
C173	8.67	8.226	0.444	8.217	0.453	8.442	0.228	8.313	0.357
C174	8.69	8.273	0.417	8.348	0.342	8.338	0.352	8.261	0.429
C177	8.72	8.331	0.389	8.372	0.348	8.401	0.319	8.357	0.363
C70	8.78	8.683	0.097	8.43	0.35	8.387	0.393	8.381	0.399
C137	8.8	8.866	-0.066	8.933	-0.133	8.902	-0.102	8.911	-0.111
C118	8.9	8.967	-0.067	9.409	-0.509	9.22	-0.32	9.36	-0.46
C98	8.92	8.903	0.017	8.605	0.315	8.899	0.021	8.712	0.208
C155	9	9.131	-0.131	8.925	0.075	8.902	0.098	8.941	0.059
C157	9	9.207	-0.207	9.046	-0.046	9.132	-0.132	9.166	-0.166
C123	9	8.951	0.049	8.979	0.021	9.068	-0.068	8.899	0.101
C164	9	9.202	-0.202	9.041	-0.041	9.101	-0.101	9.17	-0.17
C206	9.04	8.236	0.804	7.946	1.094	8.258	0.782	8.227	0.813
C134	9.1	8.444	0.656	8.756	0.344	8.757	0.343	8.74	0.36
C112	9.1	8.586	0.514	8.643	0.457	8.795	0.305	8.868	0.232
C143	9.1	8.305	0.795	8.609	0.491	8.351	0.749	8.717	0.383
C125	9.1	8.934	0.166	8.846	0.254	8.77	0.33	8.652	0.448
C126	9.1	8.945	0.155	9.06	0.04	9.017	0.083	8.917	0.183
C158	9.1	9.105	-0.005	9.155	-0.055	9.202	-0.102	9.139	-0.039
C116	9.2	8.657	0.543	9.271	-0.071	9.186	0.014	9.262	-0.062
C21	5.27	5.901	-0.631	6.667	-1.397	6.221	-0.951	6.336	-1.066
C42	5.79	5.939	-0.149	6.582	-0.792	6.633	-0.843	6.639	-0.849
C41	6.27	6.479	-0.209	6.618	-0.348	6.682	-0.412	6.352	-0.082
C44	6.3	6.48	-0.18	6.262	0.038	6.363	-0.063	6.232	0.068
C03	6.31	7.19	-0.88	6.597	-0.287	6.795	-0.485	6.86	-0.55
C59	6.55	7.488	-0.938	7.495	-0.945	7.476	-0.926	7.544	-0.994
C75	6.74	7.544	-0.804	7.598	-0.858	7.103	-0.363	6.781	-0.041
C105	6.8	7.094	-0.294	7.129	-0.329	7.406	-0.606	7.329	-0.529

C20	7.03	7.085	-0.055	6.809	0.221	7.19	-0.16	7.092	-0.062
C05	7.09	6.623	0.467	6.718	0.372	6.705	0.385	6.818	0.272
C46	7.09	7.318	-0.228	7.05	0.04	6.896	0.194	7.035	0.055
C27	7.19	7.688	-0.498	7.761	-0.571	7.752	-0.562	7.55	-0.36
C12	7.22	7.357	-0.137	7.439	-0.219	7.232	-0.012	7.32	-0.1
C37	7.27	7.485	-0.215	7.905	-0.635	7.728	-0.458	7.513	-0.243
C81	7.3	7.306	-0.006	7.447	-0.147	7.472	-0.172	7.363	-0.063
C47	7.3	7.194	0.106	7.073	0.227	7.143	0.157	7.245	0.055
C54	7.37	8.037	-0.667	8.029	-0.659	7.808	-0.438	7.701	-0.331
C45	7.45	7.039	0.411	7.068	0.382	6.761	0.689	6.951	0.499
C165	7.46	8.328	-0.868	8.247	-0.787	8.258	-0.798	8.139	-0.679
C167	7.46	8.125	-0.665	8.511	-1.051	8.544	-1.084	8.54	-1.08
C52	7.48	7.191	0.289	7.912	-0.432	7.195	0.285	7.04	0.44
C26	7.5	7.406	0.094	7.596	-0.096	7.273	0.227	7.178	0.322
C83	7.55	7.881	-0.331	7.835	-0.285	7.917	-0.367	7.972	-0.422
C82	7.58	7.635	-0.055	7.646	-0.066	7.796	-0.216	7.696	-0.116
C85	7.59	7.623	-0.033	7.623	-0.033	7.693	-0.103	7.723	-0.133
C108	7.6	7.617	-0.017	7.159	0.441	7.247	0.353	7.182	0.418
C198	7.75	8.362	-0.612	8.363	-0.613	8.307	-0.557	8.371	-0.621
C10	7.76	8.164	-0.404	8.407	-0.647	8.16	-0.4	8.426	-0.666
C56	7.79	8.396	-0.606	8.091	-0.301	7.893	-0.103	7.891	-0.101
C11	7.82	7.97	-0.15	8.11	-0.29	8.164	-0.344	8.396	-0.576
C18	7.85	7.224	0.626	6.793	1.057	7.017	0.833	6.881	0.969
C60	7.88	7.39	0.49	7.39	0.49	7.299	0.581	7.383	0.497
C61	7.92	7.838	0.082	8.165	-0.245	7.524	0.396	7.818	0.102
C196	7.99	8.487	-0.497	8.389	-0.399	8.433	-0.443	8.389	-0.399
C139	8	8.283	-0.283	8.341	-0.341	8.19	-0.19	8.53	-0.53
C80	8.04	7.851	0.189	7.766	0.274	8.013	0.027	7.913	0.127
C16	8.04	8.09	-0.05	8.111	-0.071	8.196	-0.156	8.329	-0.289
C50	8.04	8.505	-0.465	8.438	-0.398	8.696	-0.656	8.447	-0.407
C202	8.07	8.693	-0.623	8.73	-0.66	8.45	-0.38	8.495	-0.425
C36	8.09	7.671	0.419	7.953	0.137	8.342	-0.252	8.443	-0.353
C48	8.09	7.847	0.243	8.438	-0.348	8.188	-0.098	8.144	-0.054
C92	8.1	8.343	-0.243	8.056	0.044	8.258	-0.158	8.333	-0.233
C138	8.1	8.488	-0.388	8.639	-0.539	8.268	-0.168	8.608	-0.508
C28	8.12	7.857	0.263	7.684	0.436	7.954	0.166	7.808	0.312
C17	8.13	8.426	-0.296	8.402	-0.272	8.205	-0.075	8.271	-0.141
C51	8.15	7.633	0.517	7.914	0.236	7.888	0.262	7.739	0.411
C79	8.16	7.66	0.5	7.739	0.421	7.863	0.297	7.911	0.249
C35	8.18	8.677	-0.497	8.551	-0.371	8.405	-0.225	8.284	-0.104
C23	8.19	8.258	-0.068	8.165	0.025	8.286	-0.096	8.407	-0.217
C78	8.22	7.795	0.425	7.771	0.449	7.881	0.339	7.942	0.278
C55	8.23	8.259	-0.029	8.263	-0.033	8.296	-0.066	8.305	-0.075
C09	8.25	7.983	0.267	8.406	-0.156	8.174	0.076	8.324	-0.074
C77	8.3	8.417	-0.117	8.374	-0.074	8.584	-0.284	8.573	-0.273
C181	8.31	8.41	-0.1	8.533	-0.223	8.679	-0.369	8.709	-0.399

C184	8.32	8.672	-0.352	8.507	-0.187	8.302	0.018	8.166	0.154
C62	8.34	7.909	0.431	8.227	0.113	7.713	0.627	8.254	0.086
C13	8.35	8.113	0.237	8.407	-0.057	8.191	0.159	8.24	0.11
C193	8.37	8.666	-0.296	8.608	-0.238	8.527	-0.157	8.524	-0.154
C87	8.37	8.359	0.011	8.232	0.138	8.124	0.246	8.269	0.101
C72	8.39	8.577	-0.187	8.638	-0.248	8.685	-0.295	8.616	-0.226
C94	8.39	8.467	-0.077	8.181	0.209	8.481	-0.091	8.577	-0.187
C96	8.39	8.583	-0.193	8.397	-0.007	8.437	-0.047	8.461	-0.071
C182	8.42	8.494	-0.074	8.398	0.022	8.253	0.167	8.195	0.225
C90	8.43	8.376	0.054	7.997	0.433	8.292	0.138	8.398	0.032
C104	8.44	8.615	-0.175	8.574	-0.134	8.668	-0.228	8.498	-0.058
C168	8.44	8.28	0.16	8.362	0.078	8.364	0.076	8.341	0.099
C01	8.44	8.571	-0.131	8.591	-0.151	8.71	-0.27	8.593	-0.153
C209	8.46	8.642	-0.182	8.273	0.187	8.352	0.108	8.468	-0.008
C166	8.46	8.381	0.079	8.214	0.246	8.184	0.276	8.067	0.393
C49	8.46	8.19	0.27	8.098	0.362	8.454	0.006	7.963	0.497
C53	8.48	8.35	0.13	8.441	0.039	8.61	-0.13	8.546	-0.066
C58	8.48	7.887	0.593	7.86	0.62	8.172	0.308	8.157	0.323
C201	8.48	8.659	-0.179	8.65	-0.17	8.396	0.084	8.409	0.071
C101	8.48	8.557	-0.077	8.592	-0.112	8.561	-0.081	8.471	0.009
C86	8.48	8.218	0.262	8.028	0.452	8.121	0.359	8.292	0.188
C204	8.49	8.565	-0.075	8.302	0.188	8.48	0.01	8.307	0.183
C214	8.5	8.801	-0.301	8.488	0.012	8.638	-0.138	8.757	-0.257
C135	8.5	8.409	0.091	8.617	-0.117	8.597	-0.097	8.724	-0.224
C180	8.52	8.338	0.182	8.469	0.051	8.594	-0.074	8.611	-0.091
C14	8.53	8.242	0.288	8.408	0.122	8.194	0.336	8.358	0.172
C176	8.53	8.404	0.126	8.5	0.03	8.815	-0.285	8.889	-0.359
C22	8.53	8.686	-0.156	8.405	0.125	8.667	-0.137	8.726	-0.196
C64	8.55	8.558	-0.008	8.587	-0.037	8.717	-0.167	8.672	-0.122
C99	8.55	8.691	-0.141	8.645	-0.095	8.837	-0.287	8.637	-0.087
C215	8.55	8.56	-0.01	8.444	0.106	8.629	-0.079	8.548	0.002
C213	8.55	8.791	-0.241	8.496	0.054	8.692	-0.142	8.781	-0.231
C194	8.56	8.555	0.005	8.4	0.16	8.619	-0.059	8.54	0.02
C73	8.56	8.323	0.237	8.407	0.153	8.392	0.168	8.272	0.288
C33	8.56	8.605	-0.045	8.624	-0.064	8.381	0.179	8.593	-0.033
C100	8.58	8.203	0.377	8.395	0.185	8.464	0.116	8.353	0.227
C57	8.58	8.374	0.206	8.142	0.438	7.795	0.785	8.129	0.451
C76	8.58	8.224	0.356	8.65	-0.07	8.641	-0.061	8.604	-0.024
C208	8.6	8.628	-0.028	8.382	0.218	8.274	0.326	8.381	0.219
C130	8.6	8.639	-0.039	9.055	-0.455	8.938	-0.338	8.851	-0.251
C93	8.6	8.204	0.396	8.701	-0.101	8.494	0.106	8.593	0.007
C89	8.61	8.274	0.336	7.948	0.662	8.168	0.442	8.35	0.26
C189	8.61	8.63	-0.02	8.482	0.128	8.485	0.125	8.515	0.095
C91	8.61	8.4	0.21	8.046	0.564	8.303	0.307	8.332	0.278
C169	8.63	8.496	0.134	8.392	0.238	8.311	0.319	8.27	0.36
C67	8.65	8.495	0.155	8.626	0.024	8.654	-0.004	8.596	0.054

C31	8.65	8.796	-0.146	8.415	0.235	8.348	0.302	8.239	0.411
C29	8.65	8.642	0.008	8.678	-0.028	8.391	0.259	8.472	0.178
C39	8.67	8.821	-0.151	8.674	-0.004	8.768	-0.098	8.544	0.126
C30	8.67	8.179	0.491	8.72	-0.05	8.613	0.057	8.393	0.277
C212	8.67	8.617	0.053	8.664	0.006	8.94	-0.27	8.732	-0.062
C102	8.67	8.448	0.222	8.603	0.067	8.51	0.16	8.486	0.184
C65	8.67	8.593	0.077	8.769	-0.099	8.803	-0.133	8.835	-0.165
C128	8.7	8.797	-0.097	8.79	-0.09	8.69	0.01	8.603	0.097
C34	8.72	8.475	0.245	8.427	0.293	8.384	0.336	8.399	0.321
C171	8.74	8.544	0.196	8.435	0.305	8.708	0.032	8.801	-0.061
C172	8.74	8.657	0.083	8.53	0.21	8.585	0.155	8.62	0.12
C187	8.74	8.769	-0.029	8.451	0.289	8.51	0.23	8.328	0.412
C170	8.74	8.528	0.212	8.268	0.472	8.505	0.235	8.65	0.09
C191	8.76	8.667	0.093	8.566	0.194	8.545	0.215	8.557	0.203
C199	8.76	8.65	0.11	8.723	0.037	8.448	0.312	8.535	0.225
C66	8.79	8.698	0.092	8.694	0.096	8.768	0.022	8.799	-0.009
C142	8.8	8.359	0.441	8.84	-0.04	8.449	0.351	8.808	-0.008
C97	8.82	8.517	0.303	8.147	0.673	8.738	0.082	8.786	0.034
C71	8.82	8.564	0.256	8.63	0.19	8.732	0.088	8.851	-0.031
C32	8.88	9.159	-0.279	8.585	0.295	8.812	0.068	8.646	0.234
C205	8.88	8.611	0.269	8.631	0.249	8.64	0.24	8.634	0.246
C153	8.9	9.079	-0.179	9.157	-0.257	8.968	-0.068	8.986	-0.086
C141	8.9	8.252	0.648	8.76	0.14	8.287	0.613	8.711	0.189
C161	8.9	9.012	-0.112	9.132	-0.232	9.059	-0.159	8.915	-0.015
C151	8.9	8.917	-0.017	8.932	-0.032	9.029	-0.129	9.087	-0.187
C136	8.9	8.596	0.304	8.592	0.308	8.569	0.331	8.646	0.254
C127	8.9	8.802	0.098	8.823	0.077	8.713	0.187	8.662	0.238
C124	8.9	8.812	0.088	8.821	0.079	8.707	0.193	8.659	0.241
C111	8.9	8.639	0.261	8.525	0.375	8.759	0.141	8.666	0.234
C163	8.9	9.109	-0.209	8.716	0.184	9.009	-0.109	9.069	-0.169
C114	8.9	8.647	0.253	8.299	0.601	8.55	0.35	8.367	0.533
C120	8.9	9.113	-0.213	8.92	-0.02	9.048	-0.148	9.179	-0.279
C179	8.92	8.481	0.439	8.266	0.654	8.367	0.553	8.298	0.622
C68	8.92	8.575	0.345	8.738	0.182	8.704	0.216	8.726	0.194
C145	9	9.088	-0.088	9.16	-0.16	9.059	-0.059	8.91	0.09
C38	9	9.057	-0.057	8.997	0.003	8.728	0.272	8.596	0.404
C154	9	9.088	-0.088	9.17	-0.17	9.133	-0.133	9.142	-0.142
C160	9	8.806	0.194	8.905	0.095	8.973	0.027	9.033	-0.033
C152	9	8.761	0.239	8.719	0.281	8.834	0.166	8.869	0.131
C162	9	8.913	0.087	9.01	-0.01	8.913	0.087	8.855	0.145
C122	9	9.396	-0.396	9.234	-0.234	9.22	-0.22	9.132	-0.132
C132	9	8.764	0.236	9.062	-0.062	8.991	0.009	8.939	0.061
C133	9	8.705	0.295	9.195	-0.195	8.712	0.288	8.699	0.301
C144	9	8.95	0.05	9.167	-0.167	9.273	-0.273	9.105	-0.105
C156	9	9.213	-0.213	9.152	-0.152	9.266	-0.266	9.217	-0.217
C147	9	9.064	-0.064	8.985	0.015	9.048	-0.048	8.966	0.034

C131	9	8.842	0.158	9.069	-0.069	9.014	-0.014	8.926	0.074
C211	9.04	8.707	0.333	8.666	0.374	8.867	0.173	8.738	0.302
C200	9.09	8.672	0.418	8.594	0.496	8.336	0.754	8.305	0.785
C210	9.09	8.887	0.203	8.774	0.316	8.78	0.31	8.935	0.155
C148	9.1	9.159	-0.059	9.157	-0.057	9.211	-0.111	9.123	-0.023
C149	9.1	9.044	0.056	9.058	0.042	9.08	0.02	9.076	0.024
C150	9.1	9.121	-0.021	9.146	-0.046	9.228	-0.128	9.167	-0.067
C117	9.1	9.168	-0.068	9.329	-0.229	9.166	-0.066	9.272	-0.172
C159	9.1	9.034	0.066	9.033	0.067	9.055	0.045	9.079	0.021
C119	9.1	9.038	0.062	8.819	0.281	8.948	0.152	9.076	0.024
C113	9.2	8.949	0.251	8.725	0.475	8.746	0.454	8.808	0.392
C146	9.2	9.234	-0.034	9.101	0.099	9.257	-0.057	9.087	0.113
C121	9.2	9.1	0.1	9.041	0.159	9.061	0.139	8.992	0.208

#Cpd: Compounds

Table S12. Structure and activity values of PI3Kδ inhibitors.

#Cpd	Structure	pIC_{50}	#Cpd	Structure	pIC_{50}
01	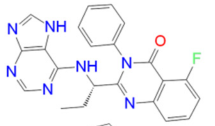	8.22	108	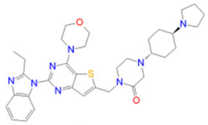	7.85
02	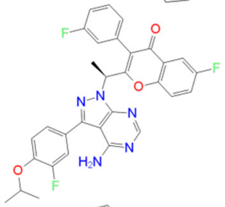	7.31	109	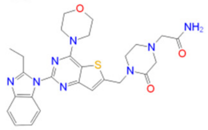	8.20
03	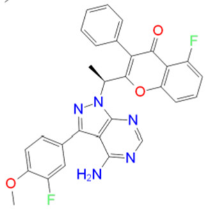	7.21	110	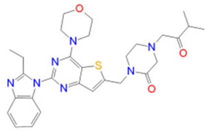	8.44
04	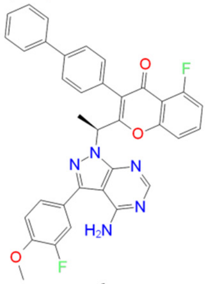	NA	111	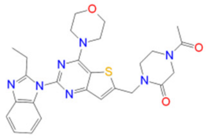	8.49
05	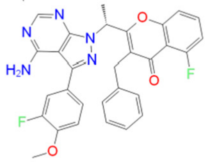	5.62	112	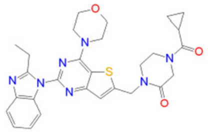	8.95
06	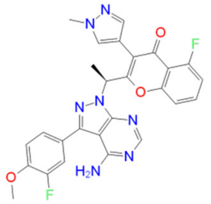	7.27	113	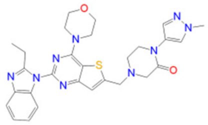	8.46
07	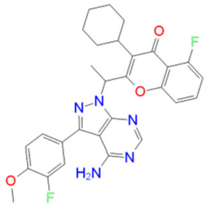	6.21	114	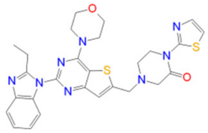	7.08

08	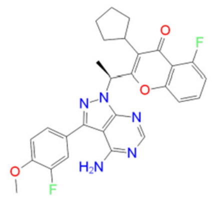	7.58	115	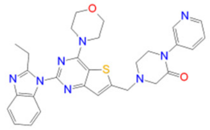	7.88
09	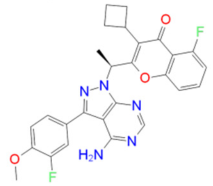	7.48	116	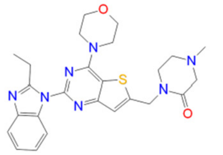	8.34
10	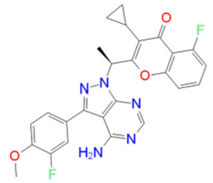	7.74	117	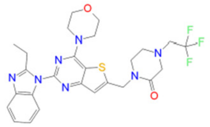	8.20
11	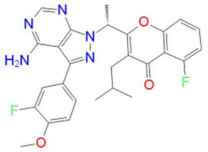	7.39	118	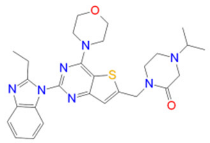	8.67
12	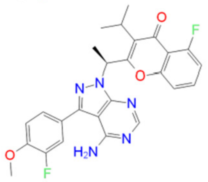	7.29	119	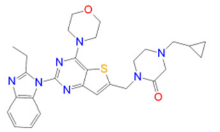	8.65
13	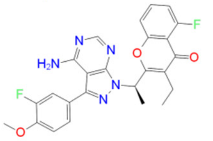	7.34	120	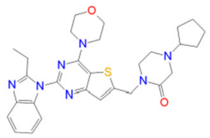	8.53
14	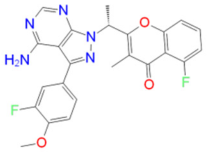	7.27	121	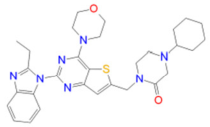	7.92
15	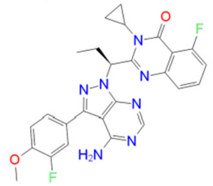	7.76	122	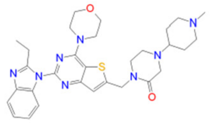	7.82

16	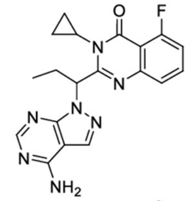	NA	123	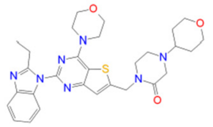	8.20
17	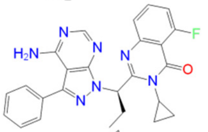	5.65	124	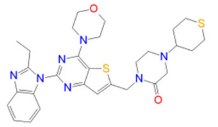	8.31
18	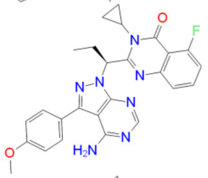	7.53	125	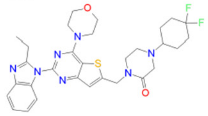	8.42
19	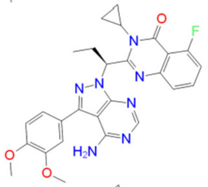	7.49	126	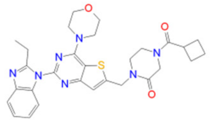	8.85
20	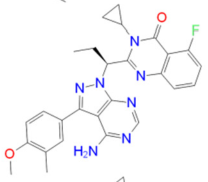	7.07	127	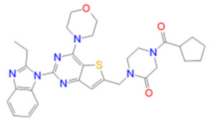	8.69
21	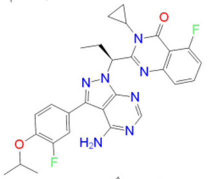	5.75	128	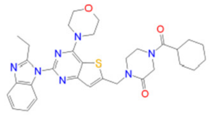	8.10
22	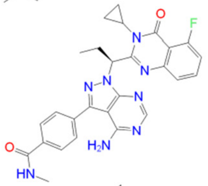	6.27	129	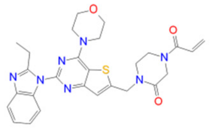	8.53
23	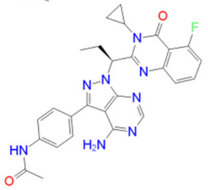	6.80	130	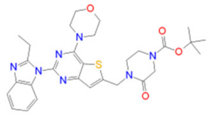	7.92

24	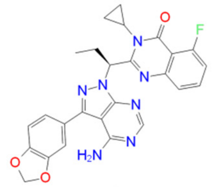	5.81	131	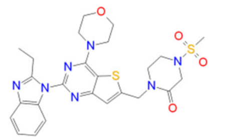	8.65
25	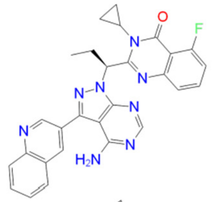	5.65	132	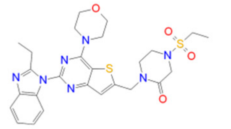	8.35
26	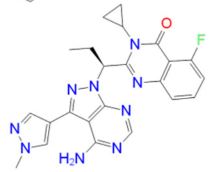	7.46	133	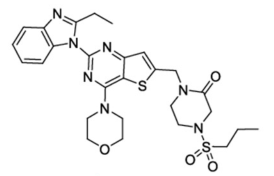	8.45
27	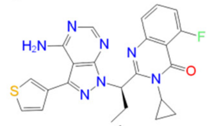	6.06	134	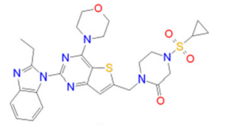	8.92
28	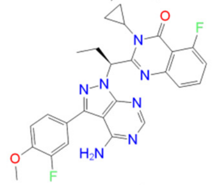	7.72	135	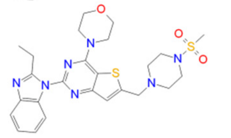	8.38
29	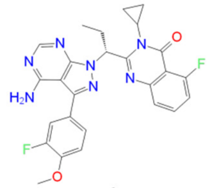	7.85	136	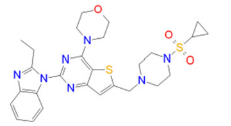	8.31
30	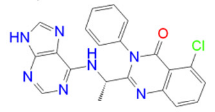	9	137	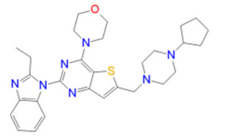	7.37
31	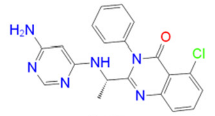	7.00	138	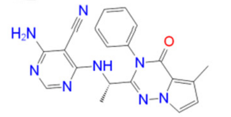	8.40
32	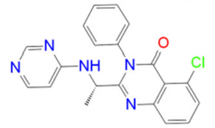	5.76	139	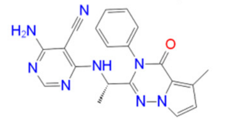	9.0

33	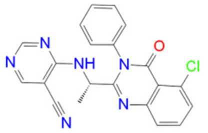	8.09	140	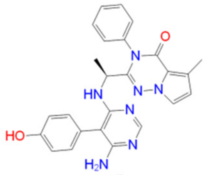	7.65
34	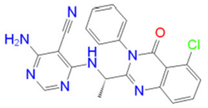	9.39	141	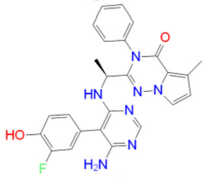	9.30
35	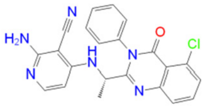	7.52	142	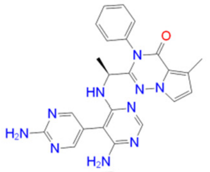	8.30
36	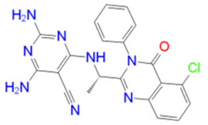	8.60	143	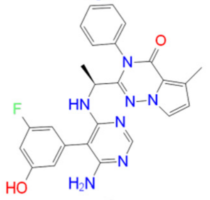	9.22
37	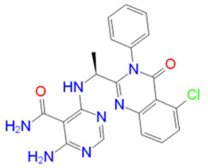	7.95	144	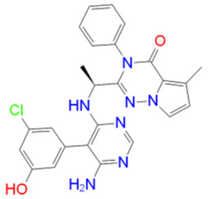	9.69
38	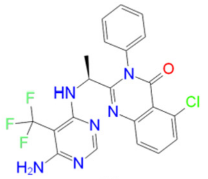	8.74	145	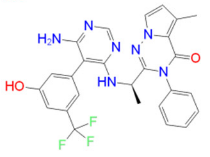	9.69
39	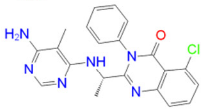	7.85	146	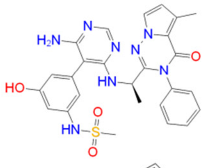	9.22
40	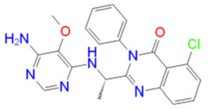	7.40	147	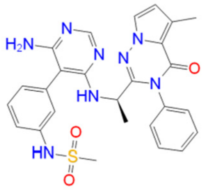	9.52

41	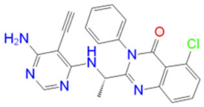	10.0	148	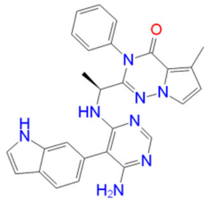	8.52
42	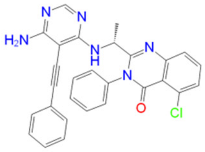	9.52	149	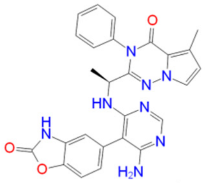	7.95
43	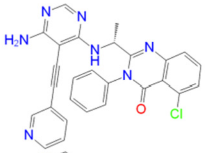	9.30	150	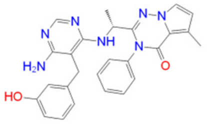	7.65
44	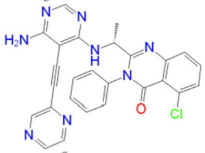	9.39	151	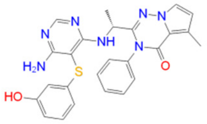	8.74
45	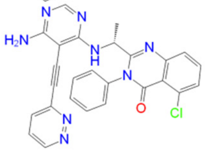	6.61	152	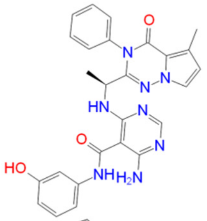	9.69
46	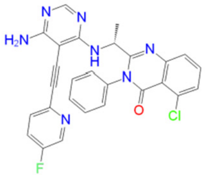	8.92	153	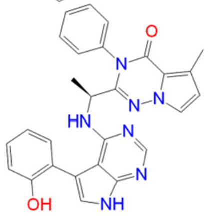	8.29
47	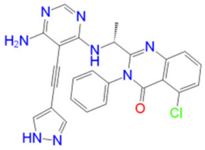	9.52	154	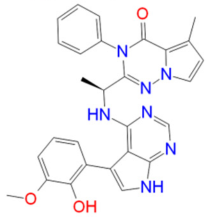	9.69

48	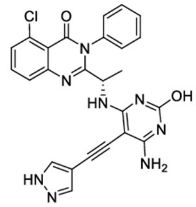	NA	155	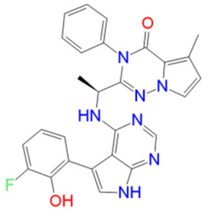	8.72
49	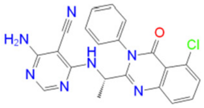	8.92	156	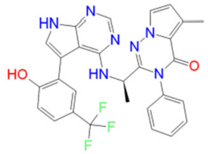	9.52
50	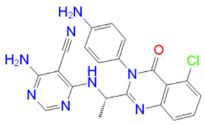	10.0	157	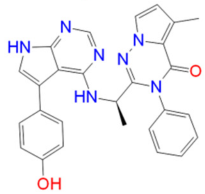	9.39
51	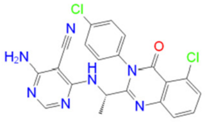	8.36	158	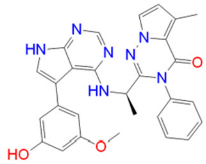	9.69
52	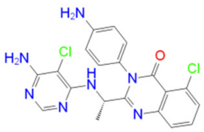	9.04	159	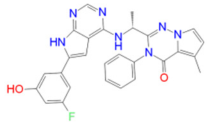	9.69
53	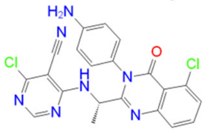	9.0	160	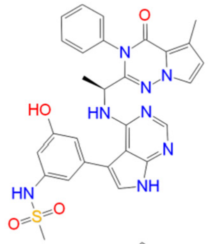	9.69
54	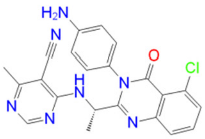	9.88	161	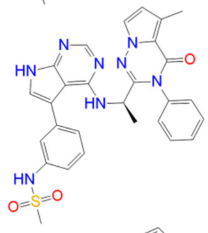	9.52
55	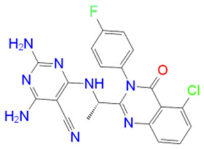	8.69	162	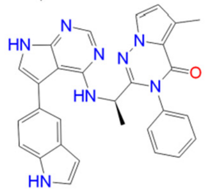	8.88

56	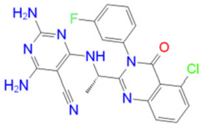	9.82	163	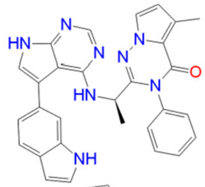	9.69
57	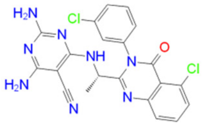	9.34	164	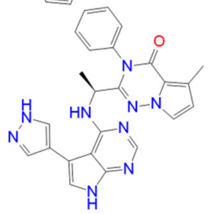	9.39
58	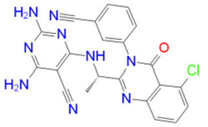	8.72	165	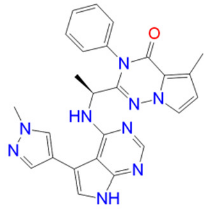	9.69
59	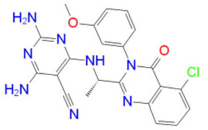	9.04	166	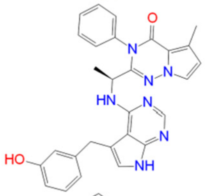	8.85
60	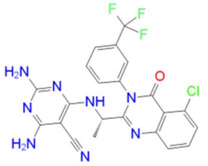	8.44	167	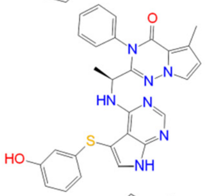	9.0
61	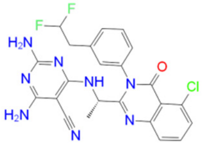	8.79	168	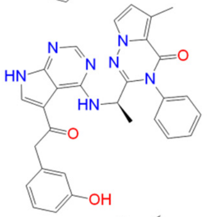	8.69
62	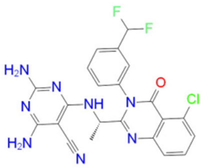	9.26	169	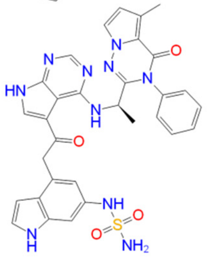	9.30

63	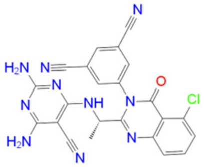	6.82	170	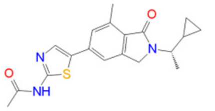	6.5
64	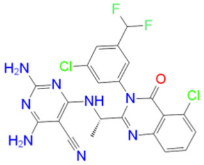	8.82	171	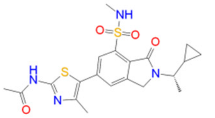	6.8
65	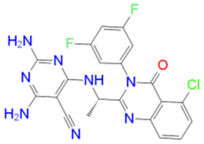	9.32	172	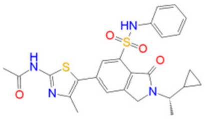	7.3
66	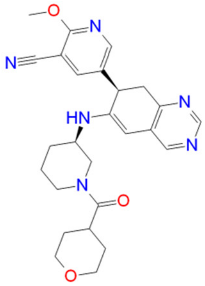	8.1	173	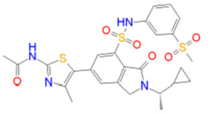	8.5
67	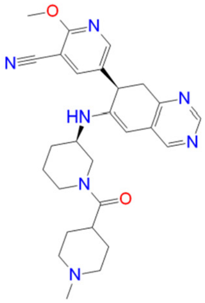	8.02	174	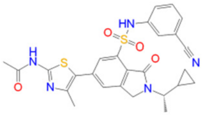	8.0
68	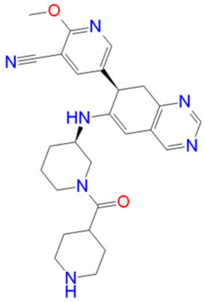	8.60	175	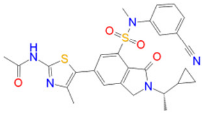	7.3

69	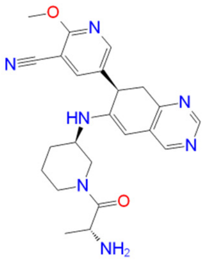	8.43	176	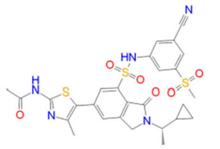	7.6
70	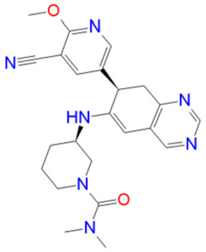	8.88	177	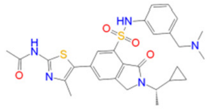	7.8
71	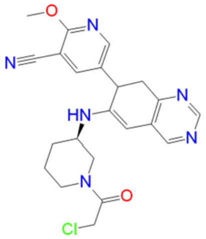	8.05	178	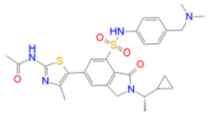	8.2
72	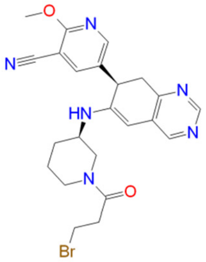	7.84	179	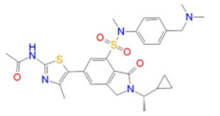	7.6
73	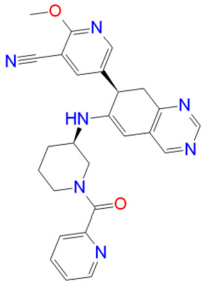	9.15	180	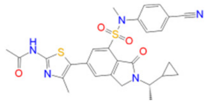	6.8

74	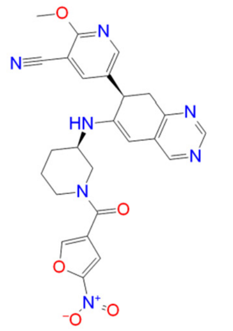	8.40	181	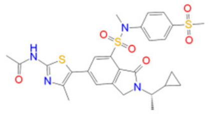	6.8
75	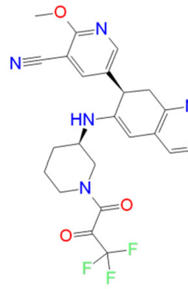	8.49	182	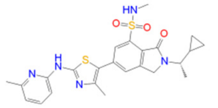	7.3
76	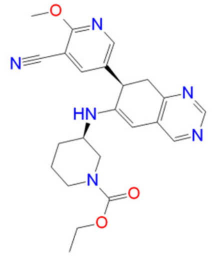	8.49	183	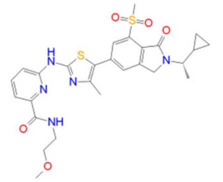	8.3
77	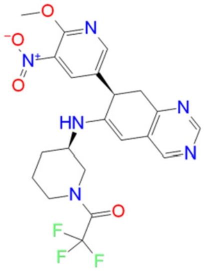	7.56	184	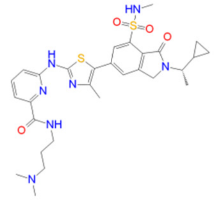	8.4
78	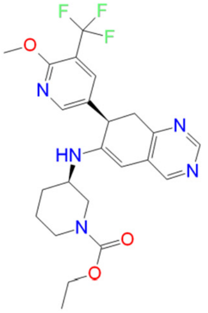	8.16	185	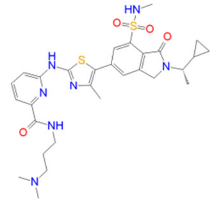	8.2

79	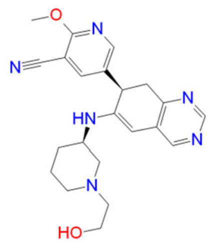	8.32	186	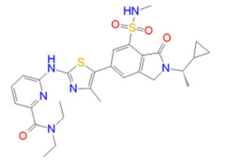	7.9
80	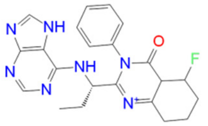	8.92	187	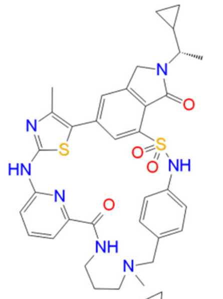	9.1
81	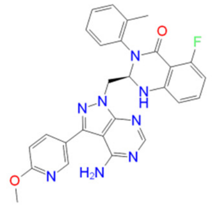	7.50	188	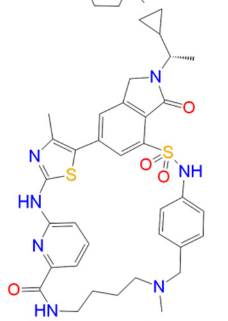	9.0
82	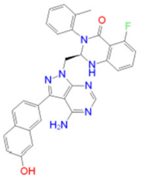	6.64	189	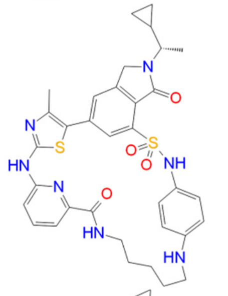	9.2
83	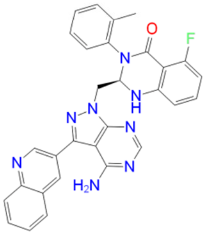	7.06	190	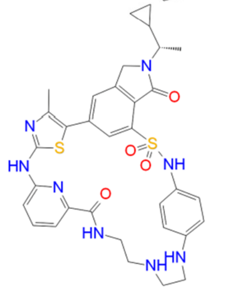	9.3

84	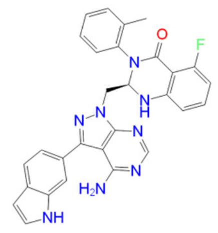	7.79	191	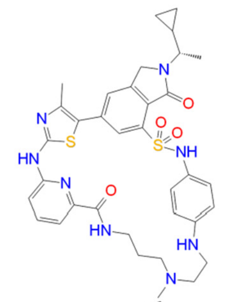	9.3
85	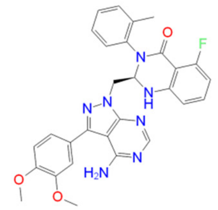	7.58	192	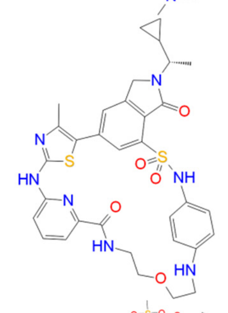	9.2
86	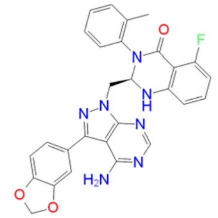	6.97	193	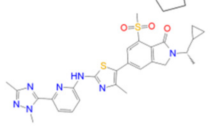	8.8
87	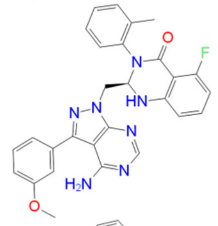	NA	194	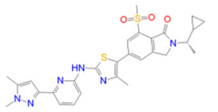	7.6
88	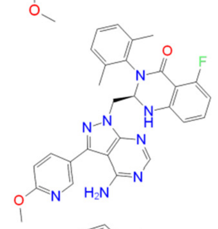	7.38	195	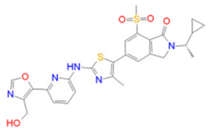	8.7
89	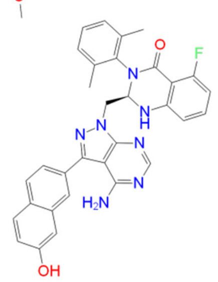	6.38	196	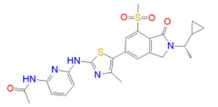	7.8

90	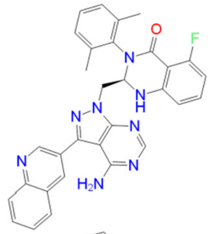	7.09	197	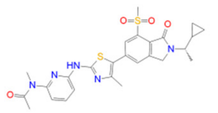	8.9
91	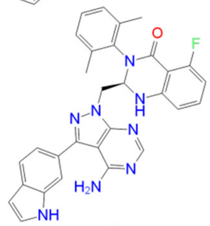	8.06	198	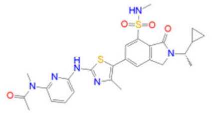	9.1
92	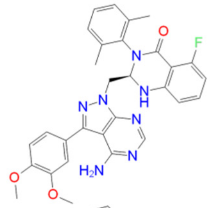	8.07	199	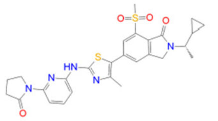	9.2
93	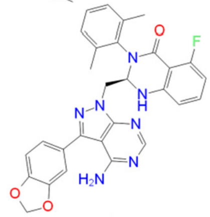	7.07	200	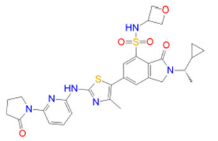	9.1
94	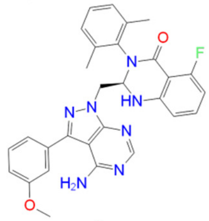	NA	201	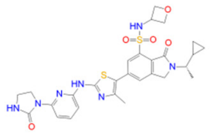	9.2
95	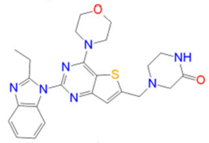	7.69	202	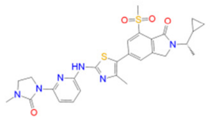	9.1
96	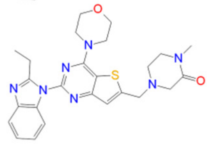	8.30	203	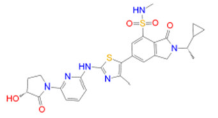	9.3

97	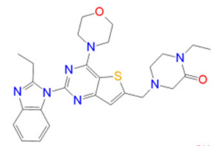	8.38	204	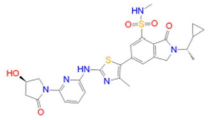	8.9
98	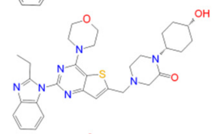	8.24	205	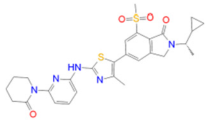	8.9
99	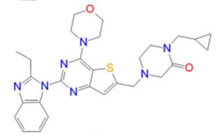	8.60	206	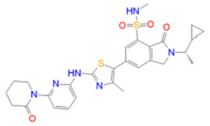	8.9
100	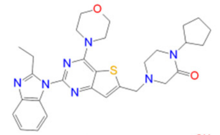	8.67	207	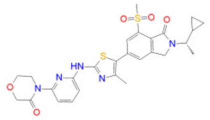	9.0
101	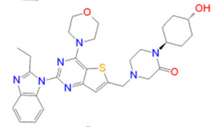	8.67	208	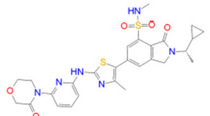	9.2
102	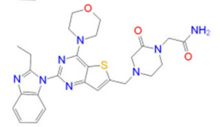	7.92	209	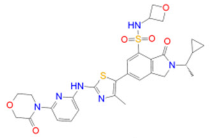	9.0
103	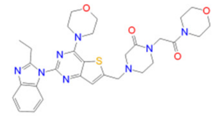	7.82	210	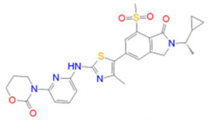	8.9
104	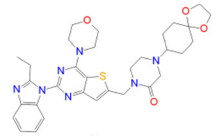	8.65	211	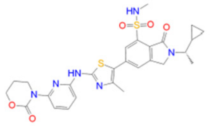	9.2
105	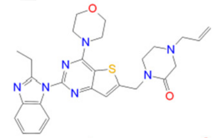	8.79	212	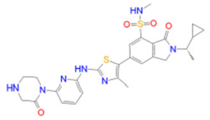	9.1
106	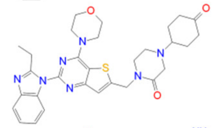	8.79	213	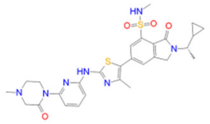	8.8
107	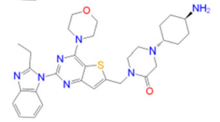	7.85			

#Cpd: Compounds;

Low pIC_{50}		Medium pIC_{50}		High pIC_{50}	
0.918525	C05	0.000614	C01	0.146314	C64
0.003722	C137	0.013614	C66	0.006222	C57
0.00639	C150	0.027467	C193	0.009359	C158
0.020226	C45	0.052375	C118	0.018529	C206
0.031519	C170	0.103259	C101	0.087502	C203
0.037477	C55	0.16035	C129	0.089714	C192
0.041496	C88	0.164582	C109	0.10734	C62
0.041858	C107	0.182944	C55	0.110613	C144
0.076856	C108	0.18718	C121	0.145045	C65
0.101207	C63	0.208749	C173	0.177289	C208
0.204409	C90	0.209223	C132	0.192651	C147
0.21623	C122	0.221449	C174	0.202192	C34
0.222416	C21	0.246496	C100	0.270057	C198
0.243418	C94	0.260394	C38	0.286747	C190
0.2547	C82	0.263985	C111	0.291852	C44
0.272047	C40	0.269492	C142	0.302886	C73
0.339541	C08	0.276823	C117	0.334132	C187

Set_A
Test set

Low pIC_{50}		Medium pIC_{50}		High pIC_{50}	
0.345206	C177	0.292678	C164	0.338574	C53
0.369424	C20	0.32354	C128	0.341411	C161
0.388485	C194	0.342606	C138	0.373802	C209
0.406397	C115	0.460039	C67	0.397538	C169
0.449295	C22	0.464389	C69	0.411461	C197
0.468575	C179	0.501701	C61	0.422677	C205
0.475535	C77	0.507382	C193	0.431534	C30
0.49346	C02	0.514709	C92	0.438346	C164
0.555686	C23	0.568746	C36	0.455366	C49
0.598858	C24	0.574278	C68	0.479663	C159
0.603684	C27	0.609814	C104	0.487579	C207
0.605352	C81	0.610824	C195	0.500929	C201
0.616693	C172	0.629462	C75	0.502511	C46
0.629772	C09	0.637484	C113	0.518507	C143
0.638791	C14	0.652811	C33	0.567239	C43
0.641515	C171	0.659978	C125	0.604859	C199
0.659431	C07	0.687848	C102	0.613666	C200

Set_B
Test set

0.673523	C25	0.688484	C149	**0.621671**	**C212**
0.682578	C29	0.727103	C79	**0.625735**	**C156**
0.694381	C03	0.733326	C74	**0.686265**	**C56**
0.719803	C95	0.737571	C105	**0.725001**	**C160**
0.735129	C10	0.760708	C58	**0.730918**	**C188**
0.736652	C11	0.791139	C64	**0.735259**	**C50**
0.757881	C28	0.805503	C195	**0.736886**	**C134**
0.765128	C31	0.827331	C131	**0.751691**	**C166**
0.771632	C103	0.828515	C123	**0.80953**	**C154**
0.773826	C15	0.834075	C51	**0.844419**	**C42**
0.790251	C19	0.867215	C96	**0.861337**	**C163**
0.794876	C84	0.87571	C97	**0.863844**	**C41**
0.865381	C93	0.889652	C127	**0.87088**	**C52**
0.903904	C181	0.891917	C71	**0.871953**	**C139**
0.905589	C182	0.910552	C151	**0.888896**	**C157**
0.908595	C186	0.928871	C136	**0.914179**	**C210**
0.930403	C83	0.951279	C120	**0.961411**	**C70**

Set_C
Test set

C17	0.072564	C37	0.081994	C162	0.108849
C32	0.163519	C91	0.147734	C204	0.120434
C89	0.167596	C78	0.166277	C80	0.262369
C180	0.173203	C178	0.292771	C112	0.330637
C86	0.295446	C153	0.33685	C167	0.400022
C114	0.317766	C124	0.375261	C59	0.406107
C06	0.340201	C116	0.487657	C202	0.440174
C175	0.494539	C135	0.528554	C211	0.468204
C13	0.518793	C60	0.633794	C189	0.527288
C26	0.530344	C76	0.6488	C146	0.567433
C18	0.54748	C148	0.662125	C191	0.620961
C176	0.700296	C99	0.764531	C141	0.730664
C140	0.707175	C119	0.853411	C47	0.759546
C196	0.710333	C168	0.889161	C145	0.769448
C72	0.733937	C155	0.909226	C152	0.804781
C39	0.886356	C106	0.945141	C165	0.81159
C130	0.890758	C213	0.97804	C54	0.974838

Set_D
Test set

Figure S7. Random sampling table to select the Test set compounds from the dataset of PI3Kδ.

Table S13: Statistical analysis to generate the best CoMSIA model using various combinations of the descriptors field for SET-D compounds.

CoMSIA	q^2	ONC	SEP	r^2	SEE	F-value	Field Contribution				
							S	E	H	A	D
**S**	0.522	5	0.669	0.612	0.607	47.725	100	-	-	-	-
**E**	0.358	4	0.778	0.548	0.653	45.991	-	100	-	-	-
**H**	0.504	4	0.685	0.612	0.605	59.974	-	-	100	-	-
**A**	0.481	5	0.702	0.613	0.606	47.807	-	-	-	100	-
**D**	0.473	5	0.707	0.643	0.582	54.347	-	-	-	-	100
**SE**	0.504	4	0.684	0.570	0.637	50.392	68.8	31.2	-	-	-
**EH**	0.496	5	0.692	0.615	0.604	48.336	-	28.1	71.9	-	-
**EA**	0.469	6	0.712	0.631	0.594	42.693	-	37.4	-	62.6	-
**ED**	0.468	3	0.706	0.568	0.636	67.066		27.6	-	-	72.4
**SH**	0.513	4	0.678	0.614	0.603	60.531	43.7	-	56.3	-	-
**SA**	0.533	5	0.666	0.660	0.568	58.626	47.9	-	-	52.1	-
**SD**	0.490	4	0.693	0.636	0.585	66.625	45.5	-	-	-	54.5
**HA**	0.521	4	0.672	0.630	0.591	64.594	-	-	57.9	42.1	-
**HD**	0.493	2	0.687	0.579	0.626	105.741	-	-	48.2	-	51.8
**AD**	0.510	4	0.680	0.666	0.561	75.750	-	-	-	44.3	55.7
**SHE**	0.513	4	0.678	0.608	0.608	58.842	36.6	18.3	45.1	-	-
**SEA**	0.528	5	0.670	0.627	0.595	50.837	40.2	19.1	-	40.6	-
**SED**	0.489	3	0.692	0.583	0.625	71.322	37.1	17.2	-	-	45.7
**EHA**	0.516	4	0.678	0.625	0.595	63.347	-	17.0	48.8	34.3	-
**EHD**	0.493	3	0.689	0.599	0.613	76.216	-	13.9	42.1	-	44.0
**SHA**	0.526	4	0.669	0.641	0.582	67.720	29.0	-	38.3	32.7	-
**SHD**	0.500	3	0.685	0.616	0.600	81.669	28.2	-	34.4	-	37.4
**EAD**	0.506	5	0.685	0.683	0.549	64.929	-	15.5	-	37.0	47.5
**HAD**	0.509	3	0.678	0.636	0.584	89.050	-	-	34.8	28.4	36.8
**SEHD**	0.500	3	0.85	0.605	0.608	78.264	25.1	11.5	29.9	-	33.5
**SEHA**	0.524	4	0.670	0.629	0.591	64.497	26.0	12.5	33.2	28.3	-
**SEAD**	0.511	4	0.679	0.649	0.575	70.274	25.9	11.4	-	29.0	33.8
**EHAD**	0.508	3	0.679	0.629	0.590	86.292	-	10.4	31.2	24.4	34.0
**SHAD**	0.509	3	0.679	0.641	0.580	90.887	20.4	-	26.9	24.1	28.6
**SEHAD**	0.512	3	0.676	0.628	0.590	86.145	18.9	9.3	24.2	20.6	26.9

q^2: squared cross-validated correlation coefficient; **ONC**: optimal number of components; **SEP**: standard error of prediction; r^2: squared correlation coefficient; **SEE**: standard error of estimation; **F-value**: F-test value; r^2_{pred}: predictive r^2; **S**: Steric; **E**: Electrostatic; **H**: Hydrophobic; **A**: H-bond acceptor; **D**: H-bond donor. Green highlighted box showing the final selection of the CoMSIA model.

Table S14: Actual pIC_{50} vs. Predicted pIC_{50} of the compounds from the CoMFA and CoMSIA training set compounds of SET-D.

#Cpd	Actual pIC_{50}	CoMFA		CoMSIA	
		Predicted pIC_{50}	Residuals	Predicted pIC_{50}	Residuals
C17	5.65	6.507	-0.857	6.689	-1.039
C32	5.76	6.701	-0.941	6.731	-0.971
C89	6.38	6.69	-0.31	7.626	-1.246
C180	6.8	7.5	-0.7	7.551	-0.751
C86	6.97	7.597	-0.627	7.527	-0.557
C114	7.08	8.135	-1.055	8.443	-1.363
C06	7.27	7.251	0.019	7.143	0.127
C175	7.3	7.646	-0.346	7.67	-0.37
C13	7.34	6.855	0.485	7.155	0.185
C26	7.46	6.514	0.946	6.734	0.726
C18	7.53	6.613	0.917	6.726	0.804
C176	7.6	7.797	-0.197	7.828	-0.228
C140	7.65	8.711	-1.061	8.634	-0.984
C196	7.8	8.6	-0.8	8.667	-0.867
C72	7.84	8.361	-0.521	8.282	-0.442
C39	7.85	8.792	-0.942	8.799	-0.949
C130	7.92	8.382	-0.462	8.816	-0.896
C37	7.95	8.792	-0.842	8.632	-0.682
C91	8.06	7.45	0.61	7.525	0.535
C78	8.16	8.328	-0.168	8.257	-0.097
C178	8.2	7.487	0.713	7.57	0.63
C153	8.29	9.355	-1.065	9.19	-0.9
C124	8.31	8.379	-0.069	8.392	-0.082
C116	8.34	8.403	-0.063	8.369	-0.029
C135	8.38	8.395	-0.015	8.527	-0.147
C60	8.44	8.993	-0.553	8.784	-0.344
C76	8.49	8.365	0.125	8.299	0.191
C148	8.52	8.701	-0.181	9.129	-0.609
C99	8.6	8.299	0.301	8.046	0.554
C119	8.65	8.41	0.24	8.375	0.275
C168	8.69	9.403	-0.713	8.951	-0.261
C155	8.72	9.353	-0.633	9.506	-0.786
C106	8.79	8.333	0.457	8.311	0.479
C213	8.8	8.685	0.115	8.667	0.133
C162	8.88	9.746	-0.866	9.663	-0.783
C204	8.9	8.81	0.09	8.724	0.176

C80	8.92	8.207	0.713	8.806	0.114
C112	8.95	8.321	0.629	8.444	0.506
C167	9	8.556	0.444	8.99	0.01
C59	9.04	9.005	0.035	8.772	0.268
C202	9.1	8.645	0.455	8.597	0.503
C211	9.2	8.749	0.451	8.588	0.612
C189	9.2	9.482	-0.282	9.63	-0.43
C146	9.22	9.493	-0.273	9.248	-0.028
C191	9.3	9.521	-0.221	9.701	-0.401
C141	9.3	8.743	0.557	9.033	0.267
C47	9.52	8.854	0.666	8.233	1.287
C145	9.69	9.004	0.686	9.164	0.526
C152	9.69	9.273	0.417	9.069	0.621
C165	9.69	9.274	0.416	9.512	0.178
C54	9.88	8.965	0.915	8.775	1.105
C05	5.62	6.69	-1.07	6.641	-1.021
C25	5.65	6.336	-0.686	6.482	-0.832
C21	5.75	6.6	-0.85	6.769	-1.019
C24	5.81	6.317	-0.507	6.669	-0.859
C27	6.06	6.49	-0.43	6.667	-0.607
C07	6.21	7.141	-0.931	7.255	-1.045
C22	6.27	6.593	-0.323	6.578	-0.308
C170	6.5	6.84	-0.34	6.791	-0.291
C45	6.61	7.615	-1.005	7.015	-0.405
C82	6.64	7.488	-0.848	7.653	-1.013
C171	6.8	6.812	-0.012	6.157	0.643
C181	6.8	7.466	-0.666	7.27	-0.47
C23	6.8	6.483	0.317	6.732	0.068
C63	6.82	7.731	-0.911	7.448	-0.628
C31	7	8.647	-1.647	8.75	-1.75
C83	7.06	7.41	-0.35	7.457	-0.397
C20	7.07	6.569	0.501	6.684	0.386
C93	7.07	7.564	-0.494	7.499	-0.429
C90	7.09	7.424	-0.334	7.427	-0.337
C03	7.21	7.296	-0.086	7.17	0.04
C14	7.27	7.002	0.268	7.191	0.079
C12	7.29	6.999	0.291	7.267	0.023
C172	7.3	7.505	-0.205	7.598	-0.298
C182	7.3	8.302	-1.002	8.708	-1.408
C94	7.3	7.197	0.103	7.351	-0.051
C02	7.31	7.392	-0.082	7.24	0.07
C137	7.37	8.338	-0.968	8.367	-0.997
C88	7.38	7.356	0.024	7.298	0.082
C11	7.39	7.116	0.274	7.103	0.287
C40	7.4	8.518	-1.118	8.745	-1.345
C09	7.48	7.062	0.418	7.118	0.362

C19	7.49	6.704	0.786	6.66	0.83
C81	7.5	7.351	0.149	7.326	0.174
C35	7.52	8.73	-1.21	8.418	-0.898
C77	7.56	7.684	-0.124	8.022	-0.462
C55	7.58	7.619	-0.039	7.479	0.101
C08	7.58	7.063	0.517	7.091	0.489
C194	7.6	8.291	-0.691	8.478	-0.878
C179	7.6	7.498	0.102	7.583	0.017
C150	7.65	8.145	-0.495	8.576	-0.926
C95	7.69	8.347	-0.657	8.131	-0.441
C28	7.72	7.007	0.713	6.771	0.949
C10	7.74	6.945	0.795	7.156	0.584
C15	7.76	6.778	0.982	6.733	1.027
C84	7.79	7.525	0.265	7.552	0.238
C177	7.8	7.778	0.022	7.675	0.125
C103	7.82	8.163	-0.343	7.873	-0.053
C122	7.82	8.281	-0.461	8.317	-0.497
C108	7.85	8.133	-0.283	8.158	-0.308
C107	7.85	8.263	-0.413	8.252	-0.402
C29	7.85	7.119	0.731	6.835	1.015
C115	7.88	7.16	0.72	7.609	0.271
C186	7.9	7.994	-0.094	8.325	-0.425
C121	7.92	8.308	-0.388	8.334	-0.414
C102	7.92	8.296	-0.376	7.861	0.059
C149	7.95	8.62	-0.67	8.45	-0.5
C174	8	7.645	0.355	7.657	0.343
C67	8.02	8.665	-0.645	8.397	-0.377
C71	8.05	8.06	-0.01	8.09	-0.04
C92	8.07	7.623	0.447	7.451	0.619
C33	8.09	8.781	-0.691	8.742	-0.652
C128	8.1	8.474	-0.374	8.468	-0.368
C66	8.11	8.687	-0.577	8.291	-0.181
C123	8.2	8.395	-0.195	8.48	-0.28
C117	8.2	8.4	-0.2	8.393	-0.193
C109	8.2	8.364	-0.164	8.345	-0.145
C195	8.2	7.926	0.274	8.197	0.003
C01	8.22	8.508	-0.288	8.918	-0.698
C98	8.24	8.273	-0.033	8.233	0.007
C96	8.3	8.355	-0.055	8.099	0.201
C142	8.3	8.543	-0.243	8.62	-0.32
C193	8.3	8.044	0.256	8.176	0.124
C136	8.31	8.374	-0.064	8.52	-0.21
C79	8.32	8.303	0.017	8.44	-0.12
C132	8.35	8.423	-0.073	8.467	-0.117
C51	8.36	8.879	-0.519	8.75	-0.39
C97	8.38	8.361	0.019	8.075	0.305

C164	8.4	7.993	0.407	8.205	0.195
C138	8.4	8.365	0.035	8.567	-0.167
C74	8.4	8.418	-0.018	8.223	0.177
C125	8.42	8.318	0.102	8.331	0.089
C69	8.43	8.512	-0.082	8.451	-0.021
C110	8.44	8.329	0.111	8.284	0.156
C113	8.46	8.289	0.171	8.106	0.354
C111	8.49	8.358	0.132	8.468	0.022
C75	8.49	8.422	0.068	8.447	0.043
C173	8.5	7.796	0.704	7.845	0.655
C129	8.53	8.517	0.013	8.484	0.046
C120	8.53	8.385	0.145	8.33	0.2
C68	8.6	8.704	-0.104	8.428	0.172
C36	8.6	8.791	-0.191	8.761	-0.161
C104	8.65	8.279	0.371	8.593	0.057
C131	8.65	8.46	0.19	8.491	0.159
C101	8.67	8.263	0.407	8.019	0.651
C100	8.67	8.338	0.332	8.127	0.543
C118	8.67	8.439	0.231	8.353	0.317
C127	8.69	8.443	0.247	8.448	0.242
C55	8.69	8.919	-0.229	8.763	-0.073
C195	8.7	8.479	0.221	8.883	-0.183
C58	8.72	8.97	-0.25	8.589	0.131
C151	8.74	8.084	0.656	8.571	0.169
C38	8.74	8.739	0.001	8.811	-0.071
C105	8.79	8.393	0.397	8.345	0.445
C61	8.79	8.951	-0.161	8.786	0.004
C193	8.8	8.276	0.524	8.553	0.247
C64	8.82	8.993	-0.173	8.784	0.036
C126	8.85	8.515	0.335	8.46	0.39
C166	8.85	8.61	0.24	9.061	-0.211
C70	8.88	8.571	0.309	8.355	0.525
C210	8.9	8.651	0.249	8.566	0.334
C205	8.9	8.666	0.234	8.672	0.228
C197	8.9	8.691	0.209	8.705	0.195
C49	8.92	8.757	0.163	8.75	0.17
C46	8.92	8.861	0.059	8.517	0.403
C134	8.92	8.437	0.483	8.485	0.435
C207	9	8.649	0.351	8.866	0.134
C206	9	8.775	0.225	8.693	0.307
C139	9	8.865	0.135	8.669	0.331
C30	9	9.015	-0.015	8.711	0.289
C53	9	8.729	0.271	8.751	0.249
C188	9	9.498	-0.498	9.69	-0.69
C209	9	9	0	9.211	-0.211
C52	9.04	8.858	0.182	8.772	0.268

C200	9.1	9.028	0.072	9.052	0.048
C198	9.1	8.805	0.295	8.726	0.374
C187	9.1	9.412	-0.312	9.712	-0.612
C212	9.1	8.759	0.341	8.706	0.394
C73	9.15	8.627	0.523	8.493	0.657
C199	9.2	8.712	0.488	8.681	0.519
C201	9.2	9.026	0.174	8.9	0.3
C192	9.2	9.473	-0.273	9.646	-0.446
C208	9.2	8.748	0.452	8.888	0.312
C143	9.22	8.792	0.428	9.076	0.144
C62	9.26	9.037	0.223	8.785	0.475
C190	9.3	9.454	-0.154	9.603	-0.303
C169	9.3	9.396	-0.096	9.026	0.274
C43	9.3	8.742	0.558	9.037	0.263
C203	9.3	8.766	0.534	8.822	0.478
C65	9.32	8.937	0.383	8.763	0.557
C57	9.34	8.969	0.371	8.762	0.578
C164	9.39	9.204	0.186	9.44	-0.05
C34	9.39	8.75	0.64	8.75	0.64
C157	9.39	9.434	-0.044	9.424	-0.034
C44	9.39	8.788	0.602	8.831	0.559
C161	9.52	9.761	-0.241	9.351	0.169
C147	9.52	9.451	0.069	9.269	0.251
C42	9.52	8.837	0.683	8.72	0.8
C156	9.52	9.691	-0.171	9.59	-0.07
C144	9.69	8.888	0.802	9.086	0.604
C160	9.69	9.801	-0.111	9.317	0.373
C154	9.69	9.509	0.181	9.577	0.113
C159	9.69	8.809	0.881	9.137	0.553
C158	9.69	9.57	0.12	9.555	0.135
C163	9.69	9.691	-0.001	9.654	0.036
C56	9.82	8.96	0.86	8.763	1.057
C41	10	8.851	1.149	9.783	0.217
C50	10	8.909	1.091	8.964	1.036

#Cpd: Compounds

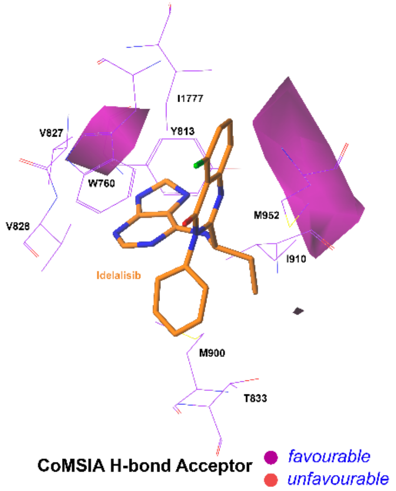

Figure S8. The standard deviation of coefficient contour maps was obtained from the CoMSIA model of PI3Kδ. The contour maps indicate the favorable and unfavorable substitution for hydrophobic, H-bond acceptor, and H-bond donor groups over the compound idelalisib.

Table S15: Newly designed compounds and their predicted pIC_{50} values against PI3Kγ and PI3Kδ.

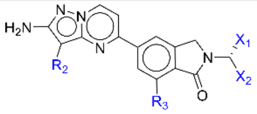

Structure

#Designed_Cpd	R_2	R_3	X_1	X_2	pIC_{50} (PI3Kγ)	pIC_{50} (PI3Kδ)
01	H	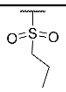	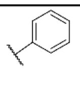	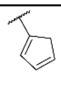	7.63	7.58
02	H	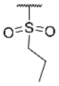	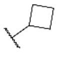	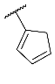	8.03	7.58
03	H	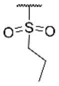	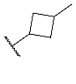	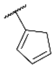	8.00	7.58
04	H	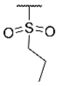	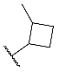	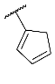	8.02	7.58
05	H	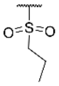	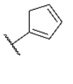	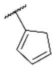	7.91	7.58
06	H	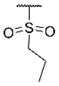	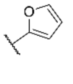	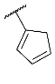	7.83	7.57
07	H	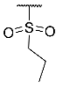	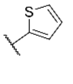	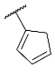	7.85	7.57
08	H	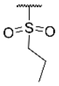	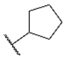	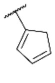	8.04	7.58
09	H	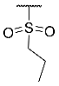	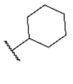	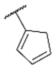	8.00	7.58

10	H 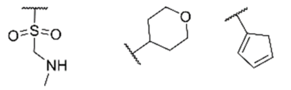	7.93	7.60
11	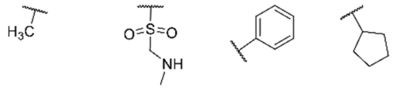	7.97	7.64
12	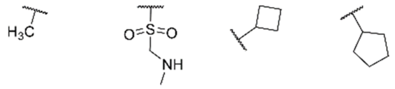	8.48	7.61
13	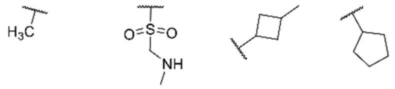	8.37	7.60
14	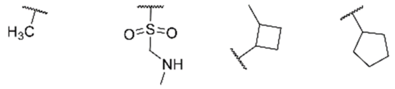	8.39	7.60
15	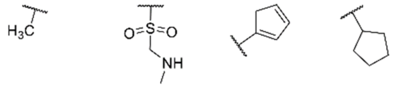	8.30	7.61
16	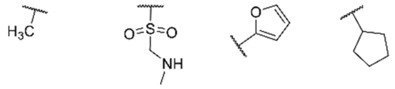	8.13	7.63
17	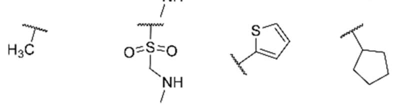	8.17	7.64
18	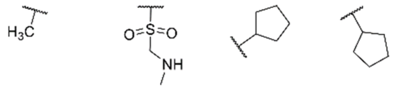	8.33	7.64
19	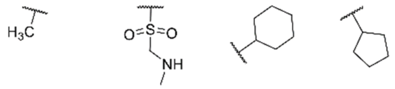	8.39	7.61
20	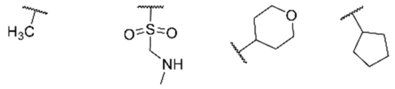	8.31	7.66
21	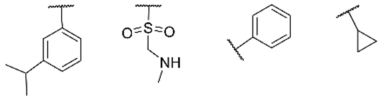	9.42	7.60
22	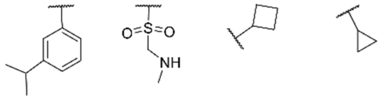	9.38	7.69
23	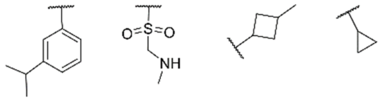	9.36	7.68
24	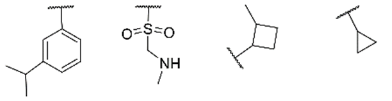	9.34	7.70
25	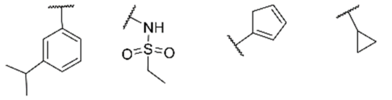	9.11	7.66
26	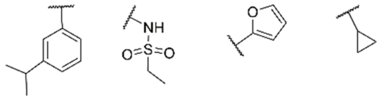	9.21	7.68
27	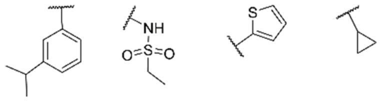	9.17	7.67
28	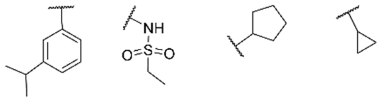	8.80	7.63
29	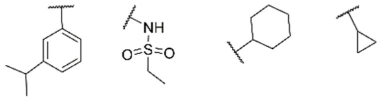	8.65	7.66

30	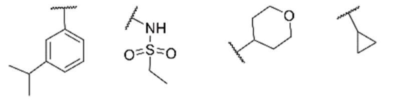	8.76	7.67
31	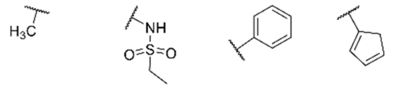	8.35	7.66
32	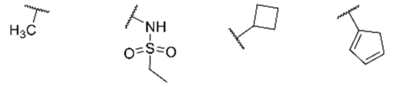	8.46	7.66
33	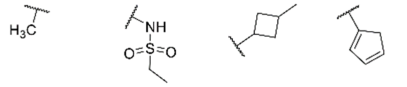	8.46	7.68
34	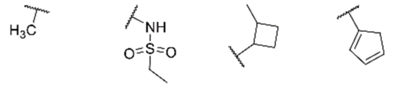	8.33	7.67
35	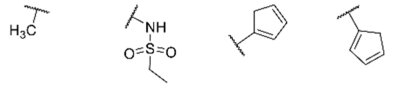	8.40	7.65
36	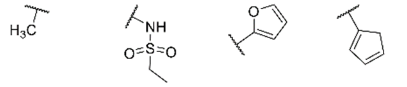	8.48	7.66
37	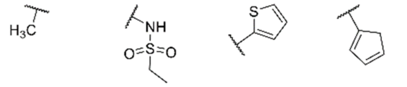	8.49	7.65
38	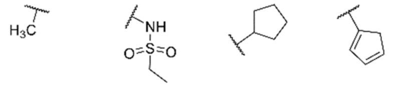	8.50	7.68
39	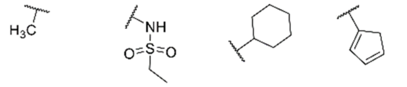	8.57	7.68
40	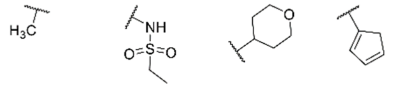	8.68	7.68
41	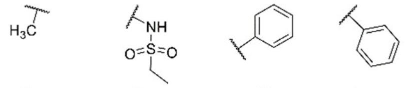	8.41	7.71
42	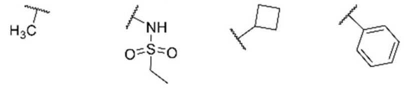	8.40	7.62
43	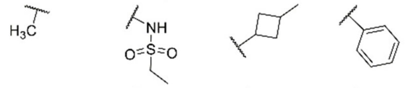	8.37	7.54
44	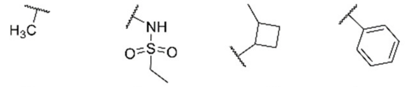	8.39	7.62
45	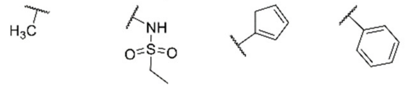	8.36	7.64
46	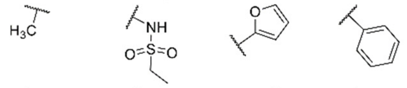	8.48	7.61
47	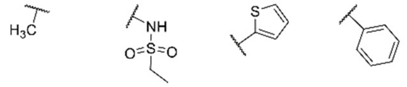	8.38	7.62
48	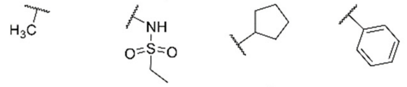	8.20	7.60
49	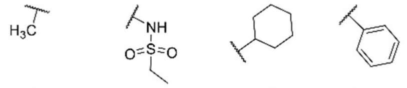	8.81	7.82
50	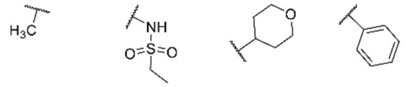	8.91	7.82
51	H 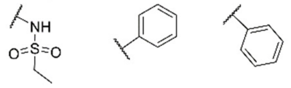	8.06	7.71

52	H	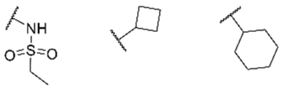	8.23	7.61
53	H	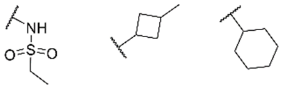	8.19	7.55
54	H	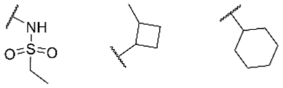	8.22	7.62
55	H	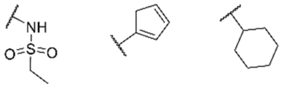	8.18	7.63
56	H	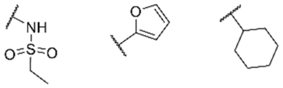	8.20	7.61
57	H	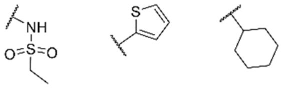	8.19	7.61
58	H	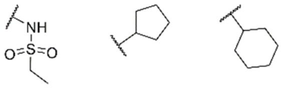	8.01	7.60
59	H	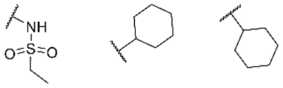	8.63	7.81
60	H	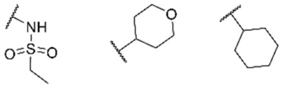	8.63	7.82
61	H	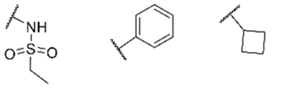	7.96	7.67
62	H	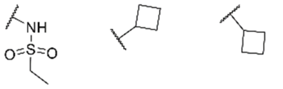	8.19	7.67
63	H	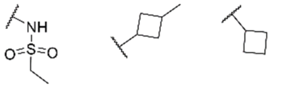	8.13	7.60
64	H	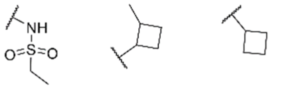	8.23	7.68
65	H	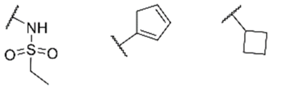	7.94	7.63
66	H	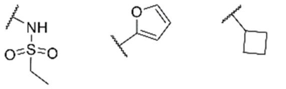	8.12	7.65
67	H	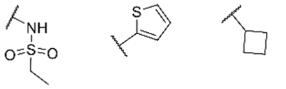	8.08	7.63
68	H	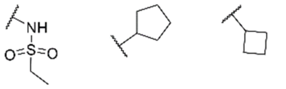	7.91	7.60
69	H	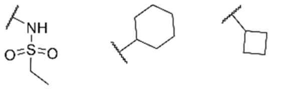	7.96	7.56
70	H	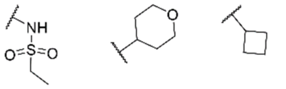	8.11	7.58
71	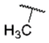	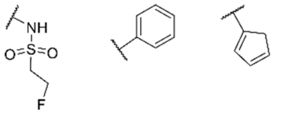	8.35	7.67
72	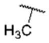	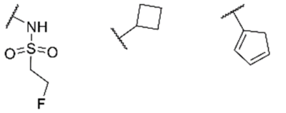	8.47	7.66

73	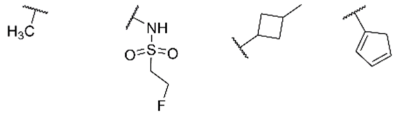	8.48	7.68
74	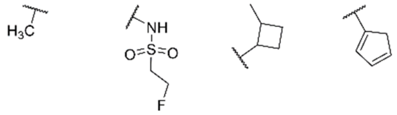	8.34	7.67
75	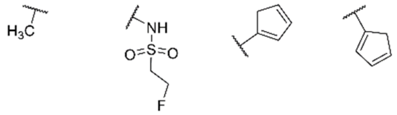	8.41	7.65
76	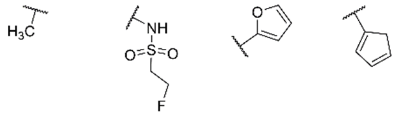	8.41	7.66
77	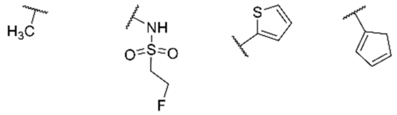	8.44	7.66
78	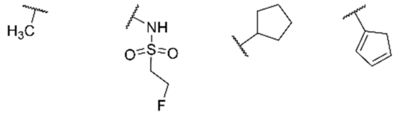	8.51	7.68
79	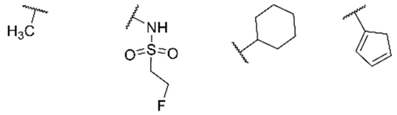	8.57	7.69
80	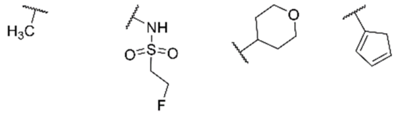	8.59	7.69
81	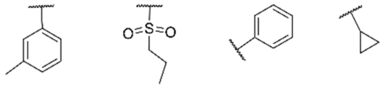	9.30	7.57
82	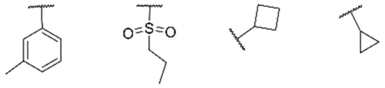	9.49	7.65
83	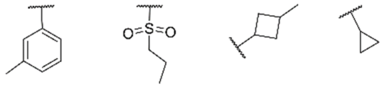	9.46	7.64
84	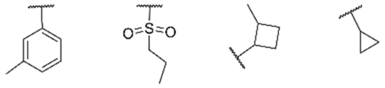	9.45	7.66
85	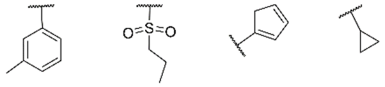	9.36	7.60
86	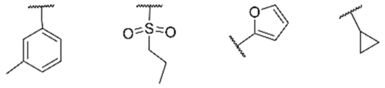	9.09	7.61
87	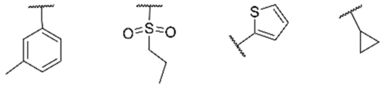	9.39	7.27
88	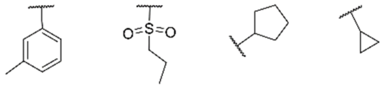	9.05	7.56
89	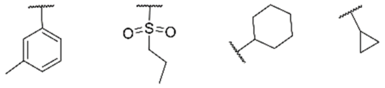	8.86	7.60
90	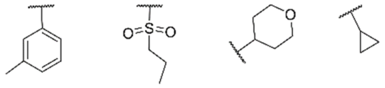	8.87	7.61
91	H 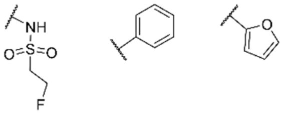	8.25	7.72

92	H	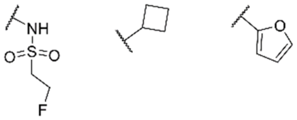	8.36	7.71
93	H	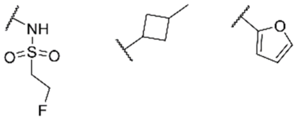	8.36	7.73
94	H	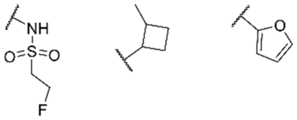	8.25	7.73
95	H	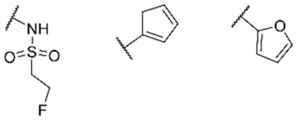	8.27	7.71
96	H	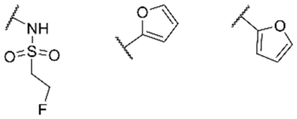	8.28	7.72
97	H	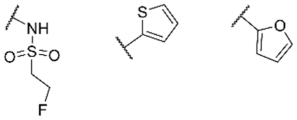	8.31	7.71
98	H	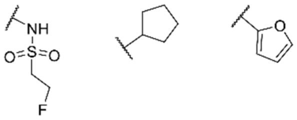	8.39	7.74
99	H	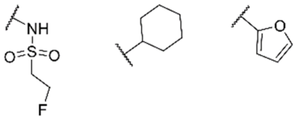	8.47	7.74
100	H	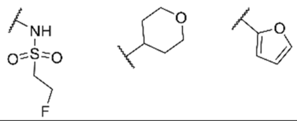	8.48	7.74

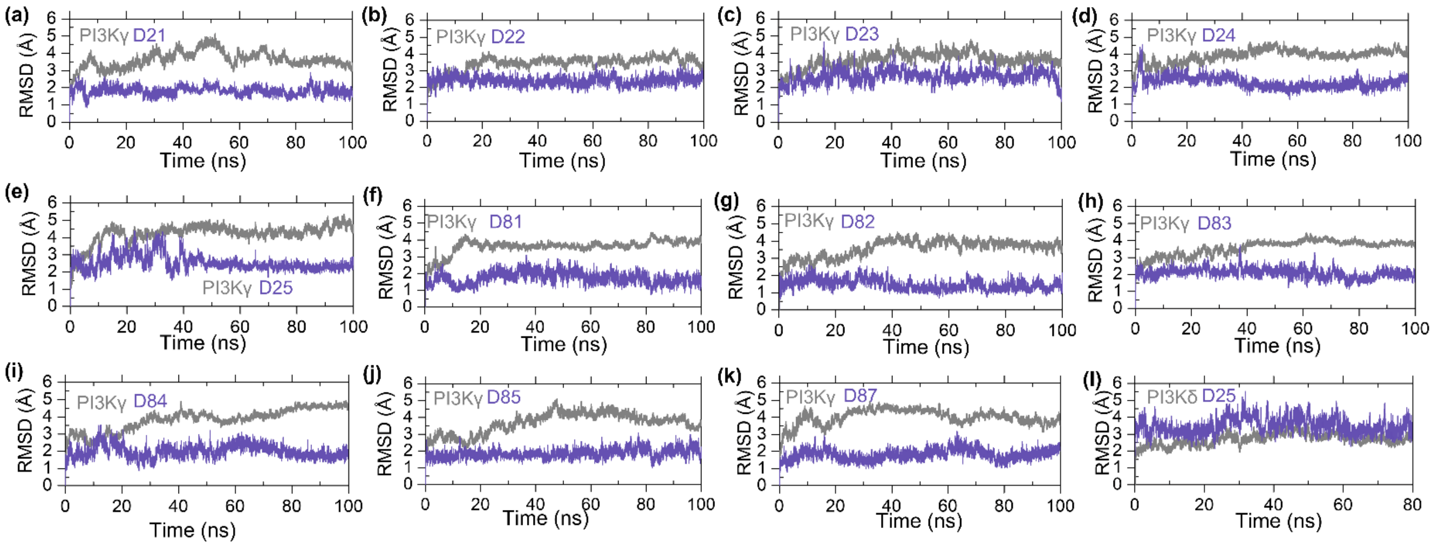

Figure S9. RMSD plots from molecular dynamics simulation study of the receptor and designed compounds. The RMSDs of the ligand and α-carbon of the receptors were shown in slate and grey.
